# Evaluation of cardiac pro-arrhythmic risks using the artificial neural network with ToR–ORd *in silico* model output

**DOI:** 10.3389/fphys.2024.1374355

**Published:** 2024-04-04

**Authors:** Nurul Qashri Mahardika T, Ali Ikhsanul Qauli, Aroli Marcellinus, Ki Moo Lim

**Affiliations:** ^1^ Computational Medicine Lab, Department of IT Convergence Engineering, Kumoh National Institute of Technology, Gumi, Republic of Korea; ^2^ Department of Engineering, Faculty of Advanced Technology and Multidiscipline, Universitas Airlangga, Surabaya, Jawa Timur, Indonesia; ^3^ Computational Medicine Lab, Department of Medical IT Convergence Engineering, Kumoh National Institute of Technology, Gumi, Republic of Korea; ^4^ Meta Heart Co Ltd., Gumi, Republic of Korea

**Keywords:** torsade de pointes, Tomek–O’Hara Rudy ventricular *in silico* cell model, artificial neural networks, grid search, explainable artificial intelligence

## Abstract

Torsades de pointes (TdP) is a type of ventricular arrhythmia that can lead to sudden cardiac death. Drug-induced TdP has been an important concern for researchers and international regulatory boards. The Comprehensive *in vitro* Proarrhythmia Assay (CiPA) initiative was proposed that integrates *in vitro* testing and computational models of cardiac ion channels and human cardiomyocyte cells to evaluate the proarrhythmic risk of drugs. The TdP risk classification performance using only a single TdP metric may require some improvements because of information limitations and the instability of generalizing results. This study evaluates the performance of TdP metrics from the *in silico* simulations of the Tomek–O'Hara Rudy (ToR–ORd) ventricular cell model for classifying the TdP risk of drugs. We utilized these metrics as an input to an artificial neural network (ANN)-based classifier. The ANN model was optimized through hyperparameter tuning using the grid search (GS) method to find the optimal model. The study outcomes show an area under the curve (AUC) value of 0.979 for the high-risk category, 0.791 for the intermediate-risk category, and 0.937 for the low-risk category. Therefore, this study successfully demonstrates the capability of the ToR–ORd ventricular cell model in classifying the TdP risk into three risk categories, providing new insights into TdP risk prediction methods.

## 1 Introduction

The development of pharmaceuticals is crucial due to the potential risk of Torsades de Pointes (TdP). For that reason, in the 1990s–2000s, several drugs were withdrawn from the market due to their tendency to induce TdP (Wood and Roden, 2004; Sager et al., 2014). The potential risk associated with TdP occurs because of the pharmacological inhibition of human ether-a-go-go-related gene (hERG) ion channels, which regulate the rapid component of the delayed rectifier current (IKr) and prolong the QT interval during drug administration, and it may potentially be life-threatening to consumers. Responding to these concerns, the International Council for Harmonization (ICH) established the E14 guidelines for the clinical evaluation and the S7B guidelines for the non-clinical assessment of drug cardiotoxicity and ventricular repolarization (Cavero and Crumb, 2005; Crumb et al., 2016).

Nevertheless, these guidelines have limitations, including the need for detailed clinical trials and having a high level of sensitivity with low specificity, which may obstruct the development of therapeutic drugs capable of prolonging the QT interval without inducing TdP (Llopis-Lorente et al., 2020). The Comprehensive *in vitro* Proarrhythmia Assay (CiPA) initiative was proposed by the Food and Drug Administration (FDA) during a Think Tank meeting in the United States in July 2013. It involves four working groups focusing on *in vitro* ion channel studies, *in silico* modeling of ion channel study results, cardiomyocyte studies, and searching for biomarkers other than QTc in human electrocardiography (ECG) (Crumb et al., 2016).

The development of the O’Hara Rudy (ORd) ventricular cell model was observed in disease-free human ventricular cells from a wealth of experimental data obtained from healthy individuals (O’Hara et al., 2011). A previous study introduced and applied the dynamic hERG pharmacological model to the ORd model (IKr-dyn ORd model) (Li et al., 2017). Furthermore, a follow-up study improved the ORd model with the dynamic hERG by rescaling some major ionic currents, including IKs, L-type calcium current (ICaL), IKr, sodium current (INa), and inward rectifier current (IK1) (CiPAORdv1.0 model) (Dutta et al., 2017). The ventricular cell model enables the simulation of cellular responses to a range of stimuli or conditions that potentially induce TdP, offering a deeper insight into how alterations at the cellular level can impact the risk of arrhythmias like TdP. This modeling approach allows for the generation of various *in silico* biomarkers from action potential (AP), calcium concentration (Ca), and net charges (qNet and qInward).

Several studies have applied various TdP metrics from the ORd or CiPAORdv1.0 ventricular cell model to classify TdP risks (Mirams et al., 2011; Lancaster and Sobie, 2016; Passini et al., 2017; Li et al., 2019; Yoo et al., 2021; Jeong et al., 2022). In their study, Mirams et al. (2011) utilized the ventricular cell model developed by Grandi et al. (2010). APD_90_ was the reference parameter for assessing APD_50_, APD_90_, triangulation, and maximum restitution. In the machine learning process, APD_50_, APD_90_, triangulation of the action potential duration (APD), and maximum restitution of APD were used. Nevertheless, the integration of these parameters resulted in marginally enhanced leave-one-out cross-validation scores that may be attributable to the potential occurrence of overfitting in the model.

The FDA proposed using qNet and qInward, which were calculated through *in silico* simulations that show promising metrics for categorizing the proarrhythmic risk of drugs (Strauss et al., 2019). A previous study suggested qNet as a single metric from the CiPAORdv1.0 ventricular cell model to classify TdP risk into high- and low-risk categories using ordinal logistic regression (Li et al., 2019). In addition, a study proposed the utilization of 13 electrophysiological features (upstroke velocity, peak voltage, ${\text{APD}}_{50}$ , APD at −60 mV, ${\text{APD}}_{90}$ , resting voltage, AP triangulation, diastolic $\left[{Ca2}^{+}\right]i$ , amplitude of ${\text{Ca}}_{\text{transient}}$ , peak $\left[{Ca2}^{+}\right]i$ , ${\text{CaD}}_{50}$ , ${\text{CaD}}_{90}$ , and Catri) as appropriate parameters in implementing statistical and machine learning models to perform the binary classification of various drugs into two categories: TdP+ (posing a risk of TdP) or TdP− (lacking TdP risk) (Parikh et al., 2017).

A dataset comprising 86 drugs from the studies conducted by Mirams et al. (2011) and Kramer et al. (2013) has been used as a computational methodology that integrates simulations of drug effects on cardiac dynamics with statistical analyses and machine learning techniques to classify TdP risks. The combination of drug simulation data and statistical analysis employing the support vector machine (SVM) algorithm shows good performance, where the AUC value is 0.86. This approach relies on metrics computed from the action potential duration at 90% repolarization ( ${\text{APD}}_{90}$ ) and the ${\text{Ca}}_{\text{resting}}$ waveform. This approach delivers superior risk prediction capabilities by incorporating insights derived from cardiac cells (Lancaster and Sobie, 2016).

Furthermore, the Tomek–O’Hara Rudy (ToR–ORd) ventricular cell model proposed by Tomek et al. (2019) represents an improvement over the ORd ventricular cell model. The ORd cell model shows that the AP plateau exhibits a higher value than the experimental data employed. The dynamics of AP duration accommodation to heart rate acceleration or sodium behavior demonstrates limited agreement (Tomek et al., 2019). Therefore, the ToR–ORd ventricular cell model revised equations for the ICaL, calcium-sensitive chloride current I(Ca)Cl, chloride background current (IClb), INa, IK1, and IKr to replicate AP waveforms (Tomek et al., 2019). The ICaL revision was based on the Goldman–Hodgkin–Katz (GHK) flux equation with the ionic activity coefficient derived from the Davies equation and Debye–Huckel theory, according to the studies by Magyar et al. (2000), and INa was substituted with an alternative formulation derived by Grandi et al. (2010), providing a more precise representation of sodium current behavior in human ventricular myocardial cells (Grandi et al., 2010; Tomek et al., 2019). Moreover, the ToR–ORd ventricular cell model modified INa to incorporate changes resulting from CaMKII phosphorylation, a critical regulatory mechanism in cardiac electrophysiology (Tomek et al., 2019). The ToR–ORd model was developed through a comprehensive calibration and validation strategy, encompassing rigorous calibration criteria (Han et al., 2020). Concurrently, the authors found that calcium concentrations and sodium homeostasis remain constant under conditions simulating high potential, which is consistent with available experimental data.

A recent study validated 12 *in silico* features, i.e., 6 AP features $({\text{APD}}_{50}$ , ${\frac{\text{dVm}}{\text{dt}}}_{\max}$ , ${\frac{\text{dVm}}{\text{dt}}}_{\max\;\_\text{repol}}{\text{Vm}}_{\text{peak}}$ , ${\text{APD}}_{90}$ , ${\text{APD}}_{50}$ , and ${\text{APD}}_{\text{tri}})$ , 4 calcium features ( ${\text{Ca}}_{\text{peak}}$ ; ${\text{CaD}}_{90}$ ; ${\text{CaD}}_{50}$ ; and ${\text{CaD}}_{\text{tri}}$ ), and 2 net charge features (qInward and qNet), derived from the ToR–ORd ventricular cell model affected by drugs (Jeong et al., 2022). The study classified TdP risk (high, intermediate, and low) using ordinal logistic regression (OLR) for each TdP metric. However, using a single TdP metric demonstrated low performance. This is attributed to the complexity of TdP phenomena involving various aspects of cellular electrophysiology, rendering a single feature insufficient to encompass all relevant variations and interactions in TdP risks.

In this study, we assess the diversity of *in silico* metrics for TdP, encompassing various aspects of cellular electrophysiological changes induced by drug influence. Consequently, the primary objective of this research is to leverage the nine available features from the ToR–ORd ventricular cell model to classify TdP risk. This is achieved by using an optimized artificial neural network (ANN) through the grid search (GS) process for hyperparameter tuning and integrating explainable artificial intelligence (XAI) with SHapley Additive exPlanation (SHAP) values into the analysis.

## 2 Research methods

**FIGURE 1:**
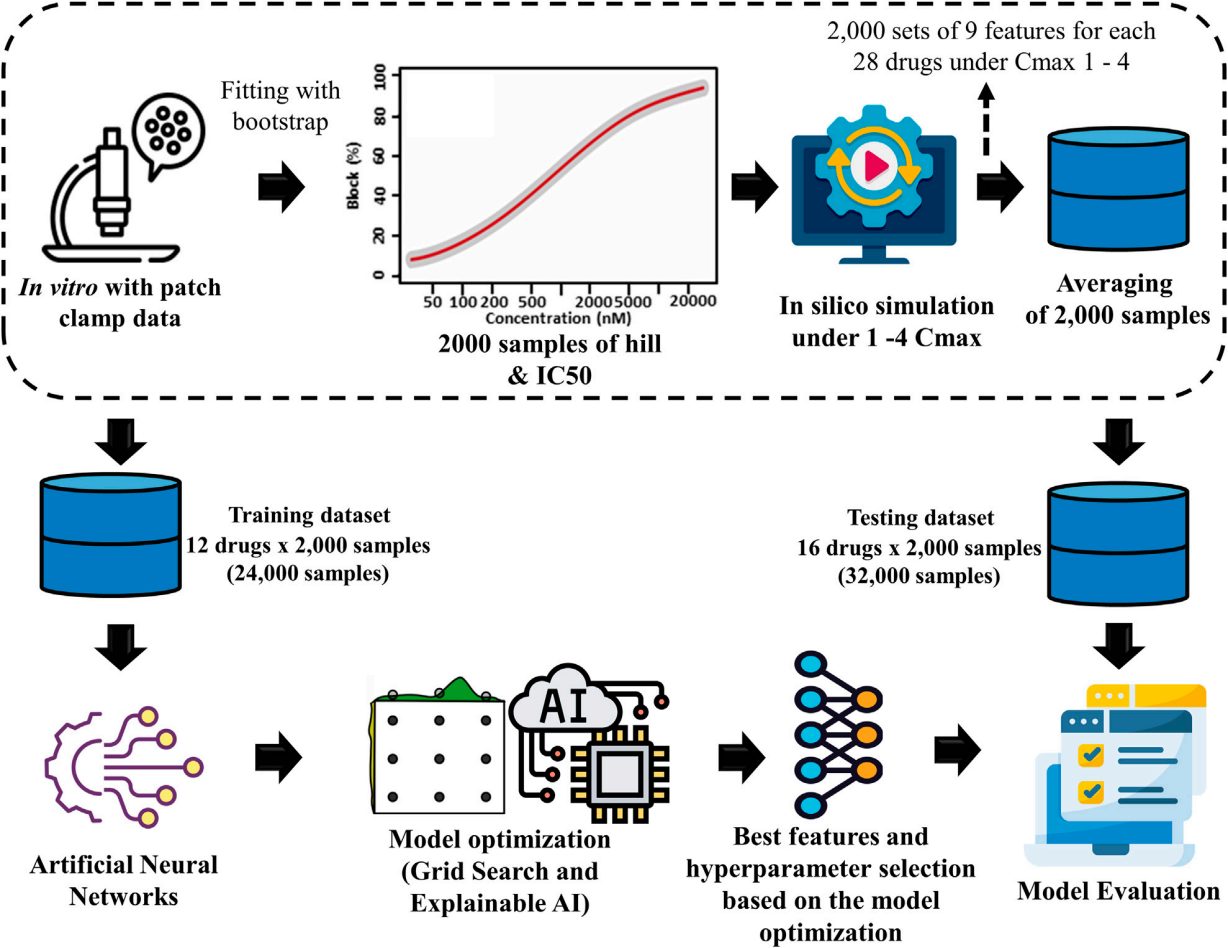
Proposed torsades de pointes (TdP) risk evaluation method. This study utilized 2,000 Hill samples from *in vitro* patch-clamp data provided by Li et al. (2019). These samples were subsequently integrated into the ToR–ORd *in silico* ventricular cell model, given 28 different drugs at 4 maximum concentration levels (C_max_ 1-4), resulting in 2,000 samples for 9 key TdP features. The average sample values were calculated for each sample based on the results obtained at the four distinct drug concentration levels. Then, the dataset was segmented into 2 parts: a training dataset consisting of 24,000 samples (12 drugs × 2,000 samples) used in the artificial neural network (ANN) classifier model and a testing dataset consisting of 32,000 samples (16 drugs × 2,000 samples) for evaluation through 10,000 testing iterations.

Figure 1 illustrates the comprehensive methodology, which includes several stages. The first step involved generating drug samples using the Markov chain Monte Carlo (MCMC) method. Then, biomarkers were produced from *in silico* simulation. The biomarkers acted as features for the ANN to predict the TdP risk of drugs. Finally, the ANN model was optimized by GS hyperparameter optimization and feature selection using XAI.

### 2.1 *In vitro* experimental dataset and MCMC simulation

This study evaluated the drug toxicity risk using 28 drugs from the *in vitro* dataset, as shown in Table 1 (Li et al., 2019). This dataset applied the inhibition levels of four ion channels (INaL, INa, ICaL, and IKr) for training and testing datasets. For the dose–response analysis, the data that are uploaded by the CiPA group are accessible at https://github.com/FDA/CiPA/tree/Model-Validation-2018/Hill_Fitting/data. We also applied the same Hill fitting method used by Li et al. (2019) to ensure the consistency and validity of our analysis. This technique produced 2,000 Hill curves illustrating the relationship between ion channel blockade and drug concentration described by drug concentrations leading to the half-maximal inhibitory concentration (IC_50_) values of the ionic channels and the slope of the Hill curve (h or Hill coefficient). Finally, the 2,000 IC_50_ values and h samples for each drug were used as inputs for *in silico* simulations.

**TABLE 1:** Drug information based on the Comprehensive *in vitro* Proarrhythmia Assay (CiPA) guideline (Li et al., 2019).

Proarrhythmic risk level	Train drugs		Test drugs	
	C_max_ (nM)	Drug name	C_max_ (nM)	Drug name
High	33	Bepridil	70	Azimilide
	2	Dofetilide	742	Disopyramide
	1,439	Sotalol	100	Ibutilide
	3,237	Quinidine	255.4	Vandetanib
Intermediate	2.6	Cisapride	0.26	Astemizole
	38	Chlorpromazine	1,206	Clarithromycin
	139	Ondansetron	71	Clozapine
	4	Terfenadine	19	Domperidone
			6.33	Droperidol
			0.431	Pimozide
			1.81	Risperidone
Low	122	Diltiazem	0.45	Loratadine
	4,129	Mexiletine	1,800	Metoprolol
	1,948.2	Ranolazine	7.7	Nifedipine
	81	Verapamil	3.02	Nitrendipine
			21	Tamoxifen

### 2.2 Preprocessing and the ToR–ORd *in silico* ventricular cell model

This study used the *in silico* ToR–ORd ventricular cell model to simulate cardiomyocyte electrophysiology. The *in silico* simulator was developed using the C++ programming language as the underlying code supported by libraries such as CVode to solve differential equations. The *in silico* simulator code is given in Supplementary Material S1. The effects of drugs on myocardial cells were quantified using Eq. 1, which includes the IC_50_ parameter that represents the concentration at which the half-maximal effect is caused, the Hill coefficient determining the dose–response curve (H), and drug concentration (D). We used Eq. 1 to calculate the inhibitory factor, which indicates how the drug impacts the *in silico* ToR–ORd ventricular cell model. Eq. 2 describes the maximum conductance g of ion channel i under the drug effect. Note that $g_{\text{control},i}$ represents the maximum conductance of the ion channel without the drug effect.

$$\text{Inhibition factor}=\frac{1}{1+{\left(\frac{\text{IC}50}{\left|D\right|}\right)}^{h}}$$ (1)

$$g_{i}=g_{\text{control},i}\left(1-\text{inhibition factor}\right)$$ (2)

In this study, we implemented four different concentrations for each type of drug for *in silico* simulations. Each drug concentration produced a total of 2,000 samples for each TdP metric, and we utilized a cumulative total of 8,000 samples (2,000 samples for each of the 4 drug concentrations) as the input. One beat with the highest $\frac{\text{dVm}}{{\text{dt}}_{\max\;\_\text{repol}}}$ among the last 250 beats was selected for generating TdP metrics (Chang et al., 2017). Through these simulations, the electrophysiological response of human ventricular myocytes was influenced by the drug effects with 1,000 pacing cycles with a duration of 2,000 milliseconds (ms) for each pacing.

We utilize a set of metrics to analyze the impact of drugs on human ventricular cardiac cells. These metrics encompass the concept of net charge, with qNet reflecting the total charge alteration during the simulation, along with qInward, which measures the inward charge during the simulation, as expressed by Eqs 3, 4:

$$\text{qNet}=\int_{0}^{\text{BCL}}\left(I_{\text{Kr}}+I_{\text{CaL}}+I_{\text{NaL}}+I_{\text{to}}+I_{\text{Ks}}+I_{K1}\right)\text{dt}$$ (3)

$$\text{qInward}=\frac{1}{2}\left(\frac{\int_{0}^{\text{BCL}}I_{\text{NaL},\text{drug}}\text{dt}}{\int_{0}^{\text{BCL}}I_{\text{NaL},\text{control}}\text{dt}}+\frac{\int_{0}^{\text{BCL}}I_{\text{CaL},\text{drug}}\text{dt}}{\int_{0}^{\text{BCL}}I_{\text{CaL},\text{control}}\text{dt}}\right)$$ (4)

Furthermore, we consider calcium-related metrics, such as ${\text{CaD}}_{90}$ , indicating when calcium reaches 90% repolarization during the cycle. ${\text{CaD}}_{50}$ measures the time when calcium reaches 50% repolarization during the cycle, and ${\text{Ca}}_{\text{resting}}$ is used for assessing the calcium level during the relaxation phase. We also consider metrics related to APs, including ${\frac{\text{dVm}}{\text{dt}}}_{\max}$ , which represents the maximum rate of change in the membrane potential during the depolarization phase of the AP cycle. ${\text{APD}}_{90}$ indicates the duration of the AP at 90% repolarization, while ${\text{APD}}_{50}$ measures the duration of the AP at 50% repolarization. Lastly, the Vm_resting_ metric measures the AP duration during the resting phase.

Using drug samples generated using the MCMC process, we acquired 56,000 samples (2,000 samples × 28 types of drugs). From the 28 drug types released by the CiPA group, as given in Table 1, we selected 12 drugs as the training dataset. Therefore, we utilized 24,000 samples (2,000 samples × 12 drugs). Each sample consists of nine TdP metrics, and it will be used as the input data in the ANN simulation that will be optimized by GS. We implemented all machine learning and model evaluation codes within a Jupyter Notebook using Python programming. The simulation code of our proposed model is given in Supplementary Material S1.

### 2.3 The proposed ANN optimization by grid search

This study divided 56,000 samples generated from *in silico* simulations into 2 sets to develop and evaluate an ANN model. The first set, comprising 24,000 samples with 2,000 samples for 12 different drugs each, was designated as the training dataset. To ensure the accuracy and robustness of the ANN model, we employed a 10-fold cross-validation method during the training process. In each cross-validation iteration, 21,600 samples were used for training and 2,400 samples for validation, which enabled a comprehensive assessment of the model performance across different subsets of training data. The remaining 32,000 samples, corresponding to 2,000 samples for 16 other drugs each, formed the testing dataset. This ensured a rigorous evaluation of the ANN’s predictive capabilities on unseen data. Accordingly, the model is both generalizable and effective in simulating drug effects based on the diverse profiles of the drugs.

We implemented the GS hyperparameter optimization approach in the ANN classifier. This approach aims to identify the optimal parameter configuration capable of achieving optimal performance for a machine learning architecture. A GS systematically tests all possible combinations of the provided parameters. It evaluates the model performance for each hyperparameter combination, and this process requires training and evaluating the model using various parameter combinations. The results of these evaluations then serve as the basis for selecting the parameter combination that yields the best performance.

We propose various hyperparameters, including batch size (32 and 64), optimization (RMSprop and Adam), the number of neurons in the hidden layers (5, 6, and 7), learning rates (0.1, 0.001, and 0.01), and alpha values (0.1, 0.01, 0.001, 0.2, 0.02, and 0.002). Here, regularization techniques lasso (L1) and ridge (L2) (0.01) were utilized in the first hidden layer to control complexity and mitigate overfitting. In each iteration, GS combined each existing parameter value (e.g., the combination of a batch size of 32 with RMSprop optimization, 5 neurons, a learning rate of 0.1, and an alpha value of 0.001). The ANN model was updated and evaluated using multiple parameter combinations. This process was repeated for all possible combinations, resulting in parameter combinations that provide the best performance on the dataset. The results from this ANN model were employed for classifying the TdP risk of drugs, which are high risk, intermediate risk, and lowrisk. The classification process utilized the output of the softmax function to generate risk probabilities.

### 2.4 Explainable AI using the ANN classifier

In XAI, SHAP values are important because they offer a consistent and theoretical way to measure how each feature contributes to prediction accuracy. Applying SHAP values to complex models such as an ANN is recommended since complex models cannot be explained easily using intrinsic explanations (Zhang et al., 2020; Thisovithan et al., 2023). A SHAP value analysis provides an insight into the contribution of each feature member to the collation value of a model. The results of SHAP values can be considered in light of the contribution made by each feature that has a positive or negative impact on the target output. The SHAP value for a specific data point is described in Eqs 5, 6, where *X* represents the feature value vector (which needs to be explained), *S* indicates the input feature subset, and the Shapley value can be obtained through the value function $f_{x}\left(S\right)=E\;\left[f\left(x\right)\left.\;\right|x_{s}\right]$ .

$$\emptyset_{i}=\sum S\underline{\subseteq }\left\{1,\ldots ,p\right\}\left\{i\right\}\frac{\left|S\right|!\left(p-\left|S\right|-1\right)!}{p!}$$ (5)

$$\left[f_{x}\left(S\cup \left\{i\right\}\right)-f_{x}\;\left(S\right)\right]$$ (6)

### 2.5 Model evaluation

**FIGURE 2:**
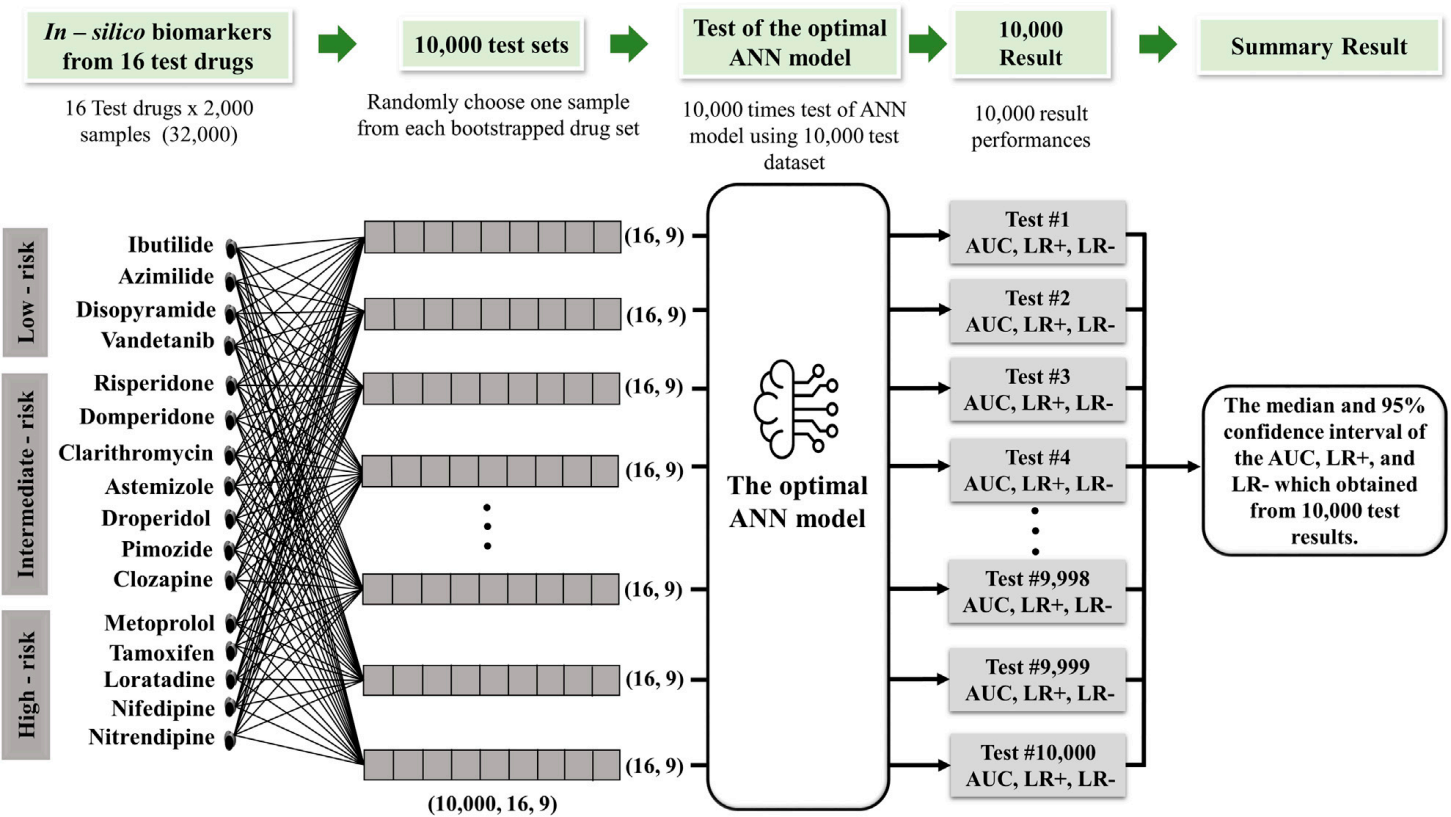
Schematic diagram of the 10,000 test algorithms based on the the study by Jeong et al. (2022).

In order to assess the effectiveness of a classification model, we implemented 10,000 test protocols as outlined in the CiPA initiative by Li et al. (2019), as shown in Figure 2. The dataset encompassed 32,000 samples, comprising 16 drug categories containing 2,000 samples. We generated 10,000 random sample subsets from this dataset covering all 16 drug categories. The model was evaluated by generating 10,000 receiver operating characteristic (ROC) curves for each risk category: high, intermediate, and low. The model performance was then statistically assessed by calculating the area under the ROC curve (AUC), positive and negative likelihood ratios, and the average classification error to measure the model accuracy.

The positive and negative likelihood ratios were determined using Eqs 6, 7. Model sensitivity and specificity are two essential components of these ratios. Sensitivity and specificity were calculated based on Eqs 8, 9, where a true positive (TP) defines the number of positive cases accurately identified by the model; true negative (TN) refers to accurately predicted negative cases; false positive (FP) indicates negative cases erroneously predicted as positive; and false negative (FN) represents positive cases mistakenly classified as negative.

$$\text{Likelihood ratio positive }\left(\text{LR}+\right)=\frac{Sensitivity}{1-specificity}$$ (7)

$$\text{Likelihood ratio negative }\left(\text{LR}-\right)=\frac{1-sensitivity}{specificity}$$ (8)

$$Sensitivity=TP/\left(TP+FN\right)$$ (9)

$$Specificity=TN/\left(TN+FP\right)$$ (10)

Based on the ROC curve, the AUC indicates the ability of the model to distinguish between positive and negative classes by measuring the relationship between the true positive rate (TPR) and false positive rate (FPR) using Eqs 10, 11. The TPR measures how sensitive the model is to identifying positive cases, whereas the FPR measures how often the model generates false positive predictions in negative situations.

$$\text{False positive rate }\left(\text{FPR}\right)=\frac{FP}{FP+TN}$$ (11)

$$\text{True Positive rate }\left(\text{TPR}\right)=\frac{TP}{TP+FN}$$ (12)

The model performance was evaluated using median values and 95% confidence intervals, calculated from the 2.5th to 97.5th percentiles of the test outcomes. Meanwhile, the 95% confidence interval for the average error was determined using the formula mean ±1.96 * SD/√N, where SD is the standard deviation of the error and N indicates the total number of samples (16 drug tests multiplied by 2,000 samples for validation).

## 3 Results

**FIGURE 3:**
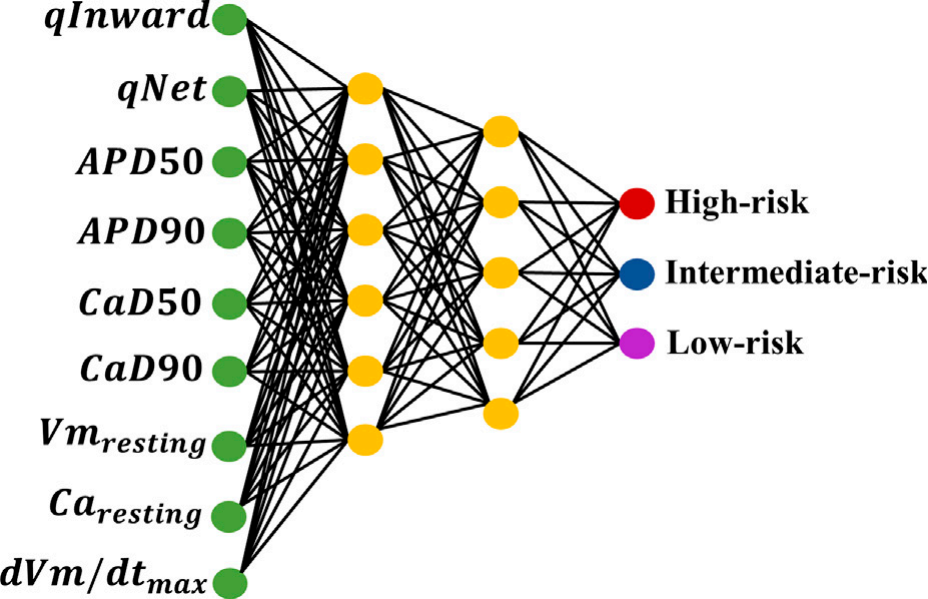
ANN architecture optimized by grid search (GS) hyperparameter optimization.

This study classified TdP risk with various drugs inducing TdP metrics within the ToR–ORd ventricular *in silico* cell model. We implemented 10-fold cross-validation using the GS method to train 12 drugs using the ANN classifier. We employed the GS method for automatic hyperparameter tuning to select the optimal parameters for the ANN classifier. The detailed structure of the ANN model, based on the GS simulation results, is shown in Figure 3. According to the GS method, six neurons were used in the first layer and five neurons in the second layer, with an alpha value of 0.1 applied to the activation function for the leaky ReLU for both layers. The model was optimized using Adam as the optimizer and trained for 200 epochs with a learning rate of 0.001. The training process was conducted using a batch size of 32. The outcomes obtained from this ANN model classified the TdP drug risks into three risk categories: high, intermediate, and low. This classification was achieved through the output layer utilizing the softmax function to generate risk probabilities. The model performance was evaluated through a 10-fold cross-validation procedure to validate the robustness of the model. Subsequently, the best-performing model was tested using 16 test datasets and subjected to 10,000 iterations of testing.

**FIGURE 4:**
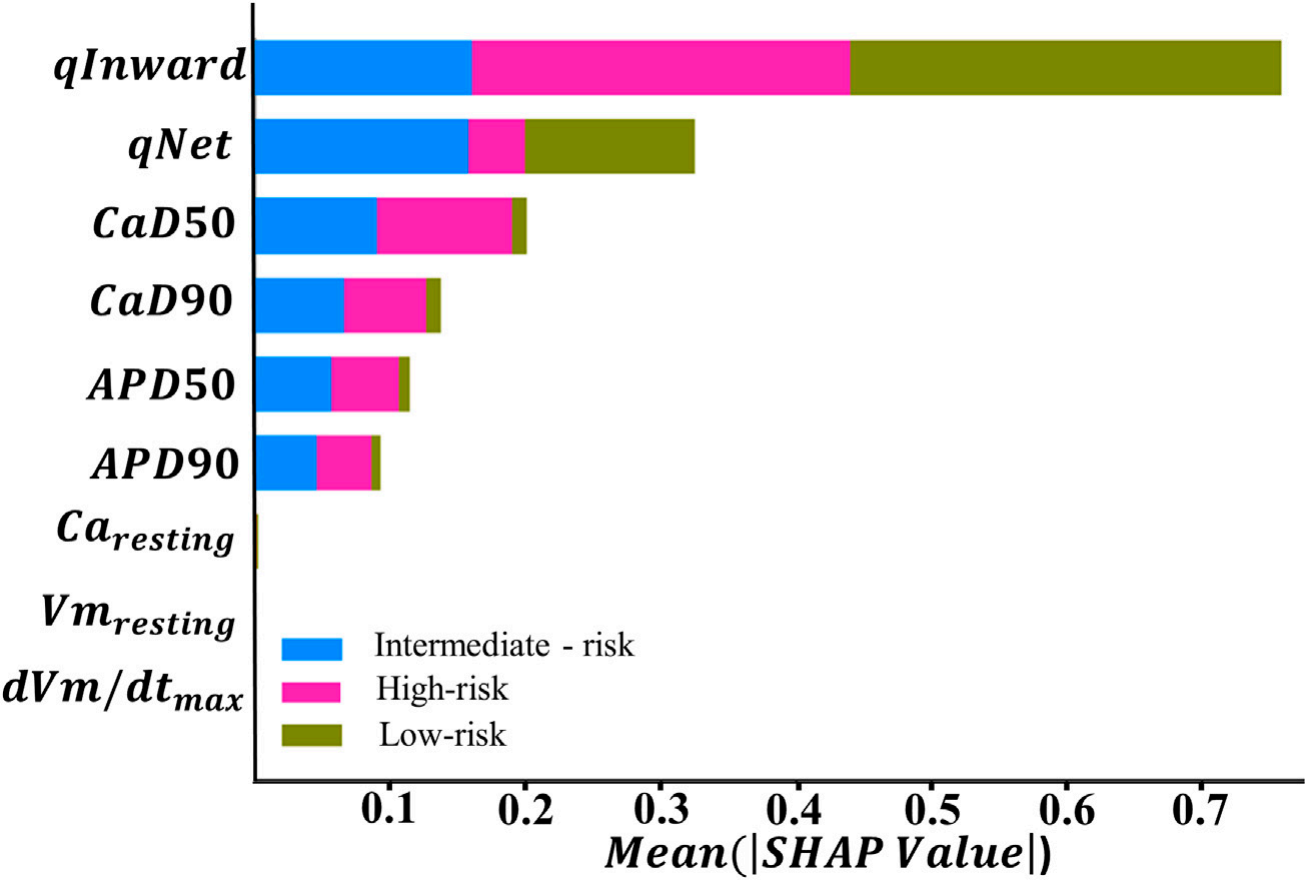
Feature importance ranking based on the SHapley Additive exPlanation (SHAP) values from explainable artificial intelligence (XAI) with the ANN, which was optimized by GS hyperparameter optimization.

Furthermore, we analyzed nine features $({\frac{dV}{\text{dt}}}_{\max}$ ; ${\text{Vm}}_{\text{resting}}$ ; ${\text{APD}}_{90}$ ; ${\text{APD}}_{50}$ ; ${\text{Ca}}_{\text{resting}}$ ; ${\text{CaD}}_{90}$ ; ${\text{CaD}}_{50}$ ; qNet; and qInward) from the training dataset to assess feature contributions to each class. By using the ANN classifier, we performed feature importance analysis using the SHAP method (Zhang et al., 2020; Yunendah et al., 2023) to evaluate feature contributions to the ANN classifier. Figure 4 shows SHAP values obtained from the trained ANN model to classify TdP risk. SHAP values provide an interpretative method for understanding the influence of each feature on the predictions generated by the model.

In the context of TdP risk induced by various drugs, Figure 4 shows that certain features exhibit higher SHAP values, indicating a dominant role in influencing the model decisions. Conversely, features with lower SHAP values contribute minimally to the model decisions in classifying TdP risk. Based on the SHAP results given in Figure 4, qInward demonstrates the most dominant contribution to both high- and low-risk classes, followed by ${\text{CaD}}_{50}$ , ${\text{CaD}}_{90}$ , ${\text{APD}}_{50}$ , ${\text{APD}}_{90}$ , and qNet. Meanwhile, ${\text{Ca}}_{\text{resting}}$ , ${\text{Vm}}_{\text{resting}}$ , and ${\frac{dV}{\text{dt}}}_{\max}$ show minimal contributions to these classification. In order to analyze the performance of each feature, we conducted individual feature analyses within the ordinal logistic regression model. As shown in Table 2, qNet, APD_50_, qInward, and CaD_50_ achieved high performances for high risk.

**TABLE 2:** Single feature performance evaluation.

	AUC			LR+			LR−			Mean classification error
	High	Inter	Low	High	Inter	Low	High	Inter	Low	
qNet	0.8750 (0.8125, 0.9375)	0.6984 (0.6508, 0.7619)	0.8364 (0.7818, 0.8727)	500,000.9866 (499999.9937, 500001.9775)	1.5238 (1.2857, 2.5000)	2.9333 (2.2000, 8.7999)	0.5000 (0.5000, 0.5000)	0.6857 (0.2857, 0.8571)	0.2750 (0.2200, 0.5500)	0.3731 (0.3677, 0.3785)
APD_50_	0.8750 (0.8125–0.9583)	0.6984 (0.6349–0.7460)	0.8000 (0.7455–0.8545)	500,000.9954 (500,000.0068, 500,001.9821)	1.2857 (0.7857, 1.9286)	1.7600 (1.3200, 2.7500)	0.5000 (0.5000, 0.5000)	0.8571 (0.6531, 1.1224)	0.4400 (0.2750, 0.7333)	0.4931 (0.4872, 0.4991)
qInward	0.8542 (0.7708, 0.9167)	0.6508 (0.5556, 0.7143)	0.8000 (0.7455, 0.8727)	5.9999 (3.0000, 500001.5687)	1.2000 (0.9524, 1.5000)	400000.2076 (2.2000, 400001.6751)	0.5455 (0.2727, 0.6000)	0.5000 (0.0000, 1.1429)	0.6000 (0.6000, 0.8800)	0.4022 (0.3968, 0.4076)
APD_90_	0.7455 (0.6905–0.8000)	0.6667 (0.6032–0.7143)	0.7455 (0.6905–0.8000)	500,000.9979 (500,000.0056, 500,001.9745)	1.0714 (0.7857, 1.7143)	1.3200 (1.1000, 2.2000)	0.5000 (0.5000, 0.5000)	0.9524 (0.7143, 1.1224)	0.7333 (0.3667, 0.8800)	0.5344 (0.5283, 0.5405)
CaD_50_	0.8646 (0.7917, 0.8958)	0.5238 (0.4683, 0.5794)	0.7091 (0.6455, 0.7273)	3.0000 (3.0000, 5.9999)	0.0000 (0.0000, 1.0000)	0.9429 (0.8250, 1.2571)	0.6000 (0.5455, 0.6000)	1.2000 (1.0000, 1.3333)	1.1000 (0.5500, 1.4667)	0.8194 (0.8122, 0.8265)
CaD_90_	0.2708 (0.2292, 0.3333)	0.6508 (0.5635, 0.7222)	0.5273 (0.4909, 0.6000)	0.7500 (0.6667, 0.7500)	1.0000 (1.0000, 1.0000)	2.2000 (1.4667, 2.2000)	1.5000 (1.5000, 2.0000)	1.0000 (1.0000, 1.0000)	0.5500 (0.5500, 0.8250)	0.9431 (0.9349, 0.9513)
Ca_Diastole	0.4062 (0.3229, 0.4896)	0.5952 (0.5079, 0.6667)	0.5000 (0.4636, 0.5455)	0.0000 (0.0000, 1.0000)	1.1429 (0.8571, 1.5238)	2.2000 (1.4667, 2.2000)	1.3333 (1.0000, 1.5000)	0.8571 (0.6857, 1.1429)	0.7333 (0.7333, 0.8250)	0.7743 (0.7665, 0.7821)
dVm_dt_Max	0.6364 (0.4364, 0.7273)	0.7143 (0.5714, 0.8571)	0.5000 (0.3542, 0.6042)	0.0000 (0.0000, 3.0000)	1.5238 (0.8571, 4.0000)	2.2000 (1.1000, 4.4000)	1.2000 (0.8182, 1.5000)	0.5714 (0.2143, 1.1429)	0.5500 (0.2444, 0.9429)	0.6720 (0.6639, 0.6801)
Vm_Resting	0.1458 (0.0833, 0.2292)	0.5079 (0.4444, 0.5873)	0.1818 (0.1273, 0.2364)	0.5000 (0.5000, 0.5000)	0.0000 (0.0000, 0.0000)	0.0000 (0.0000, 1.0000)	1.1000 (1.0000, 1.1000)	1.1250 (1.1250, 1.2857)	500,001.0122 (500,000.0253, 500,001.9961)	1.2286 (1.2215, 1.2358)

Furthermore, we examined the feature importance rankings provided by the SHAP values for the ANN classifier using nine features. Subsequently, this analytical process was expanded with additional experiments involving the reduction of features that did not show significant contributions based on the SHAP value analysis. The top feature groups, analyzed based on their contributions to the model, comprised five, six, seven, and eight top features. Among them, the group with five features consisted of qInward, qNet, ${\text{CaD}}_{50}$ , ${\text{CaD}}_{90}$ , and ${\text{APD}}_{50}$ . Then, the top six feature groups were qInward, qNet, ${\text{CaD}}_{50}$ , ${\text{CaD}}_{90}$ , ${\text{APD}}_{50}$ , and ${\text{APD}}_{90}$ . The group of the top seven features consisted of qInward, qNet, ${\text{CaD}}_{50}$ , ${\text{CaD}}_{90}$ , ${\text{APD}}_{50}$ , ${\text{APD}}_{90}$ , and Ca_resting_. In addition, the group of the top eight features encompassed qInward, qNet, ${\text{CaD}}_{50}$ , ${\text{CaD}}_{90}$ , ${\text{APD}}_{50}$ , ${\text{APD}}_{90}$ , Ca_resting_, and ${\text{Vm}}_{\text{resting}}$ . This analysis provided an insight into the importance of each feature in the context of TdP risk induced by various drugs.

Table 3 shows the evaluation of the performance of a predictive model in simulations. In the configuration with 5 features, the model demonstrated an effective ability with an AUC of 0.930 for high risk and 0.901 for low risk, indicating a good capacity in identifying low-risk events. Meanwhile, the positive likelihood ratio (LR+) was 5.99994, and the negative likelihood ratio (LR−) was 0.583333 for high risk. The average classification error in this configuration was the highest, at 26.5%.

**TABLE 3:** Model performance evaluation after 10,000 iterations. The features were grouped according to SHAP values, as illustrated in Figure 4. The feature with the lowest ranking in the SHAP value analysis was eliminated first. Proarrhythmic risk is classified as high, intermediate, and low. The Area Under the Curve (AUC), Likelihood Ratio Positive (LR+), Likelihood Ratio Negative (LR−), and mean classification error were utilized for model evaluation.

Performance evaluation		Number of features					Li et al. (2019)
		5	6	7	8	9	
AUC	High	0.930 (0.850–0.981)	0.937 (0.8125–1.0)	0.961 (0.901–1.0)	0.943 (0.791, 0.958)	0.979 (0.895, 1.0)	0.89 (0.84–0.95)
	Inter	0.750 (0.666–0.834)	0.777 (0.6667–0.8254)	0.780 (0.670–0.831)	0.730 (0.634, 0.793)	0.791 (0.708, 0.875)	-
	Low	0.901 (0.818–0.916)	0.900 (0.810–0.961)	0.917 (0.833–1.0)	0.927 (0.854, 0.981)	0.937 (0.854, 0.958)	1 (0.92–1)
LR+	High	5.99994 (5.9999400005–5.9999400008)	6.599 (3.299–600,001.206)	400,000.3 (2.1999–400,001.7)	400,000.9 (200,000.7, 400,001.7)	400,000.3 (2.199, 400,001.6)	4.5 (2.5–5)
	Inter	1.571427 (1.428570–1.714284)	1.71 (1.428–1.857)	2.399 (2.199–2.599)	2.4 (2.2, 2.6)	2.6 (2.4, 2.6)	-
	Low	3.299987 (1.649998–6.599938)	5.99994 (2.999988–5.999940)	250,001 (250,000.5–500,001.4)	5.999 (2.999, 500002.3)	250,001 (250,000.4, 250001.5)	12 (4.5–1e+06)
LR−	High	0.583333 (0.312500–0.5833338)	0.312 (0.0–0.5909)	0.318 (1.333331588936186e-06–0.6111)	0.812 (0.5833, 0.8125)	1.444E-06 (1.363e-06, 0.333)	0.11 (1.2e-06–0.23)
	Inter	0.52381 (0.285715–0.571429)	0.523 (0.2653–0.7714)	0.643 (0.464, 0.714)	0.357 (0.314, 0.642)	0.642 (0.464, 0.816)	-
	Low	0.533334 (0.520000–0.550000)	0.533 (0.52–0.55)	0.533 (0.520, 0.550)	0.520 (0.52, 0.5333)	0.520000624 (0.520000624, 0.533)	1.1e-06 (1e-06–0.3)
Mean classification error		0.265 (0.142–0.276)	0.239 (0.136–0.304)	0.217 (0.136–0.304)	0.225 (0.213–0.315)	0.214 (0.145–0.279)	0.194 (0.1973–0.1975)

With the addition of 6 features, an increase was observed in the AUC to 0.937 for high risk and 0.777 for intermediate risk, along with a decrease in the average classification error to 23.9%. This increase indicates that adding features provides additional benefits to the discriminative performance of the model. LR+ increased to 6.599, indicating improved predictive performance of the model for positive classification, while LR− decreased to 0.523, indicating improvement in the model ability to exclude high risk in negative results.

In the configuration using 7 features, a significant increase was observed in the AUC for high risk to 0.961, indicating excellent model performance. Meanwhile, the AUC for intermediate risk also increased to 0.780 and low risk increased to 0.917. LR+ for high risk significantly increased to 400,000.3, indicating substantial improvement in predicting high-risk positives. Although LR− experienced a slight increase to 0.533, the average classification error decreased to 21.7%, reflecting increased predictive accuracy.

The configuration with eight features showed a slight decrease in the AUC for high and intermediate risks. However, the AUC for low risk increased slightly to 0.927. LR+ remained high at 400,000.9, maintaining substantial predictive strength for positive classification, while LR− showed a remarkable decrease to 0.312, indicating improved performance in excluding high risk in negative results. The average classification error remained stable at 22.5%, indicating consistent predictive accuracy with adding the eighth feature.

**FIGURE 5:**
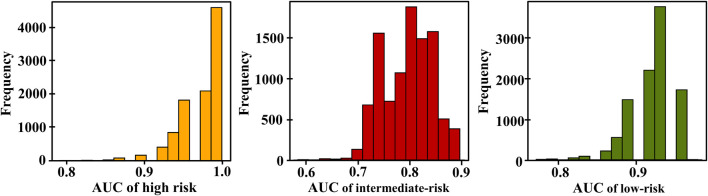
Distribution of area under the curves (AUCs) based on the TdP risk using 9 features of 16 test drugs; yellow, red, and green are the AUC distribution for high, intermediate, and low risks, respectively.

In our experiment, we observed that using nine features shows optimal classification performance. Table 3 shows that the model achieved an AUC of 0.979 for high risk, with a 95% confidence interval of 0.895–1.0. For intermediate risk, the obtained AUC was 0.791, with a 95% confidence interval between 0.708 and 0.875. Regarding low risk, the AUC value was 0.937, with a 95% confidence interval between 0.854 and 0.958. The distribution of the AUC utilizing nine features in the ANN is shown in Figure 5. LR+ for high risk remained high at 400,000.3, indicating strong predictive performance for high-risk positive classification. LR− remained low at 0.533, suggesting that the model effectively excludes high risk in negative cases. The average classification error decreased to 21.4%, indicating that the further addition of features correlates with the highest observed accuracy among all feature sets.

Therefore, this experiment shows that removing specific features does not significantly affect the performance of the classification model. Based on this finding, the eliminated features may not play a significant role in the decision-making process by the ANN classifier. Table 3 illustrates the performance achieved by the ANN classifier using the complete set of nine features and the reduced feature set. The ANN classifier model performs better with the complete set of nine features than with the reduced feature group.

## 4 Discussion

In this study, our primary objective was to evaluate TdP risk prediction by employing the ToR–ORd model. We used 28 drug datasets through the CiPA, as shown in Table 1. This study presents the potential applicability of nine TdP metrics ( ${\frac{dV}{\text{dt}}}_{\max}$ ; ${\text{Vm}}_{\text{resting}}$ ; ${\text{APD}}_{90}$ ; ${\text{APD}}_{50}$ ; ${\text{Ca}}_{\text{resting}}$ ; ${\text{CaD}}_{90}$ ; ${\text{CaD}}_{50}$ ; qNet; and qInward) to predict TdP risks. We employed these metrics with an ANN classifier. Furthermore, we fine-tuned them through the hyperparameter optimization process known as GS to categorize TdP risks effectively.

Previous research proposed an algorithm for assessing the risk of TdP classification based on ion channel measurements that are affected by the drugs and obtained through *in vitro* experiments (Crumb et al., 2016; Li et al., 2017; Li et al., 2019; Parikh et al., 2017). The CiPA research group recommends the calculation of qNet, which is the sum of ion charges passing through six ion channels (INaL, ICaL, IKr, IKs, and Ito) (Li et al., 2017). A previous study utilized qNet as a single *in silico* biomarker under the IKr-dyn ORd cell model to classify TdP risks (high and low risks) using ordinal logistic regression and successfully obtained superior performances, whereas the performance was decreased when utilized without hERG (static) (Li et al., 2019). However, involving the dynamic hERG channel model to analyze the *in vitro* experimental data often exhibits significant challenges. As shown by the work of the CiPA group (Li et al., 2019), the authors provided a “hybrid” dataset by combining the dynamic hERG parameters obtained from “manual” patch-clamp experiments using a high-throughput automated patch-clamp system (HTS) that did not capture dynamic hERG model parameters, indicating possible challenging conditions to replicate the dynamic hERG experiments.

A previous study successfully obtained higher performances for classifying TdP risks by implementing several features under the CiPAORdv1.0 ventricular *in silico* cell model without incorporating the dynamic hERG into the machine learning process (Yoo et al., 2021). The use of AP morphology, calcium transient morphology, and charge features allowed for a more comprehensive evaluation of drug cardiotoxicity and enhanced the accuracy of drug toxicity assessments, consistent with the findings obtained by Lancaster and Sobie (2016). Nevertheless, the significance of each feature in influencing the TdP risk classification performance of the machine learning model was not elaborated. Identifying the most influential feature for classifying between high, intermediate, and low risks is essential. Feature analysis can assist the development of a more simplified and efficient model by focusing solely on the most relevant features.

A previous study utilized the ToR–ORd model to evaluate the TdP metrics derived from single APs, intracellular calcium dynamics, and ionic charge obtained from the effects of drugs on the ToR–ORd ventricular cell model to classify TdP risks using ordinal logistic regression (Jeong et al., 2022). However, using single TdP metrics from the *in silico* models for classifying the TdP risks is challenging to handle complex or non-linear relationships between independent and dependent variables. The dependent variable is influenced by various factors that interact simultaneously, necessitating the incorporation of multiple features to gain a more precise understanding of these intricate relationships. Thus, using several TdP metrics could enhance the model capacity for addressing complex issues, as done by Yoo et al. (2021).

In this study, we evaluated the performance of machine learning classification in predicting TdP risk groups: high, intermediate, and low. Subsequently, we conducted additional analysis by reducing features based on the order of feature importance generated from the SHAP value analysis. Figure 4 shows qInward as the top-ranked feature, contributing significantly to the performance of the ANN classifier model. qInward has the most substantial effect on classification, particularly in the high-risk category due to alterations in the electronic charge within the INaL and ICaL ion channels of the heart. These changes significantly impact cardiac ion currents and serve as primary triggers for TdP occurrence. In high-risk drugs such as quinidine, bepridil, sotalol, and dofetilide, qInward demonstrates rapid and concentration-dependent increases. Higher drug doses correspond to faster and larger increments in qInward values (Li et al., 2017). Consistent with our findings, prior research has also highlighted the dominant role of qInward variability in convolutional neural network (CNN) classifiers for TdP risk classification (Jeong et al., 2022).

A significant disparity in performance is observed between the qNet model proposed by Li et al. (2019) and the qNet model using ToR–ORd *in silico* model. This discrepancy can be ascribed to striking structural differences in the hERG current between the ORd and ToR–ORd *in silico* models. Specifically, the ORd *in silico* model integrates additional components to depict pharmacodynamic effects, while such components are absent in the ToR–ORd *in silico* model (Li et al., 2017; Tomek et al., 2019). Tomek et al. (2019) reported that the ToR–ORd *in silico* model remains relevant and effective in simulating human cardiac electrophysiological responses. This model generates data that are well aligned with experimental data, indicating good concordance even without specific optimization. The ToR–ORd *in silico* model successfully predicted responses to various ion channel blockades, including IKr (E-4031), IKs (HMR-1556), multichannel mexiletine blockade, and ICaL (nisoldipine), which is consistent with experimental observations.

Subsequently, the ranking of SHAP values for each feature in the classification contribution of each class is followed by ${\text{CaD}}_{50}$ , ${\text{CaD}}_{90}$ , ${\text{APD}}_{50}$ , ${\text{APD}}_{90}$ , ${\text{Vm}}_{\text{resting}}$ , ${\text{Ca}}_{\text{resting}}$ , and ${\frac{dV}{\text{dt}}}_{\max}$ . Previous studies, including works by Lancaster and Sobie (2016), Li et al. (2019), Yunendah et al. (2023), and Yoo et al. (2021), utilized the significance of these features in classifying TdP risk. However, our analysis shows that ${\text{Ca}}_{\text{resting}},{\text{Vm}}_{\text{resting}}$ , and ${\frac{dV}{\text{dt}}}_{\max}$ provided minimal contributions, as shown in Figure 4. Consequently, ${\text{Ca}}_{\text{resting}},{\text{Vm}}_{\text{resting}}$ , and ${\frac{dV}{\text{dt}}}_{\max}$ have a limited impact on determining TdP risk in the utilized model.

Based on the SHAP analysis results, we grouped the feature set into five groups. Among those groups, utilizing nine features demonstrated superior performance, as shown in Table 3. Our classification performance evaluation was based on diagnostic accuracy metrics (Li et al., 2019), where “excellent” was achieved if AUC ≥0.9, “good” if AUC ≥0.8, and “minimal acceptance” if AUC ≥0.7. Based on Table 3, we observed an intriguing pattern regarding predictive performance as the number of features used varied. Beginning with five features, our model exhibited adequate performance with a sufficiently strong AUC for both low and high risks, even so with a decreased effectiveness for intermediate risk. The sixth feature group improves model performance, indicating classification stability for intermediate and low risks. However, adding the seventh feature did not consistently improve the performance, especially in the high-risk category, suggesting that some additional features may not provide significant predictive information. Transitioning to eight features did not yield significant performance changes, indicating no substantial improvement in accuracy or AUC. Compared to other feature groups, consistently high AUC performance is observed for the high-risk class and low-risk class. Meanwhile, the intermediate risks show performance stability. Feature reduction does not significantly affect TdP risk classification performance; selecting nine features provides superior and comprehensive performance in our predictive model.

Several limitations in this study may require further investigation. First, while a single ToR–ORd model yields relatively high AUC performance, integrating population models remains crucial. Incorporating diverse ventricular APs in *in silico* models can better represent the variability of cellular responses to drugs (Tomek et al., 2019). Therefore, applying these findings to a broader population or different drugs necessitates further research. Additionally, drug calibration is necessary to validate arrhythmia prediction models to ensure alignment with actual drug conditions (Han et al., 2020).

## 5 Conclusion and future works

This study evaluated nine TdP metrics from the ToR–ORd *in silico* ventricular cell model in predicting TdP risk. Initially, we conducted training incorporating the training dataset into an ANN to determine optimum hyperparameters manually through GS. We trained the optimized ANN model with 9 metrics from 12 training data. The optimal ANN model was then tested 10,000 times with 16 drugs that had not been previously used. Next, we analyzed the contribution of nine features, ${\frac{dV}{\text{dt}}}_{\max}$ , ${\text{Vm}}_{\text{resting}}$ , ${\text{APD}}_{90}$ , ${\text{APD}}_{50}$ , ${\text{Ca}}_{\text{resting}}$ , ${\text{CaD}}_{90}$ , ${\text{CaD}}_{50}$ , qNet, and qInward using SHAP value analysis on the ANN model. The SHAP value analysis showed that qInward was the feature that contributed most dominantly to the model. However, after analyzing with single qInward using ordinal logistic regression, qInward provided less satisfactory performance. Furthermore, we conducted an analysis using all nine features and a reduction of features based on the highest and lowest contribution orders from SHAP value results. Based on our findings, reducing features did not significantly impact TdP risk classification performance. The utilization of all nine TdP features with the ANN yielded the highest performance, i.e., an AUC of 0.979 for the high-risk category, 0.791 for the intermediate-risk category, and 0.937 for the low-risk category. These findings contribute to the understanding of utilizing nine features using the ToR–ORd *in silico* cell model. However, further research should consider the development of more complex *in silico* models that encompass a larger population with diverse potential actions of human ventricular and drug calibration to contribute to more significant proarrhythmic risk prediction.

## Data Availability

The original contributions presented in the study are included in the article/Supplementary Material, and further inquiries can be directed to the corresponding author.
