# Supplementary material for: Evaluation of cardiac pro-arrhythmic risks using the artificial neural network with ToR–ORd in silico model output
Source: Front Physiol. 2024 Apr 4;15:1374355. doi: 10.3389/fphys.2024.1374355 (PMC11024991; doi:10.3389/fphys.2024.1374355)
Supplement: Supplementary file 1 [file Table1.pdf]

Supplementary Material

- **Generating ToR – ORd in silico model**

```
/*
   There are a total of 223 entries in the algebraic variable array.
   There are a total of 43 entries in each of the rate and state variable arrays.
   There are a total of 163 entries in the constant variable array.
 */

#include "HEADER_NAME"
#include <cmath>
#include <cstdlib>

/*
 * TIME is time in component environment (millisecond).
 * CONSTANTS[0] is celltype in component environment (dimensionless).
 * CONSTANTS[1] is nao in component extracellular (millimolar).
 * CONSTANTS[2] is cao in component extracellular (millimolar).
 * CONSTANTS[3] is ko in component extracellular (millimolar).
 * CONSTANTS[4] is clo in component extracellular (millimolar).
 * CONSTANTS[5] is R in component physical_constants (joule_per_kilomole_kelvin).
 * CONSTANTS[6] is T in component physical_constants (kelvin).
 * CONSTANTS[7] is F in component physical_constants (coulomb_per_mole).
 * CONSTANTS[8] is zna in component physical_constants (dimensionless).
 * CONSTANTS[9] is zca in component physical_constants (dimensionless).
 * CONSTANTS[10] is zk in component physical_constants (dimensionless).
 * CONSTANTS[11] is zcl in component physical_constants (dimensionless).
 * CONSTANTS[12] is L in component cell_geometry (centimeter).
 * CONSTANTS[13] is rad in component cell_geometry (centimeter).
 * CONSTANTS[112] is vcell in component cell_geometry (microliter).
 * CONSTANTS[130] is Ageo in component cell_geometry (centimeter_squared).
 * CONSTANTS[136] is Acap in component cell_geometry (centimeter_squared).
 * CONSTANTS[142] is vmyo in component cell_geometry (microliter).
 * CONSTANTS[143] is vnsr in component cell_geometry (microliter).
 * CONSTANTS[144] is vjsr in component cell_geometry (microliter).
 * CONSTANTS[145] is vss in component cell_geometry (microliter).
 * STATES[0] is v in component membrane (millivolt).
 * ALGEBRAIC[25] is vffrt in component membrane (coulomb_per_mole).
 * ALGEBRAIC[28] is vfrt in component membrane (dimensionless).
 * ALGEBRAIC[68] is INa in component INa (microA_per_microF).
 * ALGEBRAIC[70] is INaL in component INaL (microA_per_microF).
 * ALGEBRAIC[76] is Ito in component Ito (microA_per_microF).
 * ALGEBRAIC[112] is ICaL in component ICaL (microA_per_microF).
```

* ALGEBRAIC[113] is ICaNa in component ICaL (microA_per_microF).
* ALGEBRAIC[114] is ICaK in component ICaL (microA_per_microF).
* ALGEBRAIC[115] is IKr in component IKr (microA_per_microF).
* ALGEBRAIC[117] is IKs in component IKs (microA_per_microF).
* ALGEBRAIC[121] is IK1 in component IK1 (microA_per_microF).
* ALGEBRAIC[153] is INaCa_i in component INaCa (microA_per_microF).
* ALGEBRAIC[183] is INaCa_ss in component INaCa (microA_per_microF).
* ALGEBRAIC[202] is INaK in component INaK (microA_per_microF).
* ALGEBRAIC[205] is INab in component INab (microA_per_microF).
* ALGEBRAIC[204] is IKb in component IKb (microA_per_microF).
* ALGEBRAIC[209] is IpCa in component IpCa (microA_per_microF).
* ALGEBRAIC[207] is ICab in component ICab (microA_per_microF).
* ALGEBRAIC[214] is IClCa in component ICl (microA_per_microF).
* ALGEBRAIC[216] is IClb in component ICl (microA_per_microF).
* ALGEBRAIC[66] is I_katp in component I_katp (microA_per_microF).
* ALGEBRAIC[11] is Istim in component membrane (microA_per_microF).
* CONSTANTS[14] is i_Stim_Start in component membrane (millisecond).
* CONSTANTS[15] is i_Stim_End in component membrane (millisecond).
* CONSTANTS[16] is i_Stim_Amplitude in component membrane (microA_per_microF).
* CONSTANTS[17] is i_Stim_Period in component membrane (millisecond).
* CONSTANTS[18] is i_Stim_PulseDuration in component membrane (millisecond).
* CONSTANTS[19] is KmCaMK in component CaMK (millimolar).
* CONSTANTS[20] is aCaMK in component CaMK (per_millimolar_per_millisecond).
* CONSTANTS[21] is bCaMK in component CaMK (per_millisecond).
* CONSTANTS[22] is CaMKo in component CaMK (dimensionless).
* CONSTANTS[23] is KmCaM in component CaMK (millimolar).
* ALGEBRAIC[43] is CaMKb in component CaMK (millimolar).
* ALGEBRAIC[49] is CaMKa in component CaMK (millimolar).
* STATES[1] is CaMKt in component CaMK (millimolar).
* STATES[2] is cass in component intracellular_ions (millimolar).
* CONSTANTS[24] is cmdnmax_b in component intracellular_ions (millimolar).
* CONSTANTS[113] is cmdnmax in component intracellular_ions (millimolar).
* CONSTANTS[25] is kmcmdn in component intracellular_ions (millimolar).
* CONSTANTS[26] is trpnmax in component intracellular_ions (millimolar).
* CONSTANTS[27] is kmtrpn in component intracellular_ions (millimolar).
* CONSTANTS[28] is BSRmax in component intracellular_ions (millimolar).
* CONSTANTS[29] is KmBSR in component intracellular_ions (millimolar).
* CONSTANTS[30] is BSLmax in component intracellular_ions (millimolar).
* CONSTANTS[31] is KmBSL in component intracellular_ions (millimolar).
* CONSTANTS[32] is csqnmax in component intracellular_ions (millimolar).
* CONSTANTS[33] is kmcsqn in component intracellular_ions (millimolar).
* STATES[3] is nai in component intracellular_ions (millimolar).
* STATES[4] is nass in component intracellular_ions (millimolar).
* STATES[5] is ki in component intracellular_ions (millimolar).
* STATES[6] is kss in component intracellular_ions (millimolar).
* STATES[7] is cansr in component intracellular_ions (millimolar).

* STATES[8] is cajsr in component intracellular_ions (millimolar).
* STATES[9] is cai in component intracellular_ions (millimolar).
* CONSTANTS[34] is cli in component intracellular_ions (millimolar).
* ALGEBRAIC[91] is ICaL_ss in component ICaL (microA_per_microF).
* ALGEBRAIC[92] is ICaNa_ss in component ICaL (microA_per_microF).
* ALGEBRAIC[95] is ICaK_ss in component ICaL (microA_per_microF).
* ALGEBRAIC[109] is ICaL_i in component ICaL (microA_per_microF).
* ALGEBRAIC[110] is ICaNa_i in component ICaL (microA_per_microF).
* ALGEBRAIC[111] is ICaK_i in component ICaL (microA_per_microF).
* ALGEBRAIC[208] is JdiffNa in component diff (millimolar_per_millisecond).
* ALGEBRAIC[211] is Jdiff in component diff (millimolar_per_millisecond).
* ALGEBRAIC[221] is Jup in component SERCA (millimolar_per_millisecond).
* ALGEBRAIC[206] is JdiffK in component diff (millimolar_per_millisecond).
* ALGEBRAIC[215] is Jrel in component ryr (millimolar_per_millisecond).
* ALGEBRAIC[222] is Jtr in component trans_flux (millimolar_per_millisecond).
* ALGEBRAIC[53] is Bcai in component intracellular_ions (dimensionless).
* ALGEBRAIC[59] is Bcajsr in component intracellular_ions (dimensionless).
* ALGEBRAIC[56] is Bcass in component intracellular_ions (dimensionless).
* CONSTANTS[35] is PKNa in component reversal_potentials (dimensionless).
* ALGEBRAIC[63] is ENa in component reversal_potentials (millivolt).
* ALGEBRAIC[64] is EK in component reversal_potentials (millivolt).
* ALGEBRAIC[65] is EKs in component reversal_potentials (millivolt).
* CONSTANTS[114] is ECl in component reversal_potentials (millivolt).
* CONSTANTS[36] is gkatp in component I_katp (milliS_per_microF).
* CONSTANTS[37] is fkatp in component I_katp (dimensionless).
* CONSTANTS[38] is K_o_n in component I_katp (millimolar).
* CONSTANTS[39] is A_atp in component I_katp (millimolar).
* CONSTANTS[40] is K_atp in component I_katp (millimolar).
* CONSTANTS[115] is akik in component I_katp (dimensionless).
* CONSTANTS[116] is bkik in component I_katp (dimensionless).
* ALGEBRAIC[0] is mss in component INa (dimensionless).
* ALGEBRAIC[13] is tm in component INa (millisecond).
* STATES[10] is m in component INa (dimensionless).
* ALGEBRAIC[1] is hss in component INa (dimensionless).
* ALGEBRAIC[14] is ah in component INa (dimensionless).
* ALGEBRAIC[29] is bh in component INa (dimensionless).
* ALGEBRAIC[37] is th in component INa (millisecond).
* STATES[11] is h in component INa (dimensionless).
* ALGEBRAIC[38] is jss in component INa (dimensionless).
* ALGEBRAIC[15] is aj in component INa (dimensionless).
* ALGEBRAIC[30] is bj in component INa (dimensionless).
* ALGEBRAIC[44] is tj in component INa (millisecond).
* STATES[12] is j in component INa (dimensionless).
* ALGEBRAIC[45] is hssp in component INa (dimensionless).
* STATES[13] is hp in component INa (dimensionless).
* ALGEBRAIC[50] is tjp in component INa (millisecond).

* STATES[14] is jp in component INa (dimensionless).
* ALGEBRAIC[67] is fINap in component INa (dimensionless).
* CONSTANTS[41] is GNa in component INa (milliS_per_microF).
* ALGEBRAIC[2] is mLss in component INaL (dimensionless).
* ALGEBRAIC[16] is tmL in component INaL (millisecond).
* STATES[15] is mL in component INaL (dimensionless).
* CONSTANTS[42] is thL in component INaL (millisecond).
* ALGEBRAIC[3] is hLss in component INaL (dimensionless).
* STATES[16] is hL in component INaL (dimensionless).
* ALGEBRAIC[4] is hLssp in component INaL (dimensionless).
* CONSTANTS[117] is thLp in component INaL (millisecond).
* STATES[17] is hLp in component INaL (dimensionless).
* CONSTANTS[43] is GNaL_b in component INaL (milliS_per_microF).
* CONSTANTS[118] is GNaL in component INaL (milliS_per_microF).
* ALGEBRAIC[69] is fINaLp in component INaL (dimensionless).
* CONSTANTS[44] is Gto_b in component Ito (milliS_per_microF).
* ALGEBRAIC[5] is ass in component Ito (dimensionless).
* ALGEBRAIC[17] is ta in component Ito (millisecond).
* STATES[18] is a in component Ito (dimensionless).
* CONSTANTS[45] is EKshift in component Ito (millivolt).
* ALGEBRAIC[6] is iss in component Ito (dimensionless).
* ALGEBRAIC[18] is delta_epi in component Ito (dimensionless).
* ALGEBRAIC[31] is tiF_b in component Ito (millisecond).
* ALGEBRAIC[39] is tiS_b in component Ito (millisecond).
* ALGEBRAIC[46] is tiF in component Ito (millisecond).
* ALGEBRAIC[51] is tiS in component Ito (millisecond).
* ALGEBRAIC[71] is AiF in component Ito (dimensionless).
* ALGEBRAIC[72] is AiS in component Ito (dimensionless).
* STATES[19] is iF in component Ito (dimensionless).
* STATES[20] is iS in component Ito (dimensionless).
* ALGEBRAIC[73] is i in component Ito (dimensionless).
* ALGEBRAIC[32] is assp in component Ito (dimensionless).
* STATES[21] is ap in component Ito (dimensionless).
* ALGEBRAIC[54] is dti_develop in component Ito (dimensionless).
* ALGEBRAIC[57] is dti_recover in component Ito (dimensionless).
* ALGEBRAIC[60] is tiFp in component Ito (millisecond).
* ALGEBRAIC[61] is tiSp in component Ito (millisecond).
* STATES[22] is iFp in component Ito (dimensionless).
* STATES[23] is iSp in component Ito (dimensionless).
* ALGEBRAIC[74] is ip in component Ito (dimensionless).
* CONSTANTS[119] is Gto in component Ito (milliS_per_microF).
* ALGEBRAIC[75] is fItop in component Ito (dimensionless).
* CONSTANTS[46] is Kmn in component ICaL (millimolar).
* CONSTANTS[47] is k2n in component ICaL (per_millisecond).
* CONSTANTS[48] is PCa_b in component ICaL (dimensionless).
* ALGEBRAIC[7] is dss in component ICaL (dimensionless).

* STATES[24] is d in component ICaL (dimensionless).
* ALGEBRAIC[8] is fss in component ICaL (dimensionless).
* CONSTANTS[49] is Aff in component ICaL (dimensionless).
* CONSTANTS[120] is Afs in component ICaL (dimensionless).
* STATES[25] is ff in component ICaL (dimensionless).
* STATES[26] is fs in component ICaL (dimensionless).
* ALGEBRAIC[77] is f in component ICaL (dimensionless).
* ALGEBRAIC[19] is fcass in component ICaL (dimensionless).
* ALGEBRAIC[9] is jcass in component ICaL (dimensionless).
* ALGEBRAIC[78] is Afcaf in component ICaL (dimensionless).
* ALGEBRAIC[79] is Afcas in component ICaL (dimensionless).
* STATES[27] is fcaf in component ICaL (dimensionless).
* STATES[28] is fcas in component ICaL (dimensionless).
* ALGEBRAIC[80] is fca in component ICaL (dimensionless).
* STATES[29] is jca in component ICaL (dimensionless).
* STATES[30] is ffp in component ICaL (dimensionless).
* ALGEBRAIC[81] is fp in component ICaL (dimensionless).
* STATES[31] is fcafp in component ICaL (dimensionless).
* ALGEBRAIC[82] is fcap in component ICaL (dimensionless).
* ALGEBRAIC[10] is km2n in component ICaL (per_millisecond).
* ALGEBRAIC[20] is anca_ss in component ICaL (dimensionless).
* STATES[32] is nca_ss in component ICaL (dimensionless).
* ALGEBRAIC[21] is anca_i in component ICaL (dimensionless).
* STATES[33] is nca_i in component ICaL (dimensionless).
* ALGEBRAIC[87] is PhiCaL_ss in component ICaL (dimensionless).
* ALGEBRAIC[88] is PhiCaNa_ss in component ICaL (dimensionless).
* ALGEBRAIC[89] is PhiCaK_ss in component ICaL (dimensionless).
* ALGEBRAIC[106] is PhiCaL_i in component ICaL (dimensionless).
* ALGEBRAIC[107] is PhiCaNa_i in component ICaL (dimensionless).
* ALGEBRAIC[108] is PhiCaK_i in component ICaL (dimensionless).
* CONSTANTS[121] is PCa in component ICaL (dimensionless).
* CONSTANTS[131] is PCap in component ICaL (dimensionless).
* CONSTANTS[132] is PCaNa in component ICaL (dimensionless).
* CONSTANTS[133] is PCaK in component ICaL (dimensionless).
* CONSTANTS[137] is PCaNap in component ICaL (dimensionless).
* CONSTANTS[138] is PCaKp in component ICaL (dimensionless).
* ALGEBRAIC[90] is fICaLp in component ICaL (dimensionless).
* ALGEBRAIC[22] is td in component ICaL (millisecond).
* ALGEBRAIC[23] is tff in component ICaL (millisecond).
* ALGEBRAIC[24] is tfs in component ICaL (millisecond).
* ALGEBRAIC[33] is tfcaf in component ICaL (millisecond).
* ALGEBRAIC[34] is tfcas in component ICaL (millisecond).
* CONSTANTS[50] is tjca in component ICaL (millisecond).
* ALGEBRAIC[35] is tffp in component ICaL (millisecond).
* ALGEBRAIC[40] is tfcafp in component ICaL (millisecond).
* CONSTANTS[51] is vShift in component ICaL (millivolt).

* CONSTANTS[52] is offset in component ICaL (millisecond).
* CONSTANTS[122] is Io in component ICaL (dimensionless).
* ALGEBRAIC[83] is Iss in component ICaL (dimensionless).
* ALGEBRAIC[98] is Ii in component ICaL (dimensionless).
* CONSTANTS[53] is dielConstant in component ICaL (per_kelvin).
* CONSTANTS[134] is constA in component ICaL (dimensionless).
* CONSTANTS[139] is gamma_cao in component ICaL (dimensionless).
* ALGEBRAIC[84] is gamma_cass in component ICaL (dimensionless).
* ALGEBRAIC[101] is gamma_cai in component ICaL (dimensionless).
* CONSTANTS[140] is gamma_nao in component ICaL (dimensionless).
* ALGEBRAIC[85] is gamma_nass in component ICaL (dimensionless).
* ALGEBRAIC[104] is gamma_nai in component ICaL (dimensionless).
* CONSTANTS[141] is gamma_ko in component ICaL (dimensionless).
* ALGEBRAIC[86] is gamma_kss in component ICaL (dimensionless).
* ALGEBRAIC[105] is gamma_ki in component ICaL (dimensionless).
* CONSTANTS[54] is ICaL_fractionSS in component ICaL (dimensionless).
* CONSTANTS[55] is GKr_b in component IKr (milliS_per_microF).
* STATES[34] is C1 in component IKr (dimensionless).
* STATES[35] is C2 in component IKr (dimensionless).
* STATES[36] is C3 in component IKr (dimensionless).
* STATES[37] is I in component IKr (dimensionless).
* STATES[38] is O in component IKr (dimensionless).
* ALGEBRAIC[41] is alpha in component IKr (per_millisecond).
* ALGEBRAIC[47] is beta in component IKr (per_millisecond).
* CONSTANTS[56] is alpha_1 in component IKr (per_millisecond).
* CONSTANTS[57] is beta_1 in component IKr (per_millisecond).
* ALGEBRAIC[42] is alpha_2 in component IKr (per_millisecond).
* ALGEBRAIC[48] is beta_2 in component IKr (per_millisecond).
* ALGEBRAIC[52] is alpha_i in component IKr (per_millisecond).
* ALGEBRAIC[55] is beta_i in component IKr (per_millisecond).
* ALGEBRAIC[58] is alpha_C2ToI in component IKr (per_millisecond).
* ALGEBRAIC[62] is beta_ItoC2 in component IKr (per_millisecond).
* CONSTANTS[123] is GKr in component IKr (milliS_per_microF).
* CONSTANTS[58] is GKs_b in component IKs (milliS_per_microF).
* CONSTANTS[124] is GKs in component IKs (milliS_per_microF).
* ALGEBRAIC[12] is xs1ss in component IKs (dimensionless).
* ALGEBRAIC[26] is xs2ss in component IKs (dimensionless).
* ALGEBRAIC[27] is txs1 in component IKs (millisecond).
* STATES[39] is xs1 in component IKs (dimensionless).
* STATES[40] is xs2 in component IKs (dimensionless).
* ALGEBRAIC[116] is KsCa in component IKs (dimensionless).
* ALGEBRAIC[36] is txs2 in component IKs (millisecond).
* CONSTANTS[125] is GK1 in component IK1 (milliS_per_microF).
* CONSTANTS[59] is GK1_b in component IK1 (milliS_per_microF).
* ALGEBRAIC[118] is aK1 in component IK1 (dimensionless).
* ALGEBRAIC[119] is bK1 in component IK1 (dimensionless).

* ALGEBRAIC[120] is K1ss in component IK1 (dimensionless).
* CONSTANTS[60] is INaCa_fractionSS in component INaCa (dimensionless).
* CONSTANTS[61] is kna1 in component INaCa (per_millisecond).
* CONSTANTS[62] is kna2 in component INaCa (per_millisecond).
* CONSTANTS[63] is kna3 in component INaCa (per_millisecond).
* CONSTANTS[64] is kasymm in component INaCa (dimensionless).
* CONSTANTS[65] is wna in component INaCa (dimensionless).
* CONSTANTS[66] is wca in component INaCa (dimensionless).
* CONSTANTS[67] is wnaca in component INaCa (dimensionless).
* CONSTANTS[68] is kcaon in component INaCa (per_millisecond).
* CONSTANTS[69] is kcaoff in component INaCa (per_millisecond).
* CONSTANTS[70] is qna in component INaCa (dimensionless).
* CONSTANTS[71] is qca in component INaCa (dimensionless).
* ALGEBRAIC[123] is hna in component INaCa (dimensionless).
* ALGEBRAIC[122] is hca in component INaCa (dimensionless).
* CONSTANTS[72] is KmCaAct in component INaCa (millimolar).
* CONSTANTS[73] is Gncx_b in component INaCa (milliS_per_microF).
* CONSTANTS[152] is Gncx in component INaCa (milliS_per_microF).
* ALGEBRAIC[124] is h1_i in component INaCa (dimensionless).
* ALGEBRAIC[125] is h2_i in component INaCa (dimensionless).
* ALGEBRAIC[126] is h3_i in component INaCa (dimensionless).
* ALGEBRAIC[127] is h4_i in component INaCa (dimensionless).
* ALGEBRAIC[128] is h5_i in component INaCa (dimensionless).
* ALGEBRAIC[129] is h6_i in component INaCa (dimensionless).
* ALGEBRAIC[130] is h7_i in component INaCa (dimensionless).
* ALGEBRAIC[131] is h8_i in component INaCa (dimensionless).
* ALGEBRAIC[132] is h9_i in component INaCa (dimensionless).
* CONSTANTS[146] is h10_i in component INaCa (dimensionless).
* CONSTANTS[147] is h11_i in component INaCa (dimensionless).
* CONSTANTS[148] is h12_i in component INaCa (dimensionless).
* CONSTANTS[149] is k1_i in component INaCa (dimensionless).
* CONSTANTS[150] is k2_i in component INaCa (dimensionless).
* ALGEBRAIC[133] is k3p_i in component INaCa (dimensionless).
* ALGEBRAIC[134] is k3pp_i in component INaCa (dimensionless).
* ALGEBRAIC[135] is k3_i in component INaCa (dimensionless).
* ALGEBRAIC[138] is k4_i in component INaCa (dimensionless).
* ALGEBRAIC[136] is k4p_i in component INaCa (dimensionless).
* ALGEBRAIC[137] is k4pp_i in component INaCa (dimensionless).
* CONSTANTS[151] is k5_i in component INaCa (dimensionless).
* ALGEBRAIC[139] is k6_i in component INaCa (dimensionless).
* ALGEBRAIC[140] is k7_i in component INaCa (dimensionless).
* ALGEBRAIC[141] is k8_i in component INaCa (dimensionless).
* ALGEBRAIC[142] is x1_i in component INaCa (dimensionless).
* ALGEBRAIC[143] is x2_i in component INaCa (dimensionless).
* ALGEBRAIC[144] is x3_i in component INaCa (dimensionless).
* ALGEBRAIC[145] is x4_i in component INaCa (dimensionless).

* ALGEBRAIC[146] is E1_i in component INaCa (dimensionless).
* ALGEBRAIC[147] is E2_i in component INaCa (dimensionless).
* ALGEBRAIC[148] is E3_i in component INaCa (dimensionless).
* ALGEBRAIC[149] is E4_i in component INaCa (dimensionless).
* ALGEBRAIC[150] is allo_i in component INaCa (dimensionless).
* ALGEBRAIC[151] is JncxNa_i in component INaCa (millimolar_per_millisecond).
* ALGEBRAIC[152] is JncxCa_i in component INaCa (millimolar_per_millisecond).
* ALGEBRAIC[154] is h1_ss in component INaCa (dimensionless).
* ALGEBRAIC[155] is h2_ss in component INaCa (dimensionless).
* ALGEBRAIC[156] is h3_ss in component INaCa (dimensionless).
* ALGEBRAIC[157] is h4_ss in component INaCa (dimensionless).
* ALGEBRAIC[158] is h5_ss in component INaCa (dimensionless).
* ALGEBRAIC[159] is h6_ss in component INaCa (dimensionless).
* ALGEBRAIC[160] is h7_ss in component INaCa (dimensionless).
* ALGEBRAIC[161] is h8_ss in component INaCa (dimensionless).
* ALGEBRAIC[162] is h9_ss in component INaCa (dimensionless).
* CONSTANTS[153] is h10_ss in component INaCa (dimensionless).
* CONSTANTS[154] is h11_ss in component INaCa (dimensionless).
* CONSTANTS[155] is h12_ss in component INaCa (dimensionless).
* CONSTANTS[156] is k1_ss in component INaCa (dimensionless).
* CONSTANTS[157] is k2_ss in component INaCa (dimensionless).
* ALGEBRAIC[163] is k3p_ss in component INaCa (dimensionless).
* ALGEBRAIC[164] is k3pp_ss in component INaCa (dimensionless).
* ALGEBRAIC[165] is k3_ss in component INaCa (dimensionless).
* ALGEBRAIC[168] is k4_ss in component INaCa (dimensionless).
* ALGEBRAIC[166] is k4p_ss in component INaCa (dimensionless).
* ALGEBRAIC[167] is k4pp_ss in component INaCa (dimensionless).
* CONSTANTS[158] is k5_ss in component INaCa (dimensionless).
* ALGEBRAIC[169] is k6_ss in component INaCa (dimensionless).
* ALGEBRAIC[170] is k7_ss in component INaCa (dimensionless).
* ALGEBRAIC[171] is k8_ss in component INaCa (dimensionless).
* ALGEBRAIC[172] is x1_ss in component INaCa (dimensionless).
* ALGEBRAIC[173] is x2_ss in component INaCa (dimensionless).
* ALGEBRAIC[174] is x3_ss in component INaCa (dimensionless).
* ALGEBRAIC[175] is x4_ss in component INaCa (dimensionless).
* ALGEBRAIC[176] is E1_ss in component INaCa (dimensionless).
* ALGEBRAIC[177] is E2_ss in component INaCa (dimensionless).
* ALGEBRAIC[178] is E3_ss in component INaCa (dimensionless).
* ALGEBRAIC[179] is E4_ss in component INaCa (dimensionless).
* ALGEBRAIC[180] is allo_ss in component INaCa (dimensionless).
* ALGEBRAIC[181] is JncxNa_ss in component INaCa (millimolar_per_millisecond).
* ALGEBRAIC[182] is JncxCa_ss in component INaCa (millimolar_per_millisecond).
* CONSTANTS[74] is k1p in component INaK (per_millisecond).
* CONSTANTS[75] is k1m in component INaK (per_millisecond).
* CONSTANTS[76] is k2p in component INaK (per_millisecond).
* CONSTANTS[77] is k2m in component INaK (per_millisecond).

* CONSTANTS[78] is k3p in component INaK (per_millisecond).
* CONSTANTS[79] is k3m in component INaK (per_millisecond).
* CONSTANTS[80] is k4p in component INaK (per_millisecond).
* CONSTANTS[81] is k4m in component INaK (per_millisecond).
* CONSTANTS[82] is Knai0 in component INaK (millimolar).
* CONSTANTS[83] is Knao0 in component INaK (millimolar).
* CONSTANTS[84] is delta in component INaK (millivolt).
* CONSTANTS[85] is Kki in component INaK (per_millisecond).
* CONSTANTS[86] is Kko in component INaK (per_millisecond).
* CONSTANTS[87] is MgADP in component INaK (millimolar).
* CONSTANTS[88] is MgATP in component INaK (millimolar).
* CONSTANTS[89] is Kmgatp in component INaK (millimolar).
* CONSTANTS[90] is H in component INaK (millimolar).
* CONSTANTS[91] is eP in component INaK (dimensionless).
* CONSTANTS[92] is Khp in component INaK (millimolar).
* CONSTANTS[93] is Knap in component INaK (millimolar).
* CONSTANTS[94] is Kxkur in component INaK (millimolar).
* CONSTANTS[95] is Pnak_b in component INaK (milliS_per_microF).
* CONSTANTS[162] is Pnak in component INaK (milliS_per_microF).
* ALGEBRAIC[184] is Knai in component INaK (millimolar).
* ALGEBRAIC[185] is Knao in component INaK (millimolar).
* ALGEBRAIC[186] is P in component INaK (dimensionless).
* ALGEBRAIC[187] is a1 in component INaK (dimensionless).
* CONSTANTS[159] is b1 in component INaK (dimensionless).
* CONSTANTS[160] is a2 in component INaK (dimensionless).
* ALGEBRAIC[188] is b2 in component INaK (dimensionless).
* ALGEBRAIC[189] is a3 in component INaK (dimensionless).
* ALGEBRAIC[190] is b3 in component INaK (dimensionless).
* CONSTANTS[161] is a4 in component INaK (dimensionless).
* ALGEBRAIC[191] is b4 in component INaK (dimensionless).
* ALGEBRAIC[192] is x1 in component INaK (dimensionless).
* ALGEBRAIC[193] is x2 in component INaK (dimensionless).
* ALGEBRAIC[194] is x3 in component INaK (dimensionless).
* ALGEBRAIC[195] is x4 in component INaK (dimensionless).
* ALGEBRAIC[196] is E1 in component INaK (dimensionless).
* ALGEBRAIC[197] is E2 in component INaK (dimensionless).
* ALGEBRAIC[198] is E3 in component INaK (dimensionless).
* ALGEBRAIC[199] is E4 in component INaK (dimensionless).
* ALGEBRAIC[200] is JnakNa in component INaK (millimolar_per_millisecond).
* ALGEBRAIC[201] is JnakK in component INaK (millimolar_per_millisecond).
* ALGEBRAIC[203] is xkb in component IKb (dimensionless).
* CONSTANTS[96] is GKb_b in component IKb (milliS_per_microF).
* CONSTANTS[126] is GKb in component IKb (milliS_per_microF).
* CONSTANTS[97] is PNab in component INab (milliS_per_microF).
* CONSTANTS[98] is PCab in component ICab (milliS_per_microF).
* CONSTANTS[99] is GpCa in component IpCa (milliS_per_microF).

* CONSTANTS[100] is KmCap in component IpCa (millimolar).
* CONSTANTS[101] is GClCa in component ICl (milliS_per_microF).
* CONSTANTS[102] is GClb in component ICl (milliS_per_microF).
* CONSTANTS[103] is KdClCa in component ICl (millimolar).
* CONSTANTS[104] is Fjunc in component ICl (dimensionless).
* ALGEBRAIC[210] is IClCa_junc in component ICl (microA_per_microF).
* ALGEBRAIC[212] is IClCa_sl in component ICl (microA_per_microF).
* CONSTANTS[105] is tauNa in component diff (millisecond).
* CONSTANTS[106] is tauK in component diff (millisecond).
* CONSTANTS[107] is tauCa in component diff (millisecond).
* CONSTANTS[108] is bt in component ryr (millisecond).
* CONSTANTS[127] is a_rel in component ryr (millimolar_per_millisecond).
* ALGEBRAIC[93] is Jrel_inf_b in component ryr (millimolar_per_millisecond).
* ALGEBRAIC[96] is Jrel_inf in component ryr (millimolar_per_millisecond).
* ALGEBRAIC[99] is tau_rel_b in component ryr (millisecond).
* ALGEBRAIC[102] is tau_rel in component ryr (millisecond).
* STATES[41] is Jrel_np in component ryr (millimolar_per_millisecond).
* CONSTANTS[128] is btp in component ryr (millisecond).
* CONSTANTS[135] is a_relp in component ryr (millimolar_per_millisecond).
* ALGEBRAIC[94] is Jrel_infp_b in component ryr (millimolar_per_millisecond).
* ALGEBRAIC[97] is Jrel_infp in component ryr (millimolar_per_millisecond).
* ALGEBRAIC[100] is tau_relp_b in component ryr (millisecond).
* ALGEBRAIC[103] is tau_relp in component ryr (millisecond).
* STATES[42] is Jrel_p in component ryr (millimolar_per_millisecond).
* CONSTANTS[109] is cajsr_half in component ryr (millimolar).
* ALGEBRAIC[213] is fJrelp in component ryr (dimensionless).
* CONSTANTS[110] is Jrel_b in component ryr (dimensionless).
* CONSTANTS[129] is upScale in component SERCA (dimensionless).
* ALGEBRAIC[217] is Jupnp in component SERCA (millimolar_per_millisecond).
* ALGEBRAIC[218] is Jupp in component SERCA (millimolar_per_millisecond).
* ALGEBRAIC[219] is fJupp in component SERCA (dimensionless).
* ALGEBRAIC[220] is Jleak in component SERCA (millimolar_per_millisecond).
* CONSTANTS[111] is Jup_b in component SERCA (dimensionless).
* RATES[0] is d/dt v in component membrane (millivolt).
* RATES[1] is d/dt CaMKt in component CaMK (millimolar).
* RATES[3] is d/dt nai in component intracellular_ions (millimolar).
* RATES[4] is d/dt nass in component intracellular_ions (millimolar).
* RATES[5] is d/dt ki in component intracellular_ions (millimolar).
* RATES[6] is d/dt kss in component intracellular_ions (millimolar).
* RATES[9] is d/dt cai in component intracellular_ions (millimolar).
* RATES[2] is d/dt cass in component intracellular_ions (millimolar).
* RATES[7] is d/dt cansr in component intracellular_ions (millimolar).
* RATES[8] is d/dt cajsr in component intracellular_ions (millimolar).
* RATES[10] is d/dt m in component INa (dimensionless).
* RATES[11] is d/dt h in component INa (dimensionless).
* RATES[12] is d/dt j in component INa (dimensionless).

```
 * RATES[13] is d/dt hp in component INa (dimensionless).
 * RATES[14] is d/dt jp in component INa (dimensionless).
 * RATES[15] is d/dt mL in component INaL (dimensionless).
 * RATES[16] is d/dt hL in component INaL (dimensionless).
 * RATES[17] is d/dt hLp in component INaL (dimensionless).
 * RATES[18] is d/dt a in component Ito (dimensionless).
 * RATES[19] is d/dt iF in component Ito (dimensionless).
 * RATES[20] is d/dt iS in component Ito (dimensionless).
 * RATES[21] is d/dt ap in component Ito (dimensionless).
 * RATES[22] is d/dt iFp in component Ito (dimensionless).
 * RATES[23] is d/dt iSp in component Ito (dimensionless).
 * RATES[24] is d/dt d in component ICaL (dimensionless).
 * RATES[25] is d/dt ff in component ICaL (dimensionless).
 * RATES[26] is d/dt fs in component ICaL (dimensionless).
 * RATES[27] is d/dt fcaf in component ICaL (dimensionless).
 * RATES[28] is d/dt fcas in component ICaL (dimensionless).
 * RATES[29] is d/dt jca in component ICaL (dimensionless).
 * RATES[30] is d/dt ffp in component ICaL (dimensionless).
 * RATES[31] is d/dt fcafp in component ICaL (dimensionless).
 * RATES[32] is d/dt nca_ss in component ICaL (dimensionless).
 * RATES[33] is d/dt nca_i in component ICaL (dimensionless).
 * RATES[36] is d/dt C3 in component IKr (dimensionless).
 * RATES[35] is d/dt C2 in component IKr (dimensionless).
 * RATES[34] is d/dt C1 in component IKr (dimensionless).
 * RATES[38] is d/dt O in component IKr (dimensionless).
 * RATES[37] is d/dt I in component IKr (dimensionless).
 * RATES[39] is d/dt xs1 in component IKs (dimensionless).
 * RATES[40] is d/dt xs2 in component IKs (dimensionless).
 * RATES[41] is d/dt Jrel_np in component ryr (millimolar_per_millisecond).
 * RATES[42] is d/dt Jrel_p in component ryr (millimolar_per_millisecond).
 */

void CLASS_NAME::initConsts()
{
CONSTANTS[0] = 0;
CONSTANTS[1] = 140.0;
CONSTANTS[2] = 1.8;
CONSTANTS[3] = 5.0;
CONSTANTS[4] = 150.0;
CONSTANTS[5] = 8314;
CONSTANTS[6] = 310;
CONSTANTS[7] = 96485;
CONSTANTS[8] = 1;
CONSTANTS[9] = 2;
CONSTANTS[10] = 1;
CONSTANTS[11] = -1;
```

```
CONSTANTS[12] = 0.01;
CONSTANTS[13] = 0.0011;
STATES[0] = -88.7638;
CONSTANTS[14] = 0;
CONSTANTS[15] = 100000000000000000;
CONSTANTS[16] = -53;
CONSTANTS[17] = 1000;
CONSTANTS[18] = 1.0;
CONSTANTS[19] = 0.15;
CONSTANTS[20] = 0.05;
CONSTANTS[21] = 0.00068;
CONSTANTS[22] = 0.05;
CONSTANTS[23] = 0.0015;
STATES[1] = 0.0111;
STATES[2] = 7.0305e-5;
CONSTANTS[24] = 0.05;
CONSTANTS[25] = 0.00238;
CONSTANTS[26] = 0.07;
CONSTANTS[27] = 0.0005;
CONSTANTS[28] = 0.047;
CONSTANTS[29] = 0.00087;
CONSTANTS[30] = 1.124;
CONSTANTS[31] = 0.0087;
CONSTANTS[32] = 10;
CONSTANTS[33] = 0.8;
STATES[3] = 12.1025;
STATES[4] = 12.1029;
STATES[5] = 142.3002;
STATES[6] = 142.3002;
STATES[7] = 1.5211;
STATES[8] = 1.5214;
STATES[9] = 8.1583e-05;
CONSTANTS[34] = 24.0;
CONSTANTS[35] = 0.01833;
CONSTANTS[36] = 4.3195;
CONSTANTS[37] = 0.0;
CONSTANTS[38] = 5;
CONSTANTS[39] = 2;
CONSTANTS[40] = 0.25;
STATES[10] = 8.0572e-4;
STATES[11] = 0.8286;
STATES[12] = 0.8284;
STATES[13] = 0.6707;
STATES[14] = 0.8281;
CONSTANTS[41] = 11.7802;
STATES[15] = 1.629e-4;
```

```
CONSTANTS[42] = 200;
STATES[16] = 0.5255;
STATES[17] = 0.2872;
CONSTANTS[43] = 0.0279;
CONSTANTS[44] = 0.16;
STATES[18] = 9.5098e-4;
CONSTANTS[45] = 0;
STATES[19] = 0.9996;
STATES[20] = 0.5936;
STATES[21] = 4.8454e-4;
STATES[22] = 0.9996;
STATES[23] = 0.6538;
CONSTANTS[46] = 0.002;
CONSTANTS[47] = 500;
CONSTANTS[48] = 8.3757e-05;
STATES[24] = 8.1084e-9;
CONSTANTS[49] = 0.6;
STATES[25] = 1.0;
STATES[26] = 0.939;
STATES[27] = 1.0;
STATES[28] = 0.9999;
STATES[29] = 1.0;
STATES[30] = 1.0;
STATES[31] = 1.0;
STATES[32] = 6.6462e-4;
STATES[33] = 0.0012;
CONSTANTS[50] = 75;
CONSTANTS[51] = 0;
CONSTANTS[52] = 0;
CONSTANTS[53] = 74;
CONSTANTS[54] = 0.8;
CONSTANTS[55] = 0.0321;
STATES[34] = 7.0344e-4;
STATES[35] = 8.5109e-4;
STATES[36] = 0.9981;
STATES[37] = 1.3289e-5;
STATES[38] = 3.7585e-4;
CONSTANTS[56] = 0.154375;
CONSTANTS[57] = 0.1911;
CONSTANTS[58] = 0.0011;
STATES[39] = 0.248;
STATES[40] = 1.7707e-4;
CONSTANTS[59] = 0.6992;
CONSTANTS[60] = 0.35;
CONSTANTS[61] = 15;
CONSTANTS[62] = 5;
```

```
CONSTANTS[63] = 88.12;
CONSTANTS[64] = 12.5;
CONSTANTS[65] = 6e4;
CONSTANTS[66] = 6e4;
CONSTANTS[67] = 5e3;
CONSTANTS[68] = 1.5e6;
CONSTANTS[69] = 5e3;
CONSTANTS[70] = 0.5224;
CONSTANTS[71] = 0.167;
CONSTANTS[72] = 150e-6;
CONSTANTS[73] = 0.0034;
CONSTANTS[74] = 949.5;
CONSTANTS[75] = 182.4;
CONSTANTS[76] = 687.2;
CONSTANTS[77] = 39.4;
CONSTANTS[78] = 1899;
CONSTANTS[79] = 79300;
CONSTANTS[80] = 639;
CONSTANTS[81] = 40;
CONSTANTS[82] = 9.073;
CONSTANTS[83] = 27.78;
CONSTANTS[84] = -0.155;
CONSTANTS[85] = 0.5;
CONSTANTS[86] = 0.3582;
CONSTANTS[87] = 0.05;
CONSTANTS[88] = 9.8;
CONSTANTS[89] = 1.698e-7;
CONSTANTS[90] = 1e-7;
CONSTANTS[91] = 4.2;
CONSTANTS[92] = 1.698e-7;
CONSTANTS[93] = 224;
CONSTANTS[94] = 292;
CONSTANTS[95] = 15.4509;
CONSTANTS[96] = 0.0189;
CONSTANTS[97] = 1.9239e-09;
CONSTANTS[98] = 5.9194e-08;
CONSTANTS[99] = 5e-04;
CONSTANTS[100] = 0.0005;
CONSTANTS[101] = 0.2843;
CONSTANTS[102] = 1.98e-3;
CONSTANTS[103] = 0.1;
CONSTANTS[104] = 1;
CONSTANTS[105] = 2.0;
CONSTANTS[106] = 2.0;
CONSTANTS[107] = 0.2;
CONSTANTS[108] = 4.75;
```

```
STATES[41] = 1.6129e-22;
STATES[42] = 1.2475e-20;
CONSTANTS[109] = 1.7;
CONSTANTS[110] = 1.5378;
CONSTANTS[111] = 1.0;
CONSTANTS[112] =  1000.00*3.14000*CONSTANTS[13]*CONSTANTS[13]*CONSTANTS[12];
CONSTANTS[113] = (CONSTANTS[0]==1.00000 ?  CONSTANTS[24]*1.30000 : CONSTANTS[24]);
CONSTANTS[114] =
(( CONSTANTS[5]*CONSTANTS[6])/( CONSTANTS[11]*CONSTANTS[7]))*log(CONSTANTS[4]/CO
NSTANTS[34]);
CONSTANTS[115] = pow(CONSTANTS[3]/CONSTANTS[38], 0.240000);
CONSTANTS[116] = 1.00000/(1.00000+pow(CONSTANTS[39]/CONSTANTS[40], 2.00000));
CONSTANTS[117] =  3.00000*CONSTANTS[42];
CONSTANTS[118] = (CONSTANTS[0]==1.00000 ?  CONSTANTS[43]*0.600000 :
CONSTANTS[43]);
CONSTANTS[119] = (CONSTANTS[0]==1.00000 ?  CONSTANTS[44]*2.00000 :
CONSTANTS[0]==2.00000 ?  CONSTANTS[44]*2.00000 : CONSTANTS[44]);
CONSTANTS[120] = 1.00000 - CONSTANTS[49];
CONSTANTS[121] = (CONSTANTS[0]==1.00000 ?  CONSTANTS[48]*1.20000 :
CONSTANTS[0]==2.00000 ?  CONSTANTS[48]*2.00000 : CONSTANTS[48]);
CONSTANTS[122] = ( 0.500000*(CONSTANTS[1]+CONSTANTS[3]+CONSTANTS[4]+
4.00000*CONSTANTS[2]))/1000.00;
CONSTANTS[123] = (CONSTANTS[0]==1.00000 ?  CONSTANTS[55]*1.30000 :
CONSTANTS[0]==2.00000 ?  CONSTANTS[55]*0.800000 : CONSTANTS[55]);
CONSTANTS[124] = (CONSTANTS[0]==1.00000 ?  CONSTANTS[58]*1.40000 : CONSTANTS[58]);
CONSTANTS[125] = (CONSTANTS[0]==1.00000 ?  CONSTANTS[59]*1.20000 :
CONSTANTS[0]==2.00000 ?  CONSTANTS[59]*1.30000 : CONSTANTS[59]);
CONSTANTS[126] = (CONSTANTS[0]==1.00000 ?  CONSTANTS[96]*0.600000 :
CONSTANTS[96]);
CONSTANTS[127] = ( 0.500000*CONSTANTS[108])/1.00000;
CONSTANTS[128] =  1.25000*CONSTANTS[108];
CONSTANTS[129] = (CONSTANTS[0]==1.00000 ? 1.30000 : 1.00000);
CONSTANTS[130] =  2.00000*3.14000*CONSTANTS[13]*CONSTANTS[13]+
2.00000*3.14000*CONSTANTS[13]*CONSTANTS[12];
CONSTANTS[131] =  1.10000*CONSTANTS[121];
CONSTANTS[132] =  0.00125000*CONSTANTS[121];
CONSTANTS[133] =  0.000357400*CONSTANTS[121];
CONSTANTS[134] =  1.82000e+06*pow( CONSTANTS[53]*CONSTANTS[6], - 1.50000);
CONSTANTS[135] = ( 0.500000*CONSTANTS[128])/1.00000;
CONSTANTS[136] =  2.00000*CONSTANTS[130];
CONSTANTS[137] =  0.00125000*CONSTANTS[131];
CONSTANTS[138] =  0.000357400*CONSTANTS[131];
CONSTANTS[139] = exp( - CONSTANTS[134]*4.00000*( pow(CONSTANTS[122], 1.0 /
2)/(1.00000+ pow(CONSTANTS[122], 1.0 / 2)) -  0.300000*CONSTANTS[122]));
CONSTANTS[140] = exp( - CONSTANTS[134]*1.00000*( pow(CONSTANTS[122], 1.0 /
2)/(1.00000+ pow(CONSTANTS[122], 1.0 / 2)) -  0.300000*CONSTANTS[122]));
```

```
CONSTANTS[141] = exp( - CONSTANTS[134]*1.00000*( pow(CONSTANTS[122], 1.0 / 2)/(1.00000+ pow(CONSTANTS[122], 1.0 / 2)) -  0.300000*CONSTANTS[122]));
CONSTANTS[142] =  0.680000*CONSTANTS[112];
CONSTANTS[143] =  0.0552000*CONSTANTS[112];
CONSTANTS[144] =  0.00480000*CONSTANTS[112];
CONSTANTS[145] =  0.0200000*CONSTANTS[112];
CONSTANTS[146] = CONSTANTS[64]+1.00000+
(CONSTANTS[1]/CONSTANTS[61])*(1.00000+CONSTANTS[1]/CONSTANTS[62]);
CONSTANTS[147] =
( CONSTANTS[1]*CONSTANTS[1])/( CONSTANTS[146]*CONSTANTS[61]*CONSTANTS[62]);
CONSTANTS[148] = 1.00000/CONSTANTS[146];
CONSTANTS[149] =  CONSTANTS[148]*CONSTANTS[2]*CONSTANTS[68];
CONSTANTS[150] = CONSTANTS[69];
CONSTANTS[151] = CONSTANTS[69];
CONSTANTS[152] = (CONSTANTS[0]==1.00000 ?  CONSTANTS[73]*1.10000 :
CONSTANTS[0]==2.00000 ?  CONSTANTS[73]*1.40000 : CONSTANTS[73]);
CONSTANTS[153] = CONSTANTS[64]+1.00000+
(CONSTANTS[1]/CONSTANTS[61])*(1.00000+CONSTANTS[1]/CONSTANTS[62]);
CONSTANTS[154] =
( CONSTANTS[1]*CONSTANTS[1])/( CONSTANTS[153]*CONSTANTS[61]*CONSTANTS[62]);
CONSTANTS[155] = 1.00000/CONSTANTS[153];
CONSTANTS[156] =  CONSTANTS[155]*CONSTANTS[2]*CONSTANTS[68];
CONSTANTS[157] = CONSTANTS[69];
CONSTANTS[158] = CONSTANTS[69];
CONSTANTS[159] =  CONSTANTS[75]*CONSTANTS[87];
CONSTANTS[160] = CONSTANTS[76];
CONSTANTS[161] =
(( CONSTANTS[80]*CONSTANTS[88])/CONSTANTS[89])/(1.00000+CONSTANTS[88]/CONSTANTS[89]);
CONSTANTS[162] = (CONSTANTS[0]==1.00000 ?  CONSTANTS[95]*0.900000 :
CONSTANTS[0]==2.00000 ?  CONSTANTS[95]*0.700000 : CONSTANTS[95]);
}

void CLASS_NAME::computeRates(double TIME, double* CONSTANTS, double* RATES,
double* STATES, double* ALGEBRAIC)
{
ALGEBRAIC[3] = 1.00000/(1.00000+exp((STATES[0]+87.6100)/7.48800));
RATES[16] = (ALGEBRAIC[3] - STATES[16])/CONSTANTS[42];
ALGEBRAIC[4] = 1.00000/(1.00000+exp((STATES[0]+93.8100)/7.48800));
RATES[17] = (ALGEBRAIC[4] - STATES[17])/CONSTANTS[117];
ALGEBRAIC[9] = 1.00000/(1.00000+exp((STATES[0]+18.0800)/2.79160));
RATES[29] = (ALGEBRAIC[9] - STATES[29])/CONSTANTS[50];
ALGEBRAIC[0] = 1.00000/pow(1.00000+exp(- (STATES[0]+56.8600)/9.03000), 2.00000);
ALGEBRAIC[13] =  0.129200*exp(- pow((STATES[0]+45.7900)/15.5400, 2.00000))+
0.0648700*exp(- pow((STATES[0] - 4.82300)/51.1200, 2.00000));
RATES[10] = (ALGEBRAIC[0] - STATES[10])/ALGEBRAIC[13];
```

```
ALGEBRAIC[2] = 1.00000/(1.00000+exp(- (STATES[0]+42.8500)/5.26400));
ALGEBRAIC[16] =  0.129200*exp(- pow((STATES[0]+45.7900)/15.5400, 2.00000))+
0.0648700*exp(- pow((STATES[0] - 4.82300)/51.1200, 2.00000));
RATES[15] = (ALGEBRAIC[2] - STATES[15])/ALGEBRAIC[16];
ALGEBRAIC[5] = 1.00000/(1.00000+exp(- ((STATES[0]+CONSTANTS[45]) - 14.3400)/14.8200));
ALGEBRAIC[17] = 1.05150/(1.00000/( 1.20890*(1.00000+exp(- ((STATES[0]+CONSTANTS[45]) -
18.4099)/29.3814)))+3.50000/(1.00000+exp((STATES[0]+CONSTANTS[45]+100.000)/29.3814))
);
RATES[18] = (ALGEBRAIC[5] - STATES[18])/ALGEBRAIC[17];
ALGEBRAIC[7] = (STATES[0]>=31.4978 ? 1.00000 :  1.07630*exp( - 1.00700*exp( -
0.0829000*STATES[0])));
ALGEBRAIC[22] = CONSTANTS[52]+0.600000+1.00000/(exp( -
0.0500000*(STATES[0]+CONSTANTS[51]+6.00000))+exp( 0.0900000*(STATES[0]+CONSTANTS[
51]+14.0000)));
RATES[24] = (ALGEBRAIC[7] - STATES[24])/ALGEBRAIC[22];
ALGEBRAIC[8] = 1.00000/(1.00000+exp((STATES[0]+19.5800)/3.69600));
ALGEBRAIC[23] = 7.00000+1.00000/( 0.00450000*exp(- (STATES[0]+20.0000)/10.0000)+
0.00450000*exp((STATES[0]+20.0000)/10.0000));
RATES[25] = (ALGEBRAIC[8] - STATES[25])/ALGEBRAIC[23];
ALGEBRAIC[24] = 1000.00+1.00000/( 3.50000e-05*exp(- (STATES[0]+5.00000)/4.00000)+
3.50000e-05*exp((STATES[0]+5.00000)/6.00000));
RATES[26] = (ALGEBRAIC[8] - STATES[26])/ALGEBRAIC[24];
ALGEBRAIC[10] =  STATES[29]*1.00000;
ALGEBRAIC[20] =
1.00000/(CONSTANTS[47]/ALGEBRAIC[10]+pow(1.00000+CONSTANTS[46]/STATES[2],
4.00000));
RATES[32] =  ALGEBRAIC[20]*CONSTANTS[47] -  STATES[32]*ALGEBRAIC[10];
ALGEBRAIC[21] =
1.00000/(CONSTANTS[47]/ALGEBRAIC[10]+pow(1.00000+CONSTANTS[46]/STATES[9],
4.00000));
RATES[33] =  ALGEBRAIC[21]*CONSTANTS[47] -  STATES[33]*ALGEBRAIC[10];
ALGEBRAIC[12] = 1.00000/(1.00000+exp(- (STATES[0]+11.6000)/8.93200));
ALGEBRAIC[27] = 817.300+1.00000/( 0.000232600*exp((STATES[0]+48.2800)/17.8000)+
0.00129200*exp(- (STATES[0]+210.000)/230.000));
RATES[39] = (ALGEBRAIC[12] - STATES[39])/ALGEBRAIC[27];
ALGEBRAIC[32] = 1.00000/(1.00000+exp(- ((STATES[0]+CONSTANTS[45]) - 24.3400)/14.8200));
RATES[21] = (ALGEBRAIC[32] - STATES[21])/ALGEBRAIC[17];
ALGEBRAIC[19] = ALGEBRAIC[8];
ALGEBRAIC[33] = 7.00000+1.00000/( 0.0400000*exp(- (STATES[0] - 4.00000)/7.00000)+
0.0400000*exp((STATES[0] - 4.00000)/7.00000));
RATES[27] = (ALGEBRAIC[19] - STATES[27])/ALGEBRAIC[33];
ALGEBRAIC[34] = 100.000+1.00000/( 0.000120000*exp(- STATES[0]/3.00000)+
0.000120000*exp(STATES[0]/7.00000));
RATES[28] = (ALGEBRAIC[19] - STATES[28])/ALGEBRAIC[34];
ALGEBRAIC[35] =  2.50000*ALGEBRAIC[23];
RATES[30] = (ALGEBRAIC[8] - STATES[30])/ALGEBRAIC[35];
```

```
ALGEBRAIC[26] = ALGEBRAIC[12];
ALGEBRAIC[36] = 1.00000/( 0.0100000*exp((STATES[0] - 50.0000)/20.0000)+ 0.0193000*exp(-(STATES[0]+66.5400)/31.0000));
RATES[40] = (ALGEBRAIC[26] - STATES[40])/ALGEBRAIC[36];
ALGEBRAIC[43] = ( CONSTANTS[22]*(1.00000 - STATES[1]))/(1.00000+CONSTANTS[23]/STATES[2]);
RATES[1] =  CONSTANTS[20]*ALGEBRAIC[43]*(ALGEBRAIC[43]+STATES[1]) - CONSTANTS[21]*STATES[1];
ALGEBRAIC[1] = 1.00000/pow(1.00000+exp((STATES[0]+71.5500)/7.43000), 2.00000);
ALGEBRAIC[14] = (STATES[0]>=- 40.0000 ? 0.00000 :  0.0570000*exp(-(STATES[0]+80.0000)/6.80000));
ALGEBRAIC[29] = (STATES[0]>=- 40.0000 ? 0.770000/( 0.130000*(1.00000+exp(-(STATES[0]+10.6600)/11.1000))) :  2.70000*exp( 0.0790000*STATES[0])+ 310000.*exp( 0.348500*STATES[0]));
ALGEBRAIC[37] = 1.00000/(ALGEBRAIC[14]+ALGEBRAIC[29]);
RATES[11] = (ALGEBRAIC[1] - STATES[11])/ALGEBRAIC[37];
ALGEBRAIC[40] =  2.50000*ALGEBRAIC[33];
RATES[31] = (ALGEBRAIC[19] - STATES[31])/ALGEBRAIC[40];
ALGEBRAIC[38] = ALGEBRAIC[1];
ALGEBRAIC[15] = (STATES[0]>=- 40.0000 ? 0.00000 : ( ( - 25428.0*exp( 0.244400*STATES[0]) - 6.94800e-06*exp( - 0.0439100*STATES[0]))*(STATES[0]+37.7800))/(1.00000+exp( 0.311000*(STATES[0]+79.2300))));
ALGEBRAIC[30] = (STATES[0]>=- 40.0000 ? ( 0.600000*exp( 0.0570000*STATES[0]))/(1.00000+exp( - 0.100000*(STATES[0]+32.0000))) : ( 0.0242400*exp( - 0.0105200*STATES[0]))/(1.00000+exp( - 0.137800*(STATES[0]+40.1400))));
ALGEBRAIC[44] = 1.00000/(ALGEBRAIC[15]+ALGEBRAIC[30]);
RATES[12] = (ALGEBRAIC[38] - STATES[12])/ALGEBRAIC[44];
ALGEBRAIC[45] = 1.00000/pow(1.00000+exp((STATES[0]+77.5500)/7.43000), 2.00000);
RATES[13] = (ALGEBRAIC[45] - STATES[13])/ALGEBRAIC[37];
ALGEBRAIC[6] = 1.00000/(1.00000+exp((STATES[0]+CONSTANTS[45]+43.9400)/5.71100));
ALGEBRAIC[18] = (CONSTANTS[0]==1.00000 ? 1.00000 - 0.950000/(1.00000+exp((STATES[0]+CONSTANTS[45]+70.0000)/5.00000)) : 1.00000);
ALGEBRAIC[31] = 4.56200+1.00000/( 0.393300*exp(-(STATES[0]+CONSTANTS[45]+100.000)/100.000)+ 0.0800400*exp((STATES[0]+CONSTANTS[45]+50.0000)/16.5900));
ALGEBRAIC[46] =  ALGEBRAIC[31]*ALGEBRAIC[18];
RATES[19] = (ALGEBRAIC[6] - STATES[19])/ALGEBRAIC[46];
ALGEBRAIC[28] = ( STATES[0]*CONSTANTS[7])/( CONSTANTS[5]*CONSTANTS[6]);
ALGEBRAIC[41] =  0.116100*exp( 0.299000*ALGEBRAIC[28]);
ALGEBRAIC[47] =  0.244200*exp( - 1.60400*ALGEBRAIC[28]);
RATES[36] =  ALGEBRAIC[47]*STATES[35] -  ALGEBRAIC[41]*STATES[36];
RATES[35] = ( ALGEBRAIC[41]*STATES[36]+ CONSTANTS[57]*STATES[34]) - (ALGEBRAIC[47]+CONSTANTS[56])*STATES[35];
ALGEBRAIC[50] =  1.46000*ALGEBRAIC[44];
```

```
RATES[14] = (ALGEBRAIC[38] - STATES[14])/ALGEBRAIC[50];
ALGEBRAIC[39] = 23.6200+1.00000/( 0.00141600*exp(-
(STATES[0]+CONSTANTS[45]+96.5200)/59.0500)+ 1.78000e-
08*exp((STATES[0]+CONSTANTS[45]+114.100)/8.07900));
ALGEBRAIC[51] =  ALGEBRAIC[39]*ALGEBRAIC[18];
RATES[20] = (ALGEBRAIC[6] - STATES[20])/ALGEBRAIC[51];
ALGEBRAIC[42] =  0.0578000*exp( 0.971000*ALGEBRAIC[28]);
ALGEBRAIC[48] =  0.000349000*exp( - 1.06200*ALGEBRAIC[28]);
ALGEBRAIC[52] =  0.253300*exp( 0.595300*ALGEBRAIC[28]);
ALGEBRAIC[55] =  0.0652500*exp( - 0.820900*ALGEBRAIC[28]);
RATES[38] = ( ALGEBRAIC[42]*STATES[34]+ ALGEBRAIC[55]*STATES[37]) -
(ALGEBRAIC[48]+ALGEBRAIC[52])*STATES[38];
ALGEBRAIC[54] = 1.35400+0.000100000/(exp(((STATES[0]+CONSTANTS[45]) -
167.400)/15.8900)+exp(- ((STATES[0]+CONSTANTS[45]) - 12.2300)/0.215400));
ALGEBRAIC[57] = 1.00000 -
0.500000/(1.00000+exp((STATES[0]+CONSTANTS[45]+70.0000)/20.0000));
ALGEBRAIC[60] =  ALGEBRAIC[54]*ALGEBRAIC[57]*ALGEBRAIC[46];
RATES[22] = (ALGEBRAIC[6] - STATES[22])/ALGEBRAIC[60];
ALGEBRAIC[61] =  ALGEBRAIC[54]*ALGEBRAIC[57]*ALGEBRAIC[51];
RATES[23] = (ALGEBRAIC[6] - STATES[23])/ALGEBRAIC[61];
ALGEBRAIC[58] =  5.20000e-05*exp( 1.52500*ALGEBRAIC[28]);
ALGEBRAIC[62] =
( ALGEBRAIC[48]*ALGEBRAIC[55]*ALGEBRAIC[58])/( ALGEBRAIC[42]*ALGEBRAIC[52]);
RATES[34] = ( CONSTANTS[56]*STATES[35]+ ALGEBRAIC[48]*STATES[38]+
ALGEBRAIC[62]*STATES[37]) -
(CONSTANTS[57]+ALGEBRAIC[42]+ALGEBRAIC[58])*STATES[34];
RATES[37] = ( ALGEBRAIC[58]*STATES[34]+ ALGEBRAIC[52]*STATES[38]) -
(ALGEBRAIC[62]+ALGEBRAIC[55])*STATES[37];
ALGEBRAIC[77] =  CONSTANTS[49]*STATES[25]+ CONSTANTS[120]*STATES[26];
ALGEBRAIC[78] = 0.300000+0.600000/(1.00000+exp((STATES[0] - 10.0000)/10.0000));
ALGEBRAIC[79] = 1.00000 - ALGEBRAIC[78];
ALGEBRAIC[80] =  ALGEBRAIC[78]*STATES[27]+ ALGEBRAIC[79]*STATES[28];
ALGEBRAIC[81] =  CONSTANTS[49]*STATES[30]+ CONSTANTS[120]*STATES[26];
ALGEBRAIC[82] =  ALGEBRAIC[78]*STATES[31]+ ALGEBRAIC[79]*STATES[28];
ALGEBRAIC[25] =
( STATES[0]*CONSTANTS[7]*CONSTANTS[7])/( CONSTANTS[5]*CONSTANTS[6]);
ALGEBRAIC[83] = ( 0.500000*(STATES[4]+STATES[6]+CONSTANTS[34]+
4.00000*STATES[2]))/1000.00;
ALGEBRAIC[84] = exp( - CONSTANTS[134]*4.00000*( pow(ALGEBRAIC[83], 1.0 / 2)/(1.00000+
pow(ALGEBRAIC[83], 1.0 / 2)) -  0.300000*ALGEBRAIC[83]));
ALGEBRAIC[87] =
( 4.00000*ALGEBRAIC[25]*( ALGEBRAIC[84]*STATES[2]*exp( 2.00000*ALGEBRAIC[28]) -
CONSTANTS[139]*CONSTANTS[2]))/(exp( 2.00000*ALGEBRAIC[28]) - 1.00000);
ALGEBRAIC[49] = ALGEBRAIC[43]+STATES[1];
ALGEBRAIC[90] = 1.00000/(1.00000+CONSTANTS[19]/ALGEBRAIC[49]);
```

```
ALGEBRAIC[91] =  CONSTANTS[54]*( (1.00000 - 
ALGEBRAIC[90])*CONSTANTS[121]*ALGEBRAIC[87]*STATES[24]*( ALGEBRAIC[77]*(1.00000 - 
STATES[32])+ STATES[29]*ALGEBRAIC[80]*STATES[32])+ 
ALGEBRAIC[90]*CONSTANTS[131]*ALGEBRAIC[87]*STATES[24]*( ALGEBRAIC[81]*(1.00000 - 
STATES[32])+ STATES[29]*ALGEBRAIC[82]*STATES[32]));
ALGEBRAIC[93] = (( - 
CONSTANTS[127]*ALGEBRAIC[91])/1.00000)/(1.00000+pow(CONSTANTS[109]/STATES[8], 
8.00000));
ALGEBRAIC[96] = (CONSTANTS[0]==2.00000 ?  ALGEBRAIC[93]*1.70000 : ALGEBRAIC[93]);
ALGEBRAIC[99] = CONSTANTS[108]/(1.00000+0.0123000/STATES[8]);
ALGEBRAIC[102] = (ALGEBRAIC[99]<0.00100000 ? 0.00100000 : ALGEBRAIC[99]);
RATES[41] = (ALGEBRAIC[96] - STATES[41])/ALGEBRAIC[102];
ALGEBRAIC[94] = (( - 
CONSTANTS[135]*ALGEBRAIC[91])/1.00000)/(1.00000+pow(CONSTANTS[109]/STATES[8], 
8.00000));
ALGEBRAIC[97] = (CONSTANTS[0]==2.00000 ?  ALGEBRAIC[94]*1.70000 : ALGEBRAIC[94]);
ALGEBRAIC[100] = CONSTANTS[128]/(1.00000+0.0123000/STATES[8]);
ALGEBRAIC[103] = (ALGEBRAIC[100]<0.00100000 ? 0.00100000 : ALGEBRAIC[100]);
RATES[42] = (ALGEBRAIC[97] - STATES[42])/ALGEBRAIC[103];
ALGEBRAIC[64] = 
(( CONSTANTS[5]*CONSTANTS[6])/( CONSTANTS[10]*CONSTANTS[7]))*log(CONSTANTS[3]/ST
ATES[5]);
ALGEBRAIC[71] = 1.00000/(1.00000+exp(((STATES[0]+CONSTANTS[45]) - 213.600)/151.200));
ALGEBRAIC[72] = 1.00000 - ALGEBRAIC[71];
ALGEBRAIC[73] =  ALGEBRAIC[71]*STATES[19]+ ALGEBRAIC[72]*STATES[20];
ALGEBRAIC[74] =  ALGEBRAIC[71]*STATES[22]+ ALGEBRAIC[72]*STATES[23];
ALGEBRAIC[75] = 1.00000/(1.00000+CONSTANTS[19]/ALGEBRAIC[49]);
ALGEBRAIC[76] =  CONSTANTS[119]*(STATES[0] - ALGEBRAIC[64])*( (1.00000 - 
ALGEBRAIC[75])*STATES[18]*ALGEBRAIC[73]+ ALGEBRAIC[75]*STATES[21]*ALGEBRAIC[74]);
ALGEBRAIC[115] =  CONSTANTS[123]* pow((CONSTANTS[3]/5.00000), 1.0 / 
2)*STATES[38]*(STATES[0] - ALGEBRAIC[64]);
ALGEBRAIC[65] = 
(( CONSTANTS[5]*CONSTANTS[6])/( CONSTANTS[10]*CONSTANTS[7]))*log((CONSTANTS[3]+ 
CONSTANTS[35]*CONSTANTS[1])/(STATES[5]+ CONSTANTS[35]*STATES[3]));
ALGEBRAIC[116] = 1.00000+0.600000/(1.00000+pow(3.80000e-05/STATES[9], 1.40000));
ALGEBRAIC[117] =  CONSTANTS[124]*ALGEBRAIC[116]*STATES[39]*STATES[40]*(STATES[0] - 
ALGEBRAIC[65]);
ALGEBRAIC[118] = 4.09400/(1.00000+exp( 0.121700*((STATES[0] - ALGEBRAIC[64]) - 
49.9340)));
ALGEBRAIC[119] = ( 15.7200*exp( 0.0674000*((STATES[0] - ALGEBRAIC[64]) - 
3.25700))+exp( 0.0618000*((STATES[0] - ALGEBRAIC[64]) - 594.310)))/(1.00000+exp( - 
0.162900*((STATES[0] - ALGEBRAIC[64])+14.2070)));
ALGEBRAIC[120] = ALGEBRAIC[118]/(ALGEBRAIC[118]+ALGEBRAIC[119]);
ALGEBRAIC[121] =  CONSTANTS[125]* pow((CONSTANTS[3]/5.00000), 1.0 / 
2)*ALGEBRAIC[120]*(STATES[0] - ALGEBRAIC[64]);
```

```
ALGEBRAIC[185] =  CONSTANTS[83]*exp(( (1.00000 - 
CONSTANTS[84])*ALGEBRAIC[28])/3.00000);
ALGEBRAIC[189] = ( CONSTANTS[78]*pow(CONSTANTS[3]/CONSTANTS[86], 
2.00000))/((pow(1.00000+CONSTANTS[1]/ALGEBRAIC[185], 
3.00000)+pow(1.00000+CONSTANTS[3]/CONSTANTS[86], 2.00000)) - 1.00000);
ALGEBRAIC[186] = 
CONSTANTS[91]/(1.00000+CONSTANTS[90]/CONSTANTS[92]+STATES[3]/CONSTANTS[93]+STA
TES[5]/CONSTANTS[94]);
ALGEBRAIC[190] = 
( CONSTANTS[79]*ALGEBRAIC[186]*CONSTANTS[90])/(1.00000+CONSTANTS[88]/CONSTANTS
[89]);
ALGEBRAIC[184] =  CONSTANTS[82]*exp(( CONSTANTS[84]*ALGEBRAIC[28])/3.00000);
ALGEBRAIC[187] = ( CONSTANTS[74]*pow(STATES[3]/ALGEBRAIC[184], 
3.00000))/((pow(1.00000+STATES[3]/ALGEBRAIC[184], 
3.00000)+pow(1.00000+STATES[5]/CONSTANTS[85], 2.00000)) - 1.00000);
ALGEBRAIC[188] = ( CONSTANTS[77]*pow(CONSTANTS[1]/ALGEBRAIC[185], 
3.00000))/((pow(1.00000+CONSTANTS[1]/ALGEBRAIC[185], 
3.00000)+pow(1.00000+CONSTANTS[3]/CONSTANTS[86], 2.00000)) - 1.00000);
ALGEBRAIC[191] = ( CONSTANTS[81]*pow(STATES[5]/CONSTANTS[85], 
2.00000))/((pow(1.00000+STATES[3]/ALGEBRAIC[184], 
3.00000)+pow(1.00000+STATES[5]/CONSTANTS[85], 2.00000)) - 1.00000);
ALGEBRAIC[192] =  CONSTANTS[161]*ALGEBRAIC[187]*CONSTANTS[160]+ 
ALGEBRAIC[188]*ALGEBRAIC[191]*ALGEBRAIC[190]+ 
CONSTANTS[160]*ALGEBRAIC[191]*ALGEBRAIC[190]+ 
ALGEBRAIC[190]*ALGEBRAIC[187]*CONSTANTS[160];
ALGEBRAIC[193] =  ALGEBRAIC[188]*CONSTANTS[159]*ALGEBRAIC[191]+ 
ALGEBRAIC[187]*CONSTANTS[160]*ALGEBRAIC[189]+ 
ALGEBRAIC[189]*CONSTANTS[159]*ALGEBRAIC[191]+ 
CONSTANTS[160]*ALGEBRAIC[189]*ALGEBRAIC[191];
ALGEBRAIC[194] =  CONSTANTS[160]*ALGEBRAIC[189]*CONSTANTS[161]+ 
ALGEBRAIC[190]*ALGEBRAIC[188]*CONSTANTS[159]+ 
ALGEBRAIC[188]*CONSTANTS[159]*CONSTANTS[161]+ 
ALGEBRAIC[189]*CONSTANTS[161]*CONSTANTS[159];
ALGEBRAIC[195] =  ALGEBRAIC[191]*ALGEBRAIC[190]*ALGEBRAIC[188]+ 
ALGEBRAIC[189]*CONSTANTS[161]*ALGEBRAIC[187]+ 
ALGEBRAIC[188]*CONSTANTS[161]*ALGEBRAIC[187]+ 
ALGEBRAIC[190]*ALGEBRAIC[188]*ALGEBRAIC[187];
ALGEBRAIC[196] = 
ALGEBRAIC[192]/(ALGEBRAIC[192]+ALGEBRAIC[193]+ALGEBRAIC[194]+ALGEBRAIC[195]);
ALGEBRAIC[197] = 
ALGEBRAIC[193]/(ALGEBRAIC[192]+ALGEBRAIC[193]+ALGEBRAIC[194]+ALGEBRAIC[195]);
ALGEBRAIC[200] =  3.00000*( ALGEBRAIC[196]*ALGEBRAIC[189] - 
ALGEBRAIC[197]*ALGEBRAIC[190]);
ALGEBRAIC[198] = 
ALGEBRAIC[194]/(ALGEBRAIC[192]+ALGEBRAIC[193]+ALGEBRAIC[194]+ALGEBRAIC[195]);
```

```
ALGEBRAIC[199] = 
ALGEBRAIC[195]/(ALGEBRAIC[192]+ALGEBRAIC[193]+ALGEBRAIC[194]+ALGEBRAIC[195]);
ALGEBRAIC[201] =  2.00000*( ALGEBRAIC[199]*CONSTANTS[159] - 
ALGEBRAIC[198]*ALGEBRAIC[187]);
ALGEBRAIC[202] =  CONSTANTS[162]*( CONSTANTS[8]*ALGEBRAIC[200]+ 
CONSTANTS[10]*ALGEBRAIC[201]);
ALGEBRAIC[203] = 1.00000/(1.00000+exp(- (STATES[0] - 10.8968)/23.9871));
ALGEBRAIC[204] =  CONSTANTS[126]*ALGEBRAIC[203]*(STATES[0] - ALGEBRAIC[64]);
ALGEBRAIC[66] = 
CONSTANTS[37]*CONSTANTS[36]*CONSTANTS[115]*CONSTANTS[116]*(STATES[0] - 
ALGEBRAIC[64]);
ALGEBRAIC[11] = (TIME>=CONSTANTS[14]&&TIME<=CONSTANTS[15]&&(TIME - 
CONSTANTS[14]) -  floor((TIME - 
CONSTANTS[14])/CONSTANTS[17])*CONSTANTS[17]<=CONSTANTS[18] ? CONSTANTS[16] : 
0.00000);
ALGEBRAIC[98] = ( 0.500000*(STATES[3]+STATES[5]+CONSTANTS[34]+ 
4.00000*STATES[9]))/1000.00;
ALGEBRAIC[105] = exp( - CONSTANTS[134]*1.00000*( pow(ALGEBRAIC[98], 1.0 / 2)/(1.00000+ 
pow(ALGEBRAIC[98], 1.0 / 2)) -  0.300000*ALGEBRAIC[98]));
ALGEBRAIC[108] = 
( 1.00000*ALGEBRAIC[25]*( ALGEBRAIC[105]*STATES[5]*exp( 1.00000*ALGEBRAIC[28]) - 
CONSTANTS[141]*CONSTANTS[3]))/(exp( 1.00000*ALGEBRAIC[28]) - 1.00000);
ALGEBRAIC[111] =  (1.00000 - CONSTANTS[54])*( (1.00000 - 
ALGEBRAIC[90])*CONSTANTS[133]*ALGEBRAIC[108]*STATES[24]*( ALGEBRAIC[77]*(1.00000 - 
STATES[33])+ STATES[29]*ALGEBRAIC[80]*STATES[33])+ 
ALGEBRAIC[90]*CONSTANTS[138]*ALGEBRAIC[108]*STATES[24]*( ALGEBRAIC[81]*(1.00000 - 
STATES[33])+ STATES[29]*ALGEBRAIC[82]*STATES[33]));
ALGEBRAIC[206] = (STATES[6] - STATES[5])/CONSTANTS[106];
RATES[5] = ( - 
(((ALGEBRAIC[76]+ALGEBRAIC[115]+ALGEBRAIC[117]+ALGEBRAIC[121]+ALGEBRAIC[204]+ALG
EBRAIC[66]+ALGEBRAIC[11]) - 
2.00000*ALGEBRAIC[202])+ALGEBRAIC[111])*CONSTANTS[136])/( CONSTANTS[7]*CONSTANT
S[142])+( ALGEBRAIC[206]*CONSTANTS[145])/CONSTANTS[142];
ALGEBRAIC[86] = exp( - CONSTANTS[134]*1.00000*( pow(ALGEBRAIC[83], 1.0 / 2)/(1.00000+ 
pow(ALGEBRAIC[83], 1.0 / 2)) -  0.300000*ALGEBRAIC[83]));
ALGEBRAIC[89] = 
( 1.00000*ALGEBRAIC[25]*( ALGEBRAIC[86]*STATES[6]*exp( 1.00000*ALGEBRAIC[28]) - 
CONSTANTS[141]*CONSTANTS[3]))/(exp( 1.00000*ALGEBRAIC[28]) - 1.00000);
ALGEBRAIC[95] =  CONSTANTS[54]*( (1.00000 - 
ALGEBRAIC[90])*CONSTANTS[133]*ALGEBRAIC[89]*STATES[24]*( ALGEBRAIC[77]*(1.00000 - 
STATES[32])+ STATES[29]*ALGEBRAIC[80]*STATES[32])+ 
ALGEBRAIC[90]*CONSTANTS[138]*ALGEBRAIC[89]*STATES[24]*( ALGEBRAIC[81]*(1.00000 - 
STATES[32])+ STATES[29]*ALGEBRAIC[82]*STATES[32]));
RATES[6] = ( - ALGEBRAIC[95]*CONSTANTS[136])/( CONSTANTS[7]*CONSTANTS[145]) - 
ALGEBRAIC[206];
```

```
ALGEBRAIC[63] =
(( CONSTANTS[5]*CONSTANTS[6])/( CONSTANTS[8]*CONSTANTS[7]))*log(CONSTANTS[1]/STATES[3]);
ALGEBRAIC[67] = 1.00000/(1.00000+CONSTANTS[19]/ALGEBRAIC[49]);
ALGEBRAIC[68] =  CONSTANTS[41]*(STATES[0] - ALGEBRAIC[63])*pow(STATES[10], 3.00000)*( (1.00000 - ALGEBRAIC[67])*STATES[11]*STATES[12]+ ALGEBRAIC[67]*STATES[13]*STATES[14]);
ALGEBRAIC[69] = 1.00000/(1.00000+CONSTANTS[19]/ALGEBRAIC[49]);
ALGEBRAIC[70] =  CONSTANTS[118]*(STATES[0] - ALGEBRAIC[63])*STATES[15]*( (1.00000 - ALGEBRAIC[69])*STATES[16]+ ALGEBRAIC[69]*STATES[17]);
ALGEBRAIC[150] = 1.00000/(1.00000+pow(CONSTANTS[72]/STATES[9], 2.00000));
ALGEBRAIC[123] = exp( CONSTANTS[70]*ALGEBRAIC[28]);
ALGEBRAIC[130] = 1.00000+
(CONSTANTS[1]/CONSTANTS[63])*(1.00000+1.00000/ALGEBRAIC[123]);
ALGEBRAIC[131] = CONSTANTS[1]/( CONSTANTS[63]*ALGEBRAIC[123]*ALGEBRAIC[130]);
ALGEBRAIC[134] =  ALGEBRAIC[131]*CONSTANTS[67];
ALGEBRAIC[124] = 1.00000+ (STATES[3]/CONSTANTS[63])*(1.00000+ALGEBRAIC[123]);
ALGEBRAIC[125] = ( STATES[3]*ALGEBRAIC[123])/( CONSTANTS[63]*ALGEBRAIC[124]);
ALGEBRAIC[137] =  ALGEBRAIC[125]*CONSTANTS[67];
ALGEBRAIC[127] = 1.00000+
(STATES[3]/CONSTANTS[61])*(1.00000+STATES[3]/CONSTANTS[62]);
ALGEBRAIC[128] =
( STATES[3]*STATES[3])/( ALGEBRAIC[127]*CONSTANTS[61]*CONSTANTS[62]);
ALGEBRAIC[140] =  ALGEBRAIC[128]*ALGEBRAIC[125]*CONSTANTS[65];
ALGEBRAIC[141] =  ALGEBRAIC[131]*CONSTANTS[147]*CONSTANTS[65];
ALGEBRAIC[132] = 1.00000/ALGEBRAIC[130];
ALGEBRAIC[133] =  ALGEBRAIC[132]*CONSTANTS[66];
ALGEBRAIC[135] = ALGEBRAIC[133]+ALGEBRAIC[134];
ALGEBRAIC[122] = exp( CONSTANTS[71]*ALGEBRAIC[28]);
ALGEBRAIC[126] = 1.00000/ALGEBRAIC[124];
ALGEBRAIC[136] = ( ALGEBRAIC[126]*CONSTANTS[66])/ALGEBRAIC[122];
ALGEBRAIC[138] = ALGEBRAIC[136]+ALGEBRAIC[137];
ALGEBRAIC[129] = 1.00000/ALGEBRAIC[127];
ALGEBRAIC[139] =  ALGEBRAIC[129]*STATES[9]*CONSTANTS[68];
ALGEBRAIC[142] =  CONSTANTS[150]*ALGEBRAIC[138]*(ALGEBRAIC[140]+ALGEBRAIC[139])+ CONSTANTS[151]*ALGEBRAIC[140]*(CONSTANTS[150]+ALGEBRAIC[135]);
ALGEBRAIC[143] =  CONSTANTS[149]*ALGEBRAIC[140]*(ALGEBRAIC[138]+CONSTANTS[151])+ ALGEBRAIC[138]*ALGEBRAIC[139]*(CONSTANTS[149]+ALGEBRAIC[141]);
ALGEBRAIC[144] =  CONSTANTS[149]*ALGEBRAIC[135]*(ALGEBRAIC[140]+ALGEBRAIC[139])+ ALGEBRAIC[141]*ALGEBRAIC[139]*(CONSTANTS[150]+ALGEBRAIC[135]);
ALGEBRAIC[145] =  CONSTANTS[150]*ALGEBRAIC[141]*(ALGEBRAIC[138]+CONSTANTS[151])+ ALGEBRAIC[135]*CONSTANTS[151]*(CONSTANTS[149]+ALGEBRAIC[141]);
ALGEBRAIC[146] =
ALGEBRAIC[142]/(ALGEBRAIC[142]+ALGEBRAIC[143]+ALGEBRAIC[144]+ALGEBRAIC[145]);
ALGEBRAIC[147] =
ALGEBRAIC[143]/(ALGEBRAIC[142]+ALGEBRAIC[143]+ALGEBRAIC[144]+ALGEBRAIC[145]);
```

```
ALGEBRAIC[148] =
ALGEBRAIC[144]/(ALGEBRAIC[142]+ALGEBRAIC[143]+ALGEBRAIC[144]+ALGEBRAIC[145]);
ALGEBRAIC[149] =
ALGEBRAIC[145]/(ALGEBRAIC[142]+ALGEBRAIC[143]+ALGEBRAIC[144]+ALGEBRAIC[145]);
ALGEBRAIC[151] = ( 3.00000*( ALGEBRAIC[149]*ALGEBRAIC[140] -
ALGEBRAIC[146]*ALGEBRAIC[141])+ ALGEBRAIC[148]*ALGEBRAIC[137]) -
ALGEBRAIC[147]*ALGEBRAIC[134];
ALGEBRAIC[152] =  ALGEBRAIC[147]*CONSTANTS[150] -  ALGEBRAIC[146]*CONSTANTS[149];
ALGEBRAIC[153] =  (1.00000 -
CONSTANTS[60])*CONSTANTS[152]*ALGEBRAIC[150]*( CONSTANTS[8]*ALGEBRAIC[151]+
CONSTANTS[9]*ALGEBRAIC[152]);
ALGEBRAIC[205] = ( CONSTANTS[97]*ALGEBRAIC[25]*( STATES[3]*exp(ALGEBRAIC[28]) -
CONSTANTS[1]))/(exp(ALGEBRAIC[28]) - 1.00000);
ALGEBRAIC[104] = exp( - CONSTANTS[134]*1.00000*( pow(ALGEBRAIC[98], 1.0 / 2)/(1.00000+
pow(ALGEBRAIC[98], 1.0 / 2)) -  0.300000*ALGEBRAIC[98]));
ALGEBRAIC[107] =
( 1.00000*ALGEBRAIC[25]*( ALGEBRAIC[104]*STATES[3]*exp( 1.00000*ALGEBRAIC[28]) -
CONSTANTS[140]*CONSTANTS[1]))/(exp( 1.00000*ALGEBRAIC[28]) - 1.00000);
ALGEBRAIC[110] =  (1.00000 - CONSTANTS[54])*( (1.00000 -
ALGEBRAIC[90])*CONSTANTS[132]*ALGEBRAIC[107]*STATES[24]*( ALGEBRAIC[77]*(1.00000 -
STATES[33])+ STATES[29]*ALGEBRAIC[80]*STATES[33])+
ALGEBRAIC[90]*CONSTANTS[137]*ALGEBRAIC[107]*STATES[24]*( ALGEBRAIC[81]*(1.00000 -
STATES[33])+ STATES[29]*ALGEBRAIC[82]*STATES[33]));
ALGEBRAIC[208] = (STATES[4] - STATES[3])/CONSTANTS[105];
RATES[3] = ( - (ALGEBRAIC[68]+ALGEBRAIC[70]+ 3.00000*ALGEBRAIC[153]+ALGEBRAIC[110]+
3.00000*ALGEBRAIC[202]+ALGEBRAIC[205])*CONSTANTS[136])/( CONSTANTS[7]*CONSTANT
S[142])+( ALGEBRAIC[208]*CONSTANTS[145])/CONSTANTS[142];
ALGEBRAIC[180] = 1.00000/(1.00000+pow(CONSTANTS[72]/STATES[2], 2.00000));
ALGEBRAIC[160] = 1.00000+
(CONSTANTS[1]/CONSTANTS[63])*(1.00000+1.00000/ALGEBRAIC[123]);
ALGEBRAIC[161] = CONSTANTS[1]/( CONSTANTS[63]*ALGEBRAIC[123]*ALGEBRAIC[160]);
ALGEBRAIC[164] =  ALGEBRAIC[161]*CONSTANTS[67];
ALGEBRAIC[154] = 1.00000+ (STATES[4]/CONSTANTS[63])*(1.00000+ALGEBRAIC[123]);
ALGEBRAIC[155] = ( STATES[4]*ALGEBRAIC[123])/( CONSTANTS[63]*ALGEBRAIC[154]);
ALGEBRAIC[167] =  ALGEBRAIC[155]*CONSTANTS[67];
ALGEBRAIC[157] = 1.00000+
(STATES[4]/CONSTANTS[61])*(1.00000+STATES[4]/CONSTANTS[62]);
ALGEBRAIC[158] =
( STATES[4]*STATES[4])/( ALGEBRAIC[157]*CONSTANTS[61]*CONSTANTS[62]);
ALGEBRAIC[170] =  ALGEBRAIC[158]*ALGEBRAIC[155]*CONSTANTS[65];
ALGEBRAIC[171] =  ALGEBRAIC[161]*CONSTANTS[154]*CONSTANTS[65];
ALGEBRAIC[162] = 1.00000/ALGEBRAIC[160];
ALGEBRAIC[163] =  ALGEBRAIC[162]*CONSTANTS[66];
ALGEBRAIC[165] = ALGEBRAIC[163]+ALGEBRAIC[164];
ALGEBRAIC[156] = 1.00000/ALGEBRAIC[154];
ALGEBRAIC[166] = ( ALGEBRAIC[156]*CONSTANTS[66])/ALGEBRAIC[122];
```

```
ALGEBRAIC[168] = ALGEBRAIC[166]+ALGEBRAIC[167];
ALGEBRAIC[159] = 1.00000/ALGEBRAIC[157];
ALGEBRAIC[169] =  ALGEBRAIC[159]*STATES[2]*CONSTANTS[68];
ALGEBRAIC[172] =  CONSTANTS[157]*ALGEBRAIC[168]*(ALGEBRAIC[170]+ALGEBRAIC[169])+
CONSTANTS[158]*ALGEBRAIC[170]*(CONSTANTS[157]+ALGEBRAIC[165]);
ALGEBRAIC[173] =  CONSTANTS[156]*ALGEBRAIC[170]*(ALGEBRAIC[168]+CONSTANTS[158])+
ALGEBRAIC[168]*ALGEBRAIC[169]*(CONSTANTS[156]+ALGEBRAIC[171]);
ALGEBRAIC[174] =  CONSTANTS[156]*ALGEBRAIC[165]*(ALGEBRAIC[170]+ALGEBRAIC[169])+
ALGEBRAIC[171]*ALGEBRAIC[169]*(CONSTANTS[157]+ALGEBRAIC[165]);
ALGEBRAIC[175] =  CONSTANTS[157]*ALGEBRAIC[171]*(ALGEBRAIC[168]+CONSTANTS[158])+
ALGEBRAIC[165]*CONSTANTS[158]*(CONSTANTS[156]+ALGEBRAIC[171]);
ALGEBRAIC[176] =
ALGEBRAIC[172]/(ALGEBRAIC[172]+ALGEBRAIC[173]+ALGEBRAIC[174]+ALGEBRAIC[175]);
ALGEBRAIC[177] =
ALGEBRAIC[173]/(ALGEBRAIC[172]+ALGEBRAIC[173]+ALGEBRAIC[174]+ALGEBRAIC[175]);
ALGEBRAIC[178] =
ALGEBRAIC[174]/(ALGEBRAIC[172]+ALGEBRAIC[173]+ALGEBRAIC[174]+ALGEBRAIC[175]);
ALGEBRAIC[179] =
ALGEBRAIC[175]/(ALGEBRAIC[172]+ALGEBRAIC[173]+ALGEBRAIC[174]+ALGEBRAIC[175]);
ALGEBRAIC[181] = ( 3.00000*( ALGEBRAIC[179]*ALGEBRAIC[170] -
ALGEBRAIC[176]*ALGEBRAIC[171])+ ALGEBRAIC[178]*ALGEBRAIC[167]) -
ALGEBRAIC[177]*ALGEBRAIC[164];
ALGEBRAIC[182] =  ALGEBRAIC[177]*CONSTANTS[157] -  ALGEBRAIC[176]*CONSTANTS[156];
ALGEBRAIC[183] =
CONSTANTS[60]*CONSTANTS[152]*ALGEBRAIC[180]*( CONSTANTS[8]*ALGEBRAIC[181]+
CONSTANTS[9]*ALGEBRAIC[182]);
ALGEBRAIC[85] = exp( - CONSTANTS[134]*1.00000*( pow(ALGEBRAIC[83], 1.0 / 2)/(1.00000+
pow(ALGEBRAIC[83], 1.0 / 2)) -  0.300000*ALGEBRAIC[83]));
ALGEBRAIC[88] =
( 1.00000*ALGEBRAIC[25]*( ALGEBRAIC[85]*STATES[4]*exp( 1.00000*ALGEBRAIC[28]) -
CONSTANTS[140]*CONSTANTS[1]))/(exp( 1.00000*ALGEBRAIC[28]) - 1.00000);
ALGEBRAIC[92] =  CONSTANTS[54]*( (1.00000 -
ALGEBRAIC[90])*CONSTANTS[132]*ALGEBRAIC[88]*STATES[24]*( ALGEBRAIC[77]*(1.00000 -
STATES[32])+ STATES[29]*ALGEBRAIC[80]*STATES[32])+
ALGEBRAIC[90]*CONSTANTS[137]*ALGEBRAIC[88]*STATES[24]*( ALGEBRAIC[81]*(1.00000 -
STATES[32])+ STATES[29]*ALGEBRAIC[82]*STATES[32]));
RATES[4] = ( - (ALGEBRAIC[92]+
3.00000*ALGEBRAIC[183])*CONSTANTS[136])/( CONSTANTS[7]*CONSTANTS[145]) -
ALGEBRAIC[208];
ALGEBRAIC[211] = (STATES[2] - STATES[9])/CONSTANTS[107];
ALGEBRAIC[213] = 1.00000/(1.00000+CONSTANTS[19]/ALGEBRAIC[49]);
ALGEBRAIC[215] =  CONSTANTS[110]*( (1.00000 - ALGEBRAIC[213])*STATES[41]+
ALGEBRAIC[213]*STATES[42]);
ALGEBRAIC[56] =
1.00000/(1.00000+( CONSTANTS[28]*CONSTANTS[29])/pow(CONSTANTS[29]+STATES[2],
2.00000)+( CONSTANTS[30]*CONSTANTS[31])/pow(CONSTANTS[31]+STATES[2], 2.00000));
```

```
RATES[2] =  ALGEBRAIC[56]*((( - (ALGEBRAIC[91] - 
2.00000*ALGEBRAIC[183])*CONSTANTS[136])/( 2.00000*CONSTANTS[7]*CONSTANTS[145])+(
ALGEBRAIC[215]*CONSTANTS[144])/CONSTANTS[145]) - ALGEBRAIC[211]);
ALGEBRAIC[101] = exp( - CONSTANTS[134]*4.00000*( pow(ALGEBRAIC[98], 1.0 / 2)/(1.00000+
pow(ALGEBRAIC[98], 1.0 / 2)) -  0.300000*ALGEBRAIC[98]));
ALGEBRAIC[106] = 
( 4.00000*ALGEBRAIC[25]*( ALGEBRAIC[101]*STATES[9]*exp( 2.00000*ALGEBRAIC[28]) - 
CONSTANTS[139]*CONSTANTS[2]))/(exp( 2.00000*ALGEBRAIC[28]) - 1.00000);
ALGEBRAIC[109] =  (1.00000 - CONSTANTS[54])*( (1.00000 - 
ALGEBRAIC[90])*CONSTANTS[121]*ALGEBRAIC[106]*STATES[24]*( ALGEBRAIC[77]*(1.00000 - 
STATES[33])+ STATES[29]*ALGEBRAIC[80]*STATES[33])+ 
ALGEBRAIC[90]*CONSTANTS[131]*ALGEBRAIC[106]*STATES[24]*( ALGEBRAIC[81]*(1.00000 - 
STATES[33])+ STATES[29]*ALGEBRAIC[82]*STATES[33]));
ALGEBRAIC[112] = ALGEBRAIC[91]+ALGEBRAIC[109];
ALGEBRAIC[113] = ALGEBRAIC[92]+ALGEBRAIC[110];
ALGEBRAIC[114] = ALGEBRAIC[95]+ALGEBRAIC[111];
ALGEBRAIC[209] = ( CONSTANTS[99]*STATES[9])/(CONSTANTS[100]+STATES[9]);
ALGEBRAIC[207] = 
( CONSTANTS[98]*4.00000*ALGEBRAIC[25]*( ALGEBRAIC[101]*STATES[9]*exp( 2.00000*ALG
EBRAIC[28]) -  CONSTANTS[139]*CONSTANTS[2]))/(exp( 2.00000*ALGEBRAIC[28]) - 1.00000);
ALGEBRAIC[210] = 
(( CONSTANTS[104]*CONSTANTS[101])/(1.00000+CONSTANTS[103]/STATES[2]))*(STATES[0] - 
CONSTANTS[114]);
ALGEBRAIC[212] =  (( (1.00000 - 
CONSTANTS[104])*CONSTANTS[101])/(1.00000+CONSTANTS[103]/STATES[9]))*(STATES[0] - 
CONSTANTS[114]);
ALGEBRAIC[214] = ALGEBRAIC[210]+ALGEBRAIC[212];
ALGEBRAIC[216] =  CONSTANTS[102]*(STATES[0] - CONSTANTS[114]);
RATES[0] = - 
(ALGEBRAIC[68]+ALGEBRAIC[70]+ALGEBRAIC[76]+ALGEBRAIC[112]+ALGEBRAIC[113]+ALGEBR
AIC[114]+ALGEBRAIC[115]+ALGEBRAIC[117]+ALGEBRAIC[121]+ALGEBRAIC[153]+ALGEBRAIC[1
83]+ALGEBRAIC[202]+ALGEBRAIC[205]+ALGEBRAIC[204]+ALGEBRAIC[209]+ALGEBRAIC[207]+
ALGEBRAIC[214]+ALGEBRAIC[216]+ALGEBRAIC[66]+ALGEBRAIC[11]);
ALGEBRAIC[217] = ( CONSTANTS[129]*0.00542500*STATES[9])/(STATES[9]+0.000920000);
ALGEBRAIC[218] = 
( CONSTANTS[129]*2.75000*0.00542500*STATES[9])/((STATES[9]+0.000920000) - 
0.000170000);
ALGEBRAIC[219] = 1.00000/(1.00000+CONSTANTS[19]/ALGEBRAIC[49]);
ALGEBRAIC[220] = ( 0.00488250*STATES[7])/15.0000;
ALGEBRAIC[221] =  CONSTANTS[111]*(( (1.00000 - ALGEBRAIC[219])*ALGEBRAIC[217]+ 
ALGEBRAIC[219]*ALGEBRAIC[218]) - ALGEBRAIC[220]);
ALGEBRAIC[53] = 
1.00000/(1.00000+( CONSTANTS[113]*CONSTANTS[25])/pow(CONSTANTS[25]+STATES[9], 
2.00000)+( CONSTANTS[26]*CONSTANTS[27])/pow(CONSTANTS[27]+STATES[9], 2.00000));
RATES[9] =  ALGEBRAIC[53]*((( - ((ALGEBRAIC[109]+ALGEBRAIC[209]+ALGEBRAIC[207]) - 
2.00000*ALGEBRAIC[153])*CONSTANTS[136])/( 2.00000*CONSTANTS[7]*CONSTANTS[142]) -
```

```
( ALGEBRAIC[221]*CONSTANTS[143])/CONSTANTS[142])+( ALGEBRAIC[211]*CONSTANTS[145]
)/CONSTANTS[142]);
ALGEBRAIC[222] = (STATES[7] - STATES[8])/60.0000;
RATES[7] = ALGEBRAIC[221] - ( ALGEBRAIC[222]*CONSTANTS[144])/CONSTANTS[143];
ALGEBRAIC[59] =
1.00000/(1.00000+( CONSTANTS[32]*CONSTANTS[33])/pow(CONSTANTS[33]+STATES[8],
2.00000));
RATES[8] =  ALGEBRAIC[59]*(ALGEBRAIC[222] - ALGEBRAIC[215]);
}

void CLASS_NAME::computeVariables()
{
ALGEBRAIC[3] = 1.00000/(1.00000+exp((STATES[0]+87.6100)/7.48800));
ALGEBRAIC[4] = 1.00000/(1.00000+exp((STATES[0]+93.8100)/7.48800));
ALGEBRAIC[9] = 1.00000/(1.00000+exp((STATES[0]+18.0800)/2.79160));
ALGEBRAIC[0] = 1.00000/pow(1.00000+exp(- (STATES[0]+56.8600)/9.03000), 2.00000);
ALGEBRAIC[13] =  0.129200*exp(- pow((STATES[0]+45.7900)/15.5400, 2.00000))+
0.0648700*exp(- pow((STATES[0] - 4.82300)/51.1200, 2.00000));
ALGEBRAIC[2] = 1.00000/(1.00000+exp(- (STATES[0]+42.8500)/5.26400));
ALGEBRAIC[16] =  0.129200*exp(- pow((STATES[0]+45.7900)/15.5400, 2.00000))+
0.0648700*exp(- pow((STATES[0] - 4.82300)/51.1200, 2.00000));
ALGEBRAIC[5] = 1.00000/(1.00000+exp(- ((STATES[0]+CONSTANTS[45]) - 14.3400)/14.8200));
ALGEBRAIC[17] = 1.05150/(1.00000/( 1.20890*(1.00000+exp(- ((STATES[0]+CONSTANTS[45]) -
18.4099)/29.3814)))+3.50000/(1.00000+exp((STATES[0]+CONSTANTS[45]+100.000)/29.3814))
);
ALGEBRAIC[7] = (STATES[0]>=31.4978 ? 1.00000 :  1.07630*exp( - 1.00700*exp( -
0.0829000*STATES[0])));
ALGEBRAIC[22] = CONSTANTS[52]+0.600000+1.00000/(exp( -
0.0500000*(STATES[0]+CONSTANTS[51]+6.00000))+exp( 0.0900000*(STATES[0]+CONSTANTS[
51]+14.0000)));
ALGEBRAIC[8] = 1.00000/(1.00000+exp((STATES[0]+19.5800)/3.69600));
ALGEBRAIC[23] = 7.00000+1.00000/( 0.00450000*exp(- (STATES[0]+20.0000)/10.0000)+
0.00450000*exp((STATES[0]+20.0000)/10.0000));
ALGEBRAIC[24] = 1000.00+1.00000/( 3.50000e-05*exp(- (STATES[0]+5.00000)/4.00000)+
3.50000e-05*exp((STATES[0]+5.00000)/6.00000));
ALGEBRAIC[10] =  STATES[29]*1.00000;
ALGEBRAIC[20] =
1.00000/(CONSTANTS[47]/ALGEBRAIC[10]+pow(1.00000+CONSTANTS[46]/STATES[2],
4.00000));
ALGEBRAIC[21] =
1.00000/(CONSTANTS[47]/ALGEBRAIC[10]+pow(1.00000+CONSTANTS[46]/STATES[9],
4.00000));
ALGEBRAIC[12] = 1.00000/(1.00000+exp(- (STATES[0]+11.6000)/8.93200));
ALGEBRAIC[27] = 817.300+1.00000/( 0.000232600*exp((STATES[0]+48.2800)/17.8000)+
0.00129200*exp(- (STATES[0]+210.000)/230.000));
ALGEBRAIC[32] = 1.00000/(1.00000+exp(- ((STATES[0]+CONSTANTS[45]) - 24.3400)/14.8200));
```

```
ALGEBRAIC[19] = ALGEBRAIC[8];
ALGEBRAIC[33] = 7.00000+1.00000/( 0.0400000*exp(- (STATES[0] - 4.00000)/7.00000)+ 0.0400000*exp((STATES[0] - 4.00000)/7.00000));
ALGEBRAIC[34] = 100.000+1.00000/( 0.000120000*exp(- STATES[0]/3.00000)+ 0.000120000*exp(STATES[0]/7.00000));
ALGEBRAIC[35] =  2.50000*ALGEBRAIC[23];
ALGEBRAIC[26] = ALGEBRAIC[12];
ALGEBRAIC[36] = 1.00000/( 0.0100000*exp((STATES[0] - 50.0000)/20.0000)+ 0.0193000*exp(- (STATES[0]+66.5400)/31.0000));
ALGEBRAIC[43] = ( CONSTANTS[22]*(1.00000 - STATES[1]))/(1.00000+CONSTANTS[23]/STATES[2]);
ALGEBRAIC[1] = 1.00000/pow(1.00000+exp((STATES[0]+71.5500)/7.43000), 2.00000);
ALGEBRAIC[14] = (STATES[0]>=- 40.0000 ? 0.00000 :  0.0570000*exp(- (STATES[0]+80.0000)/6.80000));
ALGEBRAIC[29] = (STATES[0]>=- 40.0000 ? 0.770000/( 0.130000*(1.00000+exp(- (STATES[0]+10.6600)/11.1000))) :  2.70000*exp( 0.0790000*STATES[0])+ 310000.*exp( 0.348500*STATES[0]));
ALGEBRAIC[37] = 1.00000/(ALGEBRAIC[14]+ALGEBRAIC[29]);
ALGEBRAIC[40] =  2.50000*ALGEBRAIC[33];
ALGEBRAIC[38] = ALGEBRAIC[1];
ALGEBRAIC[15] = (STATES[0]>=- 40.0000 ? 0.00000 : ( ( - 25428.0*exp( 0.244400*STATES[0]) - 6.94800e-06*exp( - 0.0439100*STATES[0]))*(STATES[0]+37.7800))/(1.00000+exp( 0.311000*(STATES[0]+79.2300) )));
ALGEBRAIC[30] = (STATES[0]>=- 40.0000 ? ( 0.600000*exp( 0.0570000*STATES[0]))/(1.00000+exp( - 0.100000*(STATES[0]+32.0000))) : ( 0.0242400*exp( - 0.0105200*STATES[0]))/(1.00000+exp( - 0.137800*(STATES[0]+40.1400))));
ALGEBRAIC[44] = 1.00000/(ALGEBRAIC[15]+ALGEBRAIC[30]);
ALGEBRAIC[45] = 1.00000/pow(1.00000+exp((STATES[0]+77.5500)/7.43000), 2.00000);
ALGEBRAIC[6] = 1.00000/(1.00000+exp((STATES[0]+CONSTANTS[45]+43.9400)/5.71100));
ALGEBRAIC[18] = (CONSTANTS[0]==1.00000 ? 1.00000 - 0.950000/(1.00000+exp((STATES[0]+CONSTANTS[45]+70.0000)/5.00000)) : 1.00000);
ALGEBRAIC[31] = 4.56200+1.00000/( 0.393300*exp(- (STATES[0]+CONSTANTS[45]+100.000)/100.000)+ 0.0800400*exp((STATES[0]+CONSTANTS[45]+50.0000)/16.5900));
ALGEBRAIC[46] =  ALGEBRAIC[31]*ALGEBRAIC[18];
ALGEBRAIC[28] = ( STATES[0]*CONSTANTS[7])/( CONSTANTS[5]*CONSTANTS[6]);
ALGEBRAIC[41] =  0.116100*exp( 0.299000*ALGEBRAIC[28]);
ALGEBRAIC[47] =  0.244200*exp( - 1.60400*ALGEBRAIC[28]);
ALGEBRAIC[50] =  1.46000*ALGEBRAIC[44];
ALGEBRAIC[39] = 23.6200+1.00000/( 0.00141600*exp(- (STATES[0]+CONSTANTS[45]+96.5200)/59.0500)+ 1.78000e-08*exp((STATES[0]+CONSTANTS[45]+114.100)/8.07900));
ALGEBRAIC[51] =  ALGEBRAIC[39]*ALGEBRAIC[18];
ALGEBRAIC[42] =  0.0578000*exp( 0.971000*ALGEBRAIC[28]);
```

```
ALGEBRAIC[48] =  0.000349000*exp( - 1.06200*ALGEBRAIC[28]);
ALGEBRAIC[52] =  0.253300*exp( 0.595300*ALGEBRAIC[28]);
ALGEBRAIC[55] =  0.0652500*exp( - 0.820900*ALGEBRAIC[28]);
ALGEBRAIC[54] = 1.35400+0.000100000/(exp(((STATES[0]+CONSTANTS[45]) - 
167.400)/15.8900)+exp(- ((STATES[0]+CONSTANTS[45]) - 12.2300)/0.215400));
ALGEBRAIC[57] = 1.00000 - 
0.500000/(1.00000+exp((STATES[0]+CONSTANTS[45]+70.0000)/20.0000));
ALGEBRAIC[60] =  ALGEBRAIC[54]*ALGEBRAIC[57]*ALGEBRAIC[46];
ALGEBRAIC[61] =  ALGEBRAIC[54]*ALGEBRAIC[57]*ALGEBRAIC[51];
ALGEBRAIC[58] =  5.20000e-05*exp( 1.52500*ALGEBRAIC[28]);
ALGEBRAIC[62] = 
( ALGEBRAIC[48]*ALGEBRAIC[55]*ALGEBRAIC[58])/( ALGEBRAIC[42]*ALGEBRAIC[52]);
ALGEBRAIC[77] =  CONSTANTS[49]*STATES[25]+ CONSTANTS[120]*STATES[26];
ALGEBRAIC[78] = 0.300000+0.600000/(1.00000+exp((STATES[0] - 10.0000)/10.0000));
ALGEBRAIC[79] = 1.00000 - ALGEBRAIC[78];
ALGEBRAIC[80] =  ALGEBRAIC[78]*STATES[27]+ ALGEBRAIC[79]*STATES[28];
ALGEBRAIC[81] =  CONSTANTS[49]*STATES[30]+ CONSTANTS[120]*STATES[26];
ALGEBRAIC[82] =  ALGEBRAIC[78]*STATES[31]+ ALGEBRAIC[79]*STATES[28];
ALGEBRAIC[25] = 
( STATES[0]*CONSTANTS[7]*CONSTANTS[7])/( CONSTANTS[5]*CONSTANTS[6]);
ALGEBRAIC[83] = ( 0.500000*(STATES[4]+STATES[6]+CONSTANTS[34]+ 
4.00000*STATES[2]))/1000.00;
ALGEBRAIC[84] = exp( - CONSTANTS[134]*4.00000*( pow(ALGEBRAIC[83], 1.0 / 2)/(1.00000+ 
pow(ALGEBRAIC[83], 1.0 / 2)) -  0.300000*ALGEBRAIC[83]));
ALGEBRAIC[87] = 
( 4.00000*ALGEBRAIC[25]*( ALGEBRAIC[84]*STATES[2]*exp( 2.00000*ALGEBRAIC[28]) - 
CONSTANTS[139]*CONSTANTS[2]))/(exp( 2.00000*ALGEBRAIC[28]) - 1.00000);
ALGEBRAIC[49] = ALGEBRAIC[43]+STATES[1];
ALGEBRAIC[90] = 1.00000/(1.00000+CONSTANTS[19]/ALGEBRAIC[49]);
ALGEBRAIC[91] =  CONSTANTS[54]*( (1.00000 - 
ALGEBRAIC[90])*CONSTANTS[121]*ALGEBRAIC[87]*STATES[24]*( ALGEBRAIC[77]*(1.00000 - 
STATES[32])+ STATES[29]*ALGEBRAIC[80]*STATES[32])+ 
ALGEBRAIC[90]*CONSTANTS[131]*ALGEBRAIC[87]*STATES[24]*( ALGEBRAIC[81]*(1.00000 - 
STATES[32])+ STATES[29]*ALGEBRAIC[82]*STATES[32]));
ALGEBRAIC[93] = (( - 
CONSTANTS[127]*ALGEBRAIC[91])/1.00000)/(1.00000+pow(CONSTANTS[109]/STATES[8], 
8.00000));
ALGEBRAIC[96] = (CONSTANTS[0]==2.00000 ?  ALGEBRAIC[93]*1.70000 : ALGEBRAIC[93]);
ALGEBRAIC[99] = CONSTANTS[108]/(1.00000+0.0123000/STATES[8]);
ALGEBRAIC[102] = (ALGEBRAIC[99]<0.00100000 ? 0.00100000 : ALGEBRAIC[99]);
ALGEBRAIC[94] = (( - 
CONSTANTS[135]*ALGEBRAIC[91])/1.00000)/(1.00000+pow(CONSTANTS[109]/STATES[8], 
8.00000));
ALGEBRAIC[97] = (CONSTANTS[0]==2.00000 ?  ALGEBRAIC[94]*1.70000 : ALGEBRAIC[94]);
ALGEBRAIC[100] = CONSTANTS[128]/(1.00000+0.0123000/STATES[8]);
ALGEBRAIC[103] = (ALGEBRAIC[100]<0.00100000 ? 0.00100000 : ALGEBRAIC[100]);
```

```
ALGEBRAIC[64] =
(( CONSTANTS[5]*CONSTANTS[6])/( CONSTANTS[10]*CONSTANTS[7]))*log(CONSTANTS[3]/STATES[5]);
ALGEBRAIC[71] = 1.00000/(1.00000+exp(((STATES[0]+CONSTANTS[45]) - 213.600)/151.200));
ALGEBRAIC[72] = 1.00000 - ALGEBRAIC[71];
ALGEBRAIC[73] =  ALGEBRAIC[71]*STATES[19]+ ALGEBRAIC[72]*STATES[20];
ALGEBRAIC[74] =  ALGEBRAIC[71]*STATES[22]+ ALGEBRAIC[72]*STATES[23];
ALGEBRAIC[75] = 1.00000/(1.00000+CONSTANTS[19]/ALGEBRAIC[49]);
ALGEBRAIC[76] =  CONSTANTS[119]*(STATES[0] - ALGEBRAIC[64])*( (1.00000 -
ALGEBRAIC[75])*STATES[18]*ALGEBRAIC[73]+ ALGEBRAIC[75]*STATES[21]*ALGEBRAIC[74]);
ALGEBRAIC[115] =  CONSTANTS[123]* pow((CONSTANTS[3]/5.00000), 1.0 /
2)*STATES[38]*(STATES[0] - ALGEBRAIC[64]);
ALGEBRAIC[65] =
(( CONSTANTS[5]*CONSTANTS[6])/( CONSTANTS[10]*CONSTANTS[7]))*log((CONSTANTS[3]+
CONSTANTS[35]*CONSTANTS[1])/(STATES[5]+ CONSTANTS[35]*STATES[3]));
ALGEBRAIC[116] = 1.00000+0.600000/(1.00000+pow(3.80000e-05/STATES[9], 1.40000));
ALGEBRAIC[117] =  CONSTANTS[124]*ALGEBRAIC[116]*STATES[39]*STATES[40]*(STATES[0] -
ALGEBRAIC[65]);
ALGEBRAIC[118] = 4.09400/(1.00000+exp( 0.121700*((STATES[0] - ALGEBRAIC[64]) -
49.9340)));
ALGEBRAIC[119] = ( 15.7200*exp( 0.0674000*((STATES[0] - ALGEBRAIC[64]) -
3.25700))+exp( 0.0618000*((STATES[0] - ALGEBRAIC[64]) - 594.310)))/(1.00000+exp( -
0.162900*((STATES[0] - ALGEBRAIC[64])+14.2070)));
ALGEBRAIC[120] = ALGEBRAIC[118]/(ALGEBRAIC[118]+ALGEBRAIC[119]);
ALGEBRAIC[121] =  CONSTANTS[125]* pow((CONSTANTS[3]/5.00000), 1.0 /
2)*ALGEBRAIC[120]*(STATES[0] - ALGEBRAIC[64]);
ALGEBRAIC[185] =  CONSTANTS[83]*exp(( (1.00000 -
CONSTANTS[84])*ALGEBRAIC[28])/3.00000);
ALGEBRAIC[189] = ( CONSTANTS[78]*pow(CONSTANTS[3]/CONSTANTS[86],
2.00000))/((pow(1.00000+CONSTANTS[1]/ALGEBRAIC[185],
3.00000)+pow(1.00000+CONSTANTS[3]/CONSTANTS[86], 2.00000)) - 1.00000);
ALGEBRAIC[186] =
CONSTANTS[91]/(1.00000+CONSTANTS[90]/CONSTANTS[92]+STATES[3]/CONSTANTS[93]+STATES[5]/CONSTANTS[94]);
ALGEBRAIC[190] =
( CONSTANTS[79]*ALGEBRAIC[186]*CONSTANTS[90])/(1.00000+CONSTANTS[88]/CONSTANTS[89]);
ALGEBRAIC[184] =  CONSTANTS[82]*exp(( CONSTANTS[84]*ALGEBRAIC[28])/3.00000);
ALGEBRAIC[187] = ( CONSTANTS[74]*pow(STATES[3]/ALGEBRAIC[184],
3.00000))/((pow(1.00000+STATES[3]/ALGEBRAIC[184],
3.00000)+pow(1.00000+STATES[5]/CONSTANTS[85], 2.00000)) - 1.00000);
ALGEBRAIC[188] = ( CONSTANTS[77]*pow(CONSTANTS[1]/ALGEBRAIC[185],
3.00000))/((pow(1.00000+CONSTANTS[1]/ALGEBRAIC[185],
3.00000)+pow(1.00000+CONSTANTS[3]/CONSTANTS[86], 2.00000)) - 1.00000);
```

```
ALGEBRAIC[191] = ( CONSTANTS[81]*pow(STATES[5]/CONSTANTS[85], 
2.00000))/((pow(1.00000+STATES[3]/ALGEBRAIC[184], 
3.00000)+pow(1.00000+STATES[5]/CONSTANTS[85], 2.00000)) - 1.00000);
ALGEBRAIC[192] =  CONSTANTS[161]*ALGEBRAIC[187]*CONSTANTS[160]+
ALGEBRAIC[188]*ALGEBRAIC[191]*ALGEBRAIC[190]+
CONSTANTS[160]*ALGEBRAIC[191]*ALGEBRAIC[190]+
ALGEBRAIC[190]*ALGEBRAIC[187]*CONSTANTS[160];
ALGEBRAIC[193] =  ALGEBRAIC[188]*CONSTANTS[159]*ALGEBRAIC[191]+
ALGEBRAIC[187]*CONSTANTS[160]*ALGEBRAIC[189]+
ALGEBRAIC[189]*CONSTANTS[159]*ALGEBRAIC[191]+
CONSTANTS[160]*ALGEBRAIC[189]*ALGEBRAIC[191];
ALGEBRAIC[194] =  CONSTANTS[160]*ALGEBRAIC[189]*CONSTANTS[161]+
ALGEBRAIC[190]*ALGEBRAIC[188]*CONSTANTS[159]+
ALGEBRAIC[188]*CONSTANTS[159]*CONSTANTS[161]+
ALGEBRAIC[189]*CONSTANTS[161]*CONSTANTS[159];
ALGEBRAIC[195] =  ALGEBRAIC[191]*ALGEBRAIC[190]*ALGEBRAIC[188]+
ALGEBRAIC[189]*CONSTANTS[161]*ALGEBRAIC[187]+
ALGEBRAIC[188]*CONSTANTS[161]*ALGEBRAIC[187]+
ALGEBRAIC[190]*ALGEBRAIC[188]*ALGEBRAIC[187];
ALGEBRAIC[196] = 
ALGEBRAIC[192]/(ALGEBRAIC[192]+ALGEBRAIC[193]+ALGEBRAIC[194]+ALGEBRAIC[195]);
ALGEBRAIC[197] = 
ALGEBRAIC[193]/(ALGEBRAIC[192]+ALGEBRAIC[193]+ALGEBRAIC[194]+ALGEBRAIC[195]);
ALGEBRAIC[200] =  3.00000*( ALGEBRAIC[196]*ALGEBRAIC[189] - 
ALGEBRAIC[197]*ALGEBRAIC[190]);
ALGEBRAIC[198] = 
ALGEBRAIC[194]/(ALGEBRAIC[192]+ALGEBRAIC[193]+ALGEBRAIC[194]+ALGEBRAIC[195]);
ALGEBRAIC[199] = 
ALGEBRAIC[195]/(ALGEBRAIC[192]+ALGEBRAIC[193]+ALGEBRAIC[194]+ALGEBRAIC[195]);
ALGEBRAIC[201] =  2.00000*( ALGEBRAIC[199]*CONSTANTS[159] - 
ALGEBRAIC[198]*ALGEBRAIC[187]);
ALGEBRAIC[202] =  CONSTANTS[162]*( CONSTANTS[8]*ALGEBRAIC[200]+
CONSTANTS[10]*ALGEBRAIC[201]);
ALGEBRAIC[203] = 1.00000/(1.00000+exp(- (STATES[0] - 10.8968)/23.9871));
ALGEBRAIC[204] =  CONSTANTS[126]*ALGEBRAIC[203]*(STATES[0] - ALGEBRAIC[64]);
ALGEBRAIC[66] = 
CONSTANTS[37]*CONSTANTS[36]*CONSTANTS[115]*CONSTANTS[116]*(STATES[0] - 
ALGEBRAIC[64]);
ALGEBRAIC[11] = (TIME>=CONSTANTS[14]&&TIME<=CONSTANTS[15]&&(TIME - 
CONSTANTS[14]) -  floor((TIME - 
CONSTANTS[14])/CONSTANTS[17])*CONSTANTS[17]<=CONSTANTS[18] ? CONSTANTS[16] : 
0.00000);
ALGEBRAIC[98] = ( 0.500000*(STATES[3]+STATES[5]+CONSTANTS[34]+
4.00000*STATES[9]))/1000.00;
ALGEBRAIC[105] = exp( - CONSTANTS[134]*1.00000*( pow(ALGEBRAIC[98], 1.0 / 2)/(1.00000+
pow(ALGEBRAIC[98], 1.0 / 2)) -  0.300000*ALGEBRAIC[98]));
```

```
ALGEBRAIC[108] =
( 1.00000*ALGEBRAIC[25]*( ALGEBRAIC[105]*STATES[5]*exp( 1.00000*ALGEBRAIC[28]) -
CONSTANTS[141]*CONSTANTS[3]))/(exp( 1.00000*ALGEBRAIC[28]) - 1.00000);
ALGEBRAIC[111] =  (1.00000 - CONSTANTS[54])*( (1.00000 -
ALGEBRAIC[90])*CONSTANTS[133]*ALGEBRAIC[108]*STATES[24]*( ALGEBRAIC[77]*(1.00000 -
STATES[33])+ STATES[29]*ALGEBRAIC[80]*STATES[33])+
ALGEBRAIC[90]*CONSTANTS[138]*ALGEBRAIC[108]*STATES[24]*( ALGEBRAIC[81]*(1.00000 -
STATES[33])+ STATES[29]*ALGEBRAIC[82]*STATES[33]));
ALGEBRAIC[206] = (STATES[6] - STATES[5])/CONSTANTS[106];
ALGEBRAIC[86] = exp( - CONSTANTS[134]*1.00000*( pow(ALGEBRAIC[83], 1.0 / 2)/(1.00000+
pow(ALGEBRAIC[83], 1.0 / 2)) -  0.300000*ALGEBRAIC[83]));
ALGEBRAIC[89] =
( 1.00000*ALGEBRAIC[25]*( ALGEBRAIC[86]*STATES[6]*exp( 1.00000*ALGEBRAIC[28]) -
CONSTANTS[141]*CONSTANTS[3]))/(exp( 1.00000*ALGEBRAIC[28]) - 1.00000);
ALGEBRAIC[95] =  CONSTANTS[54]*( (1.00000 -
ALGEBRAIC[90])*CONSTANTS[133]*ALGEBRAIC[89]*STATES[24]*( ALGEBRAIC[77]*(1.00000 -
STATES[32])+ STATES[29]*ALGEBRAIC[80]*STATES[32])+
ALGEBRAIC[90]*CONSTANTS[138]*ALGEBRAIC[89]*STATES[24]*( ALGEBRAIC[81]*(1.00000 -
STATES[32])+ STATES[29]*ALGEBRAIC[82]*STATES[32]));
ALGEBRAIC[63] =
(( CONSTANTS[5]*CONSTANTS[6])/( CONSTANTS[8]*CONSTANTS[7]))*log(CONSTANTS[1]/STA
TES[3]);
ALGEBRAIC[67] = 1.00000/(1.00000+CONSTANTS[19]/ALGEBRAIC[49]);
ALGEBRAIC[68] =  CONSTANTS[41]*(STATES[0] - ALGEBRAIC[63])*pow(STATES[10],
3.00000)*( (1.00000 - ALGEBRAIC[67])*STATES[11]*STATES[12]+
ALGEBRAIC[67]*STATES[13]*STATES[14]);
ALGEBRAIC[69] = 1.00000/(1.00000+CONSTANTS[19]/ALGEBRAIC[49]);
ALGEBRAIC[70] =  CONSTANTS[118]*(STATES[0] - ALGEBRAIC[63])*STATES[15]*( (1.00000 -
ALGEBRAIC[69])*STATES[16]+ ALGEBRAIC[69]*STATES[17]);
ALGEBRAIC[150] = 1.00000/(1.00000+pow(CONSTANTS[72]/STATES[9], 2.00000));
ALGEBRAIC[123] = exp( CONSTANTS[70]*ALGEBRAIC[28]);
ALGEBRAIC[130] = 1.00000+
(CONSTANTS[1]/CONSTANTS[63])*(1.00000+1.00000/ALGEBRAIC[123]);
ALGEBRAIC[131] = CONSTANTS[1]/( CONSTANTS[63]*ALGEBRAIC[123]*ALGEBRAIC[130]);
ALGEBRAIC[134] =  ALGEBRAIC[131]*CONSTANTS[67];
ALGEBRAIC[124] = 1.00000+ (STATES[3]/CONSTANTS[63])*(1.00000+ALGEBRAIC[123]);
ALGEBRAIC[125] = ( STATES[3]*ALGEBRAIC[123])/( CONSTANTS[63]*ALGEBRAIC[124]);
ALGEBRAIC[137] =  ALGEBRAIC[125]*CONSTANTS[67];
ALGEBRAIC[127] = 1.00000+
(STATES[3]/CONSTANTS[61])*(1.00000+STATES[3]/CONSTANTS[62]);
ALGEBRAIC[128] =
( STATES[3]*STATES[3])/( ALGEBRAIC[127]*CONSTANTS[61]*CONSTANTS[62]);
ALGEBRAIC[140] =  ALGEBRAIC[128]*ALGEBRAIC[125]*CONSTANTS[65];
ALGEBRAIC[141] =  ALGEBRAIC[131]*CONSTANTS[147]*CONSTANTS[65];
ALGEBRAIC[132] = 1.00000/ALGEBRAIC[130];
ALGEBRAIC[133] =  ALGEBRAIC[132]*CONSTANTS[66];
```

```
ALGEBRAIC[135] = ALGEBRAIC[133]+ALGEBRAIC[134];
ALGEBRAIC[122] = exp( CONSTANTS[71]*ALGEBRAIC[28]);
ALGEBRAIC[126] = 1.00000/ALGEBRAIC[124];
ALGEBRAIC[136] = ( ALGEBRAIC[126]*CONSTANTS[66])/ALGEBRAIC[122];
ALGEBRAIC[138] = ALGEBRAIC[136]+ALGEBRAIC[137];
ALGEBRAIC[129] = 1.00000/ALGEBRAIC[127];
ALGEBRAIC[139] =  ALGEBRAIC[129]*STATES[9]*CONSTANTS[68];
ALGEBRAIC[142] =  CONSTANTS[150]*ALGEBRAIC[138]*(ALGEBRAIC[140]+ALGEBRAIC[139])+
CONSTANTS[151]*ALGEBRAIC[140]*(CONSTANTS[150]+ALGEBRAIC[135]);
ALGEBRAIC[143] =  CONSTANTS[149]*ALGEBRAIC[140]*(ALGEBRAIC[138]+CONSTANTS[151])+
ALGEBRAIC[138]*ALGEBRAIC[139]*(CONSTANTS[149]+ALGEBRAIC[141]);
ALGEBRAIC[144] =  CONSTANTS[149]*ALGEBRAIC[135]*(ALGEBRAIC[140]+ALGEBRAIC[139])+
ALGEBRAIC[141]*ALGEBRAIC[139]*(CONSTANTS[150]+ALGEBRAIC[135]);
ALGEBRAIC[145] =  CONSTANTS[150]*ALGEBRAIC[141]*(ALGEBRAIC[138]+CONSTANTS[151])+
ALGEBRAIC[135]*CONSTANTS[151]*(CONSTANTS[149]+ALGEBRAIC[141]);
ALGEBRAIC[146] =
ALGEBRAIC[142]/(ALGEBRAIC[142]+ALGEBRAIC[143]+ALGEBRAIC[144]+ALGEBRAIC[145]);
ALGEBRAIC[147] =
ALGEBRAIC[143]/(ALGEBRAIC[142]+ALGEBRAIC[143]+ALGEBRAIC[144]+ALGEBRAIC[145]);
ALGEBRAIC[148] =
ALGEBRAIC[144]/(ALGEBRAIC[142]+ALGEBRAIC[143]+ALGEBRAIC[144]+ALGEBRAIC[145]);
ALGEBRAIC[149] =
ALGEBRAIC[145]/(ALGEBRAIC[142]+ALGEBRAIC[143]+ALGEBRAIC[144]+ALGEBRAIC[145]);
ALGEBRAIC[151] = ( 3.00000*( ALGEBRAIC[149]*ALGEBRAIC[140] -
ALGEBRAIC[146]*ALGEBRAIC[141])+ ALGEBRAIC[148]*ALGEBRAIC[137]) -
ALGEBRAIC[147]*ALGEBRAIC[134];
ALGEBRAIC[152] =  ALGEBRAIC[147]*CONSTANTS[150] -  ALGEBRAIC[146]*CONSTANTS[149];
ALGEBRAIC[153] =  (1.00000 -
CONSTANTS[60])*CONSTANTS[152]*ALGEBRAIC[150]*( CONSTANTS[8]*ALGEBRAIC[151]+
CONSTANTS[9]*ALGEBRAIC[152]);
ALGEBRAIC[205] = ( CONSTANTS[97]*ALGEBRAIC[25]*( STATES[3]*exp(ALGEBRAIC[28]) -
CONSTANTS[1]))/(exp(ALGEBRAIC[28]) - 1.00000);
ALGEBRAIC[104] = exp( - CONSTANTS[134]*1.00000*( pow(ALGEBRAIC[98], 1.0 / 2)/(1.00000+
pow(ALGEBRAIC[98], 1.0 / 2)) -  0.300000*ALGEBRAIC[98]));
ALGEBRAIC[107] =
( 1.00000*ALGEBRAIC[25]*( ALGEBRAIC[104]*STATES[3]*exp( 1.00000*ALGEBRAIC[28]) -
CONSTANTS[140]*CONSTANTS[1]))/(exp( 1.00000*ALGEBRAIC[28]) - 1.00000);
ALGEBRAIC[110] =  (1.00000 - CONSTANTS[54])*( (1.00000 -
ALGEBRAIC[90])*CONSTANTS[132]*ALGEBRAIC[107]*STATES[24]*( ALGEBRAIC[77]*(1.00000 -
STATES[33])+ STATES[29]*ALGEBRAIC[80]*STATES[33])+
ALGEBRAIC[90]*CONSTANTS[137]*ALGEBRAIC[107]*STATES[24]*( ALGEBRAIC[81]*(1.00000 -
STATES[33])+ STATES[29]*ALGEBRAIC[82]*STATES[33]));
ALGEBRAIC[208] = (STATES[4] - STATES[3])/CONSTANTS[105];
ALGEBRAIC[180] = 1.00000/(1.00000+pow(CONSTANTS[72]/STATES[2], 2.00000));
ALGEBRAIC[160] = 1.00000+
(CONSTANTS[1]/CONSTANTS[63])*(1.00000+1.00000/ALGEBRAIC[123]);
```

```
ALGEBRAIC[161] = CONSTANTS[1]/( CONSTANTS[63]*ALGEBRAIC[123]*ALGEBRAIC[160]);
ALGEBRAIC[164] =  ALGEBRAIC[161]*CONSTANTS[67];
ALGEBRAIC[154] = 1.00000+ (STATES[4]/CONSTANTS[63])*(1.00000+ALGEBRAIC[123]);
ALGEBRAIC[155] = ( STATES[4]*ALGEBRAIC[123])/( CONSTANTS[63]*ALGEBRAIC[154]);
ALGEBRAIC[167] =  ALGEBRAIC[155]*CONSTANTS[67];
ALGEBRAIC[157] = 1.00000+
(STATES[4]/CONSTANTS[61])*(1.00000+STATES[4]/CONSTANTS[62]);
ALGEBRAIC[158] =
( STATES[4]*STATES[4])/( ALGEBRAIC[157]*CONSTANTS[61]*CONSTANTS[62]);
ALGEBRAIC[170] =  ALGEBRAIC[158]*ALGEBRAIC[155]*CONSTANTS[65];
ALGEBRAIC[171] =  ALGEBRAIC[161]*CONSTANTS[154]*CONSTANTS[65];
ALGEBRAIC[162] = 1.00000/ALGEBRAIC[160];
ALGEBRAIC[163] =  ALGEBRAIC[162]*CONSTANTS[66];
ALGEBRAIC[165] = ALGEBRAIC[163]+ALGEBRAIC[164];
ALGEBRAIC[156] = 1.00000/ALGEBRAIC[154];
ALGEBRAIC[166] = ( ALGEBRAIC[156]*CONSTANTS[66])/ALGEBRAIC[122];
ALGEBRAIC[168] = ALGEBRAIC[166]+ALGEBRAIC[167];
ALGEBRAIC[159] = 1.00000/ALGEBRAIC[157];
ALGEBRAIC[169] =  ALGEBRAIC[159]*STATES[2]*CONSTANTS[68];
ALGEBRAIC[172] =  CONSTANTS[157]*ALGEBRAIC[168]*(ALGEBRAIC[170]+ALGEBRAIC[169])+
CONSTANTS[158]*ALGEBRAIC[170]*(CONSTANTS[157]+ALGEBRAIC[165]);
ALGEBRAIC[173] =  CONSTANTS[156]*ALGEBRAIC[170]*(ALGEBRAIC[168]+CONSTANTS[158])+
ALGEBRAIC[168]*ALGEBRAIC[169]*(CONSTANTS[156]+ALGEBRAIC[171]);
ALGEBRAIC[174] =  CONSTANTS[156]*ALGEBRAIC[165]*(ALGEBRAIC[170]+ALGEBRAIC[169])+
ALGEBRAIC[171]*ALGEBRAIC[169]*(CONSTANTS[157]+ALGEBRAIC[165]);
ALGEBRAIC[175] =  CONSTANTS[157]*ALGEBRAIC[171]*(ALGEBRAIC[168]+CONSTANTS[158])+
ALGEBRAIC[165]*CONSTANTS[158]*(CONSTANTS[156]+ALGEBRAIC[171]);
ALGEBRAIC[176] =
ALGEBRAIC[172]/(ALGEBRAIC[172]+ALGEBRAIC[173]+ALGEBRAIC[174]+ALGEBRAIC[175]);
ALGEBRAIC[177] =
ALGEBRAIC[173]/(ALGEBRAIC[172]+ALGEBRAIC[173]+ALGEBRAIC[174]+ALGEBRAIC[175]);
ALGEBRAIC[178] =
ALGEBRAIC[174]/(ALGEBRAIC[172]+ALGEBRAIC[173]+ALGEBRAIC[174]+ALGEBRAIC[175]);
ALGEBRAIC[179] =
ALGEBRAIC[175]/(ALGEBRAIC[172]+ALGEBRAIC[173]+ALGEBRAIC[174]+ALGEBRAIC[175]);
ALGEBRAIC[181] = ( 3.00000*( ALGEBRAIC[179]*ALGEBRAIC[170] -
ALGEBRAIC[176]*ALGEBRAIC[171])+ ALGEBRAIC[178]*ALGEBRAIC[167]) -
ALGEBRAIC[177]*ALGEBRAIC[164];
ALGEBRAIC[182] =  ALGEBRAIC[177]*CONSTANTS[157] -  ALGEBRAIC[176]*CONSTANTS[156];
ALGEBRAIC[183] =
CONSTANTS[60]*CONSTANTS[152]*ALGEBRAIC[180]*( CONSTANTS[8]*ALGEBRAIC[181]+
CONSTANTS[9]*ALGEBRAIC[182]);
ALGEBRAIC[85] = exp( - CONSTANTS[134]*1.00000*( pow(ALGEBRAIC[83], 1.0 / 2)/(1.00000+
pow(ALGEBRAIC[83], 1.0 / 2)) -  0.300000*ALGEBRAIC[83]));
```

```
ALGEBRAIC[88] =
( 1.00000*ALGEBRAIC[25]*( ALGEBRAIC[85]*STATES[4]*exp( 1.00000*ALGEBRAIC[28]) -
CONSTANTS[140]*CONSTANTS[1]))/(exp( 1.00000*ALGEBRAIC[28]) - 1.00000);
ALGEBRAIC[92] =  CONSTANTS[54]*( (1.00000 -
ALGEBRAIC[90])*CONSTANTS[132]*ALGEBRAIC[88]*STATES[24]*( ALGEBRAIC[77]*(1.00000 -
STATES[32])+ STATES[29]*ALGEBRAIC[80]*STATES[32])+
ALGEBRAIC[90]*CONSTANTS[137]*ALGEBRAIC[88]*STATES[24]*( ALGEBRAIC[81]*(1.00000 -
STATES[32])+ STATES[29]*ALGEBRAIC[82]*STATES[32]));
ALGEBRAIC[211] = (STATES[2] - STATES[9])/CONSTANTS[107];
ALGEBRAIC[213] = 1.00000/(1.00000+CONSTANTS[19]/ALGEBRAIC[49]);
ALGEBRAIC[215] =  CONSTANTS[110]*( (1.00000 - ALGEBRAIC[213])*STATES[41]+
ALGEBRAIC[213]*STATES[42]);
ALGEBRAIC[56] =
1.00000/(1.00000+( CONSTANTS[28]*CONSTANTS[29])/pow(CONSTANTS[29]+STATES[2],
2.00000)+( CONSTANTS[30]*CONSTANTS[31])/pow(CONSTANTS[31]+STATES[2], 2.00000));
ALGEBRAIC[101] = exp( - CONSTANTS[134]*4.00000*( pow(ALGEBRAIC[98], 1.0 / 2)/(1.00000+
pow(ALGEBRAIC[98], 1.0 / 2)) -  0.300000*ALGEBRAIC[98]));
ALGEBRAIC[106] =
( 4.00000*ALGEBRAIC[25]*( ALGEBRAIC[101]*STATES[9]*exp( 2.00000*ALGEBRAIC[28]) -
CONSTANTS[139]*CONSTANTS[2]))/(exp( 2.00000*ALGEBRAIC[28]) - 1.00000);
ALGEBRAIC[109] =  (1.00000 - CONSTANTS[54])*( (1.00000 -
ALGEBRAIC[90])*CONSTANTS[121]*ALGEBRAIC[106]*STATES[24]*( ALGEBRAIC[77]*(1.00000 -
STATES[33])+ STATES[29]*ALGEBRAIC[80]*STATES[33])+
ALGEBRAIC[90]*CONSTANTS[131]*ALGEBRAIC[106]*STATES[24]*( ALGEBRAIC[81]*(1.00000 -
STATES[33])+ STATES[29]*ALGEBRAIC[82]*STATES[33]));
ALGEBRAIC[112] = ALGEBRAIC[91]+ALGEBRAIC[109];
ALGEBRAIC[113] = ALGEBRAIC[92]+ALGEBRAIC[110];
ALGEBRAIC[114] = ALGEBRAIC[95]+ALGEBRAIC[111];
ALGEBRAIC[209] = ( CONSTANTS[99]*STATES[9])/(CONSTANTS[100]+STATES[9]);
ALGEBRAIC[207] =
( CONSTANTS[98]*4.00000*ALGEBRAIC[25]*( ALGEBRAIC[101]*STATES[9]*exp( 2.00000*ALG
EBRAIC[28]) -  CONSTANTS[139]*CONSTANTS[2]))/(exp( 2.00000*ALGEBRAIC[28]) - 1.00000);
ALGEBRAIC[210] =
(( CONSTANTS[104]*CONSTANTS[101])/(1.00000+CONSTANTS[103]/STATES[2]))*(STATES[0] -
CONSTANTS[114]);
ALGEBRAIC[212] =  (( (1.00000 -
CONSTANTS[104])*CONSTANTS[101])/(1.00000+CONSTANTS[103]/STATES[9]))*(STATES[0] -
CONSTANTS[114]);
ALGEBRAIC[214] = ALGEBRAIC[210]+ALGEBRAIC[212];
ALGEBRAIC[216] =  CONSTANTS[102]*(STATES[0] - CONSTANTS[114]);
ALGEBRAIC[217] = ( CONSTANTS[129]*0.00542500*STATES[9])/(STATES[9]+0.000920000);
ALGEBRAIC[218] =
( CONSTANTS[129]*2.75000*0.00542500*STATES[9])/((STATES[9]+0.000920000) -
0.000170000);
ALGEBRAIC[219] = 1.00000/(1.00000+CONSTANTS[19]/ALGEBRAIC[49]);
ALGEBRAIC[220] = ( 0.00488250*STATES[7])/15.0000;
```

```
ALGEBRAIC[221] =  CONSTANTS[111]*(( (1.00000 - ALGEBRAIC[219])*ALGEBRAIC[217]+
ALGEBRAIC[219]*ALGEBRAIC[218]) - ALGEBRAIC[220]);
ALGEBRAIC[53] =
1.00000/(1.00000+( CONSTANTS[113]*CONSTANTS[25])/pow(CONSTANTS[25]+STATES[9],
2.00000)+( CONSTANTS[26]*CONSTANTS[27])/pow(CONSTANTS[27]+STATES[9], 2.00000));
ALGEBRAIC[222] = (STATES[7] - STATES[8])/60.0000;
ALGEBRAIC[59] =
1.00000/(1.00000+( CONSTANTS[32]*CONSTANTS[33])/pow(CONSTANTS[33]+STATES[8],
2.00000));
}
```

- **Model training code**

```
import pandas as pd
import numpy as np
import random
import matplotlib.pyplot as plt
import xgboost as xgb
import joblib
import shap
import tensorflow as tf

from tensorflow.keras.models import Sequential
from tensorflow.keras.layers import Dense, LeakyReLU
from tensorflow.keras.optimizers import Adam, RMSprop
from tensorflow.keras.regularizers import l1_l2
from scikeras.wrappers import KerasClassifier
from sklearn.model_selection import GridSearchCV, train_test_split
from sklearn.metrics import accuracy_score
from natsort import natsorted
from sklearn.preprocessing import MinMaxScaler, StandardScaler
from itertools import cycle
from sklearn.metrics import roc_curve, auc
from sklearn.metrics import roc_auc_score
from sklearn.preprocessing import LabelEncoder, LabelBinarizer
from keras.models import load_model
from sklearn.metrics import precision_recall_fscore_support
from xgboost import XGBClassifier
from sklearn.metrics import confusion_matrix
from sklearn.metrics import f1_score
from sklearn.metrics import roc_curve, auc
from sklearn.metrics import accuracy_score, precision_score, recall_score, f1_score,
confusion_matrix
from sklearn.metrics import classification_report
from sklearn.preprocessing import LabelBinarizer
from tensorflow.keras.layers import Input, Dense, LeakyReLU
from tensorflow.keras.regularizers import l1_l2
from tensorflow.keras.optimizers import Adam,RMSprop
from tensorflow.keras.callbacks import ModelCheckpoint
from sklearn.model_selection import StratifiedKFold



path1 = 'D:/CiPA/'
path2 = 'Simulation/ANN 9 features multi data/'
```

```
lb = LabelBinarizer()
d_train = pd.read_csv(path1 + 'AVG_Tomek_4ch_training dataset.csv')
d_train["Sample"] = d_train['drug_name'].astype(str)

# Select the first 100 samples for each unique 'Drug_Name'
d_train = d_train.groupby('drug_name').head(2000)
d_train = d_train.drop(columns=['0.1 unnamed'])

data=d_train
df_train=data.iloc[:,:9].reset_index(drop=True)
label=data.loc[:, ['label']].reset_index(drop=True)

# Columns to be scaled
columns_to_scale = ['dVm_dt_Max','Vm_Resting', 'Ca_Diastole',
'qNet,'qInward','CaD50','CaD90','APD50','APD90']
scaler = StandardScaler()
df_train[columns_to_scale] = scaler.fit_transform(df_train[columns_to_scale])

############################### ANN architecture ################################

def merge_model():
   ApD90_input = Input(shape=(6))
   ApD90_model = ApD90_input

   dense1 = Dense(units=6, kernel_regularizer=l1_l2(l1=0.01, l2=0.01))(ApD90_model)
   leaky_relu1 = LeakyReLU(alpha=0.1)(dense1)

   dense2 = Dense(units=5, kernel_regularizer=l1_l2(l1=0.01, l2=0.01))(leaky_relu1)
   leaky_relu2 = LeakyReLU(alpha=0.1)(dense2)
   output = Dense(3, activation='softmax')( leaky_relu2)

   model = tf.keras.models.Model(inputs=[ApD90_model], outputs=[output])
   model.compile(loss='categorical_crossentropy', optimizer=RMSprop(learning_rate=0.001),
metrics=['accuracy'])

   return model

def model_fit(train, y_train, val_set,epoch = 100, batch = 128, checkpoint = False):
  '''
   checkpoint must be in string if used, otherwise model will not be saved
  '''
  import warnings

  model = merge_model()
  if type(checkpoint) == ModelCheckpoint:
   history = model.fit(train, y_train,
```

```
          validation_data = val_set,
          epochs        = epoch,
          batch_size     = batch,
          callbacks      = [checkpoint])

  else :
    warnings.warn('This model will NOT be saved, checkpoint should be in string(path) for
saving the model')
    history = model.fit(train, y_train,
          validation_data = val_set,
          epochs = epoch,
          batch_size = batch)

  return model, history


          fold = 10
          cv = StratifiedKFold(n_splits=fold, shuffle=True, random_state=42)

          # Initialize variables to store model history
          model_history = []

          # For reproducibility
          np.random.seed(13)
          tf.random.set_seed(13)

          i = 1  # Fold counter

          for train_idx, test_idx in cv.split(df_train, label['label']):
            # Training and validation data for this fold
            X_train_CA = df_train.iloc[train_idx]
            print('X_train_CA')
            X_val_CA = df_train.iloc[test_idx]

            y_train_CA = label.iloc[train_idx]
            y_val_CA = label.iloc[test_idx]

            # Training and validation samples for this fold
            sample_train_CA = data.iloc[train_idx]['Sample']
            sample_val_CA = data.iloc[test_idx]['Sample']

            # Print the fold number
            print(f"Fold - {i}")

            # Print the training data details
            print("Training data:")
```

```
        training_samples_count = sample_train_CA.value_counts()
        print(training_samples_count)
        print(f"Total training samples: {training_samples_count.sum()}")

        # Print the validation data details
        print("Validation data:")
        validation_samples_count = sample_val_CA.value_counts()
        print(validation_samples_count)
        print(f"Total validation samples: {validation_samples_count.sum()}")

        print("\n")  # Adds a blank line for clarity


        # Label Encoder (you can use LabelBinarizer or other encoding methods)
        binarizer = LabelBinarizer()
        y_train = binarizer.fit_transform(y_train_CA)
        y_val = binarizer.transform(y_val_CA)

        # Combine all training data (add other datasets as needed)
        X_train = [X_train_CA]
        val_dataset = (X_val_CA, y_val)

        # Define the path to save the best model
        detail = 'Model-epoch-{epoch:02d}-loss-{loss:.4f}-accuracy-{accuracy:.4f}-val_loss-
       {val_loss:.4f}-val_accuracy-{val_accuracy:.4f}.hdf5'
        save_path = path2 + 'fold/fold-{}/'.format(i) + detail

        # Create a ModelCheckpoint to save the best model
        save_model = ModelCheckpoint(save_path, monitor='val_loss', verbose=1,
       save_best_only=True)

        i += 1

        # Train the model for this fold
  model_history.append(model_fit(X_train, y_train, val_dataset, epoch=100, batch=128,
checkpoint=save_model))

for i in range(len(model_history)):

  plt.title('Train Accuracy vs Val Accuracy')

  plt.plot(model_history[i][1].history['accuracy'], label='Train Accuracy', color='red', )
  plt.plot(model_history[i][1].history['val_accuracy'], label='Val Accuracy', color='blue', )
  plt.ylabel("Accuracy")
  plt.xlabel("Epoch")
  plt.legend()
```

```
  plt.savefig(path2+'Accuracy/' + "Accuracy vs Val
  Accuracy_Li_Endo_Standart_{0}.png".format(i))
  plt.savefig("loss_model1 of kfold_10.jpg")
  plt.show()
```

```
for i in range(len(model_history)):

  plt.title('Train Loss vs Val Loss')

  plt.plot(model_history[i][1].history['loss'], label='Train Loss', color='red', )
  plt.plot(model_history[i][1].history['val_loss'], label='Val Loss', color='blue', )
  plt.ylabel("Loss")
  plt.xlabel("Epoch")
  plt.legend()
  plt.savefig(path2+'Loss/' + "Loss vs Val Loss_Li_Endo_Standart_{0}.png".format(i))
  plt.savefig("loss_model1 of kfold_10.jpg")
  plt.show()
```

- **Grid search hyperparameter optimization**

```
# Define the model creation function
def create_model(neurons=10, lr=0.001, batch_size=32, alpha=0.1, optimizer='adam',
layers=1, neurons_layer2=None):
    model = Sequential()
    model.add(Dense(neurons, kernel_regularizer=l1_l2(l1=0.01, l2=0.01),
input_shape=(9,)))
    model.add(LeakyReLU(alpha=alpha))
    if layers >= 2 and neurons_layer2 is not None:
        model.add(Dense(neurons_layer2, kernel_regularizer=l1_l2(l1=0.01, l2=0.01)))
        model.add(LeakyReLU(alpha=alpha))
    model.add(Dense(3, kernel_initializer='uniform', activation='softmax'))

    if optimizer == 'adam':
        optimizer = Adam(learning_rate=lr)
    elif optimizer == 'rmsprop':
        optimizer = RMSprop(learning_rate=lr)

    model.compile(loss='categorical_crossentropy', optimizer=optimizer,
metrics=['accuracy'])
    return model

# Initialize KerasClassifier with the create_model function
```

```
model = KerasClassifier(model=create_model, epochs=50, batch_size=32, verbose=0)

# Define the parameter grid
param_grid = {
   'model__neurons': [5, 6, 7, 8, 9],
   'model__lr': [0.001],
   'model__batch_size': [32, 64,128],
   'model__alpha': [0.1],
   'model__optimizer': ['adam', 'rmsprop'],
   'model__layers': [1, 2],
   'model__neurons_layer2': [5, 6, 7, 8, 9]
}

# Split the dataset into training and testing sets
X_train, X_test, y_train, y_test = train_test_split(df_train, Y_train, test_size=0.2,
random_state=42)

# Conduct the grid search
grid = GridSearchCV(estimator=model, param_grid=param_grid, n_jobs=-1, cv=10,
scoring='accuracy')
grid_result = grid.fit(X_train, y_train)

# Summarize results
print("Best: %f using %s" % (grid_result.best_score_, grid_result.best_params_))
best_model = grid_result.best_estimator_

# Predictions and accuracy evaluation
y_pred_train = best_model.predict(X_train)
y_pred_validation = best_model.predict(X_test)

accuracy_training = accuracy_score(y_train, y_pred_train)
accuracy_validation = accuracy_score(y_test, y_pred_validation)

print("Accuracy on train Set:", accuracy_training)
print("Accuracy on validation Set:", accuracy_validation)
```

- Model testing code

```
d_test = pd.read_csv('D:/CiPA/AVG_Tomek_4ch_testing dataset.csv')
d_test["Sample"] = d_test['drug_name'].astype(str) + "_" + d_test['Sample_ID'].astype(str)
# Group by 'Drug_Name' and select the first 100 samples\
d_test = d_test.groupby('drug_name', group_keys=False).apply(lambda x: x.head(2000))
d_test = d_test.drop(columns=['risk_code','drug_name', 'Sample_ID', 'dVm_dt_Repol',
'APDtri', 'Catri', 'Vm_Peak', 'Ca_Peak', 'qNet_Vm_repol_90'])
# d_test = d_test.drop(columns=['Sample_ID', 'Drug_Name'])
```

```
d_test = d_test.set_index('Sample')
d_test.index.name = None

val_test = d_test  #kalau hanya data testing, ubah jadi "d_test"
val_test.index.name = None
val_test=pd.DataFrame(val_test)
val_test


list_name_all = natsorted(val_test.index.to_list())
list_name = natsorted(set([x.split('_')[0] for x in list_name_all]))


dict_drug = {}

for uniq_name in list_name:
  drug_pairs = np.empty((0,1))
  for qni_name in list_name_all:

    id_qni, sample_qni = qni_name.split('_')[0], qni_name.split('_')[1]

    if uniq_name == id_qni : # begin with astemizole

      pairs = np.array([qni_name])
      drug_pairs = np.vstack((drug_pairs, pairs))

  dict_drug[uniq_name] = drug_pairs

for i in list_name:
  print(i)
  print(dict_drug[i].shape)

unique_pairs = np.array([])
  for i in range(10000):

    limit = range( 0, len( dict_drug['astemizole'] ) )
    temp_pair = np.unique(random.sample(limit, 16))

    if  unique_pairs.any():

      if (temp_pair == unique_pairs).all():
        print(temp_pair)

      unique_pairs = np.vstack((unique_pairs, temp_pair))

    else :
```

```
    unique_pairs = temp_pair

for i in range(200):
  print(dict_drug['astemizole'][11])
  break

  df_qni = val_test.iloc[:,:9]
  #df_qni = val_qni.iloc[:, :-2]
  df_label = val_test.loc[:, ['label']]
  df_qni.reset_index()

  scaler = StandardScaler()
  # Fit and transform the scaler on the data
  df_qni_scaled = scaler.fit_transform(df_qni)
  # Convert the scaled array back to a DataFrame
  df_qni_scaled = pd.DataFrame(df_qni_scaled, columns=df_qni.columns)
  # Reassign the index to the scaled DataFrame
  df_qni_scaled.index = df_qni.index


  def auc_all(Y_test, predictions):
    fpr = dict()
    tpr = dict()
    roc_auc = dict()
    for i in range(3):
        fpr[i], tpr[i], _ = roc_curve(Y_test[:, i], predictions[:, i])
        roc_auc[i] = auc(fpr[i], tpr[i])

    # Compute micro-average ROC curve and ROC area
    fpr["micro"], tpr["micro"], _ = roc_curve(Y_test.ravel(), predictions.ravel())
    roc_auc["micro"] = auc(fpr["micro"], tpr["micro"])


    # First aggregate all false positive rates
    lw = 2

    all_fpr = np.unique(np.concatenate([fpr[i] for i in range(3)]))

    # Then interpolate all ROC curves at this points
    mean_tpr = np.zeros_like(all_fpr)
    for i in range(3):
        mean_tpr += np.interp(all_fpr, fpr[i], tpr[i])

    # Finally average it and compute AUC
    mean_tpr /= 2
```

```
    fpr["macro"] = all_fpr
    tpr["macro"] = mean_tpr
    roc_auc["macro"] = auc(fpr["macro"], tpr["macro"])

    return (fpr, tpr, roc_auc)


  model_test = load_model('D:/ANN 9 features multi data/fix result/1Net CL standard scaller data baru 4 ion channel minmax 2 gs opt/fold/fold-1/model terbaik/Model-epoch-100-loss-0.1208-accuracy-0.9714-val_loss-0.1128-val_accuracy-0.9775.hdf5')

  ########################### 10,000 time tests #################################

  like_p = []
  like_m = []

  idx = 0
  final_pairs_qni = np.empty((0,9), dtype='float32')

  final_label = np.empty((0,1), dtype='int32')

  result_df = pd.DataFrame()
  stop_flow = 0

  for id_pair, pair in enumerate(unique_pairs):

    # ===========================================
    #        Inner Loop / Drug_name
    # ===========================================
    for drug in dict_drug.keys():
      qni = dict_drug[drug][pair[idx]][0]

      X_qni = df_qni_scaled.loc[[qni]]

      Y_qni = df_label.loc[[qni], ['label']]


      final_pairs_qni = np.vstack((final_pairs_qni, X_qni))
      final_label = np.vstack((final_label, Y_qni))

      idx += 1
    # ===========================================
    #       End of  Inner Loop/ Drug_name
    # ===========================================

  # ===========================================
```

```
#      Outer Loop / Pairs
# ============================================

   lb = LabelBinarizer()
   Y_test = lb.fit_transform(final_label)
   input_data = final_pairs_qni.astype('float32')
   recall_score(predictions.astype('float64').argmax(axis=1), Y_test.argmax(axis=1),
average='weighted')
 Y_test.argmax(axis=1))
   u = 1e-6
   sd = 1e-12
   cfx = confusion_matrix(Y_test.argmax(axis=1), predictions.argmax(axis=1))

   tp_high = cfx[0,0]
   tn_high = cfx[1,1]+cfx[1,2]+cfx[2,1]+cfx[2,2]
   fp_high = cfx[1,0]+cfx[2,0]
   fn_high = cfx[0,1]+cfx[0,2]

   tp_inter = cfx[1,1]
   tn_inter = cfx[0,0]+cfx[0,2]+cfx[2,0]+cfx[2,2]
   fp_inter = cfx[0,1]+cfx[2,1]
   fn_inter = cfx[1,0]+cfx[1,2]

   tp_low = cfx[2,2]
   tn_low = cfx[0,0]+cfx[0,1]+cfx[1,0]+cfx[1,1]
   fp_low = cfx[0,2]+cfx[1,2]
   fn_low = cfx[2,0]+cfx[2,1]

   acc_high = (tp_high+tn_high)/(tp_high+tn_high+fp_high+fn_high)
   pre_high = tp_high/(tp_high+fp_high)
   rec_high = tp_high/(tp_high+fn_high)
   spe_high = tn_high/(tn_high+tp_high)
   lrp_high = (rec_high + np.random.normal(u, sd)) / (1 - spe_high + np.random.normal(u, sd))
   lrn_high = (1 - rec_high + np.random.normal(u, sd)) / (spe_high + np.random.normal(u, sd))
   f1s_high = (2*pre_high*rec_high)/(pre_high+rec_high)

   acc_inter = (tp_inter+tn_inter)/(tp_inter+tn_inter+fp_inter+fn_inter)
   pre_inter = tp_inter/(tp_inter+fp_inter)
   rec_inter = tp_inter/(tp_inter+fn_inter)
   spe_inter = tn_inter/(tn_inter+tp_inter)
   lrp_inter = (rec_inter + np.random.normal(u, sd)) / (1 - spe_inter + np.random.normal(u,
sd))
   lrn_inter = (1 - rec_inter + np.random.normal(u, sd)) / (spe_inter + np.random.normal(u,
sd))
   f1s_inter = (2*pre_inter*rec_inter)/(pre_inter+rec_inter)
```

```
    acc_low = (tp_low+tn_low)/(tp_low+tn_low+fp_low+fn_low)
    pre_low = tp_low/(tp_low+fp_low)
    rec_low = tp_low/(tp_low+fn_low)
    spe_low = tn_low/(tn_low+tp_low)
    lrp_low = (rec_low + np.random.normal(u, sd)) / (1 - spe_low + np.random.normal(u, sd))
    lrn_low = (1 - rec_low + np.random.normal(u, sd)) / (spe_low + np.random.normal(u, sd))
    f1s_low = (2*pre_low*rec_low)/(pre_low+rec_low)
    acc = (acc_high+acc_inter+acc_low)/3
    (_, _, roc_auc) = auc_all(Y_test, predictions) # (fpr, tpr, roc_auc) if fpr and tpr needed

    col_data = {
      'sample_pairs': id_pair + 1,
      'pairs': [[pair]],
      'acc': [f"{acc:.4f}"],
      'acc_high': [f"{acc_high:.4f}"],
      'acc_inter': [f"{acc_inter:.4f}"],
      'acc_low': [f"{acc_low:.4f}"],
      'precision_high': [f"{pre_high:.4f}"],
      'precision_inter': [f"{pre_inter:.4f}"],
      'precision_low': [f"{pre_low:.4f}"],
      'recall/sensitivity_high': [f"{rec_high:.4f}"],
      'recall/sensitivity_inter': [f"{rec_inter:.4f}"],
      'recall/sensitivity_low': [f"{rec_low:.4f}"],
      'specificity_high': [f"{spe_high:.4f}"],
      'specificity_inter': [f"{spe_inter:.4f}"],
      'specificity_low': [f"{spe_low:.4f}"],
      'LR+_high': [f"{lrp_high:.4f}"],
      'LR+_inter': [f"{lrp_inter:.4f}"],
      'LR+_low': [f"{lrp_low:.4f}"],
      'LR-_high': [f"{lrn_high:.4f}"],
      'LR-_inter': [f"{lrn_inter:.4f}"],
      'LR-_low': [f"{lrn_low:.4f}"],
      'f1_score_high': [f"{f1s_high:.4f}"],
      'f1_score_inter': [f"{f1s_inter:.4f}"],
      'f1_score_low': [f"{f1s_low:.4f}"],
      'high_auc': [f"{roc_auc[0]:.4f}"],
      'inter_auc': [f"{roc_auc[1]:.4f}"],
      'low_auc': [f"{roc_auc[2]:.4f}"]
  }

    res_detail = pd.DataFrame(col_data)
    result_df = pd.concat([result_df, res_detail], axis=0, ignore_index=True)
    if idx == 16:
        idx = 0
        final_pairs_qni = np.empty((0,9), dtype='float32')
        final_label = np.empty((0,1), dtype='int32')
```

```
  if stop_flow == 10000:
    break
# ============================================
#      End of Outer Loop / Pairs
# ============================================
```
